# UCHL1 Promotes Gastric Cancer Progression by Regulating CIP2A Degradation

**DOI:** 10.3390/ph18101468

**Published:** 2025-09-29

**Authors:** Ga-ye Lee, In-ho Jeong, Byung Sik Kim, Hee-Sung Kim, Peter Chang-Whan Lee

**Affiliations:** 1 Department of Biochemistry & Molecular Biology, Asan Medical Center, University of Ulsan College of Medicine, Seoul 05505, Republic of Korea; a51008964@gmail.com (G.-y.L.); d4inno@naver.com (I.-h.J.); 2 Department of General Surgery, Hallym Medical Center, Hallym University College of Medicine, Anyang 14068, Republic of Korea; amcgs009@gmail.com

**Keywords:** ubiquitin proteasome system, UCHL1, gastric cancer, CIP2A, cell cycle

## Abstract

**Background:** Gastric cancer is one of the most prevalent malignancies worldwide and the fourth leading cause of cancer-related mortality. Protein ubiquitination and deubiquitination regulate protein stability as post-translational modifications, playing essential roles in tumorigenesis. Although UCHL1, a deubiquitinating enzyme (DUB), is implicated in the progression of several cancer types, its role in gastric cancer remains unclear. **Methods:** Kaplan–Meier analysis and gastric cancer patient tissues were used to assess UCHL1 expression. Cell viability assay, colony-forming assay, and transwell migration and invasion assay were performed to evaluate cell growth. Immunoprecipitation and Western blotting analyzed protein expression and interactions. **Results:** This study demonstrates that UCHL1 expression is markedly upregulated in gastric cancer tissues compared to normal tissues. Elevated UCHL1 expression is associated with poor patient prognosis, supporting its potential role as an oncogenic factor. Reduced UCHL1 expression suppressed cell proliferation, migration, and invasion in gastric cancer cell lines. As the underlying mechanism, we identified CIP2A, a known oncogenic regulator of c-Myc, as a downstream effector of UCHL1. UCHL1 knockdown reduced CIP2A protein levels via deubiquitination, attenuated c-Myc signaling, and decreased expression of key cell cycle regulators. Furthermore, UCHL1 knockdown significantly downregulated cyclin D1 expression, arresting the cell cycle in the G1 phase and inhibiting cell proliferation. **Conclusions:** Collectively, our findings reveal that UCHL1 promotes gastric cancer progression, highlighting it as a potential therapeutic target.

## 1. Introduction

Gastric cancer (GC), as the fifth most diagnosed malignancy, is the fourth leading cause of cancer-related deaths globally, accounting for >750,000 deaths [1]. According to Lauren’s classification, GC is a heterogeneous disease that is divided into two histological types: intestinal and diffuse [2,3]. The intestinal subtype, which occurs more frequently than the diffuse subtype, is associated with a better prognosis [2]. Multiple risk factors for GC, including environmental factors and genetic alterations, have been reported in numerous studies [2,3,4]. However, GC caused by dysregulated molecular pathways remains poorly characterized [3]. Therefore, elucidating the initiating mechanisms is necessary for understanding GC pathogenesis and biological characteristics [5].

The ubiquitin-proteasome system (UPS) is responsible for post-translational modification and proteostasis, known as protein homeostasis, which maintains the appropriate protein function in eukaryotic cells [6]. Ubiquitination of misfolded or unfolded intracellular proteins is required to prevent the development of various disorders [7]. Similarly, substrate proteins from which ubiquitin chains are removed through deubiquitination play an essential role in regulating proteostasis, protein activity, and cellular signaling pathways [8]. Recently, deubiquitinating enzymes (DUBs) have been classified into six cysteine proteases/one metalloprotease, comprising approximately 100 members [9]. Specifically, DUBs regulate various aspects of cellular response, including cell growth, apoptosis, gene expression, localization, and differentiation [9,10]. Additionally, they regulate various tumor-related intracellular signaling pathways, such as the Wnt/beta-catenin cascade, NF-κB, and RAS signaling, in different tumors [10,11,12,13]. For example, BRCA-1-associated protein 1 (BAP1), a DUB, plays a role as a tumor suppressor in pancreatic cancer by inhibiting NF-κB signaling [14]. Consequently, investigating the functions of DUBs within molecular pathways in cancer cells is essential for overcoming various human diseases [3].

Ubiquitin C-terminal hydrolase-L1 (UCHL1), a member of ubiquitin C-terminal hydrolases (UCHs), is an underexplored DUB that is predominantly expressed in the brain [15]. Based on prior studies, UCHL1 is a bifunctional regulator, acting either as a tumor suppressor or promoter depending on the tumor type [15,16]. Specifically, UCHL1 acts as a tumor suppressor by stabilizing p53 in breast cancer and hepatocellular carcinoma, whereas it acts as a tumor promoter by enhancing the PI3K/Akt signaling pathway in non-small cell lung cancer and melanoma [17,18,19,20]. However, studies on the molecular mechanism and role of UCHL1 in gastric cancer are very limited [3]. Accordingly, it is important to identify the role and mechanism of UCHL1 in gastric cancer progression.

Cancerous inhibitor of protein phosphatase 2A (CIP2A), a well-known oncoprotein that inhibits tumor suppressor protein phosphatase 2A (PP2A), is overexpressed in about 70% of various cancer types, such as non-small cell lung cancer (NSCLC), GC, and breast cancer [21,22,23,24,25,26]. CIP2A promotes tumorigenesis by modulating key regulators of cancer cell proliferation and survival, including PP2A, c-Myc, and E2F1 [27]. Although PP2A normally acts as a tumor suppressor by dephosphorylating c-Myc, CIP2A maintains c-Myc phosphorylation and promotes its overexpression by binding PP2A and inhibiting its activity, which ultimately drives tumor growth [27,28]. Furthermore, CIP2A functions as a specific regulatory factor in various tumor types. For example, CIP2A activates the Wnt/β-catenin and JNK pathways or binds to Raf in the Ras signaling cascade, thereby contributing to tumorigenesis [28]. Consistent with its oncogenic properties, CIP2A also plays a tumor-promoting role in GC, although the underlying molecular mechanisms remain incompletely understood [29].

This study shows that UCHL1 plays an oncogenic role in GC compared to normal tissues, being upregulated in >70% of cases. Additionally, UCHL1 was related to the overall survival of patients with GC. *UCHL1* knockdown using siRNA significantly suppressed cancer-associated phenotypes, including cell proliferation, migration, and invasion. Mechanistically, UCHL1 interacted with CIP2A as a DUB, thereby promoting tumor progression in GC. Similarly, treatment with LDN-57444, a specific UCHL1 inhibitor, also suppressed tumor growth. Hence, UCHL1 promotes tumor growth, suggesting it as a potential therapeutic target in cancer therapy.

## 2. Results

### 2.1. UCHL1 Is Highly Expressed in Gastric Cancer Tissues

**Figure 1:**
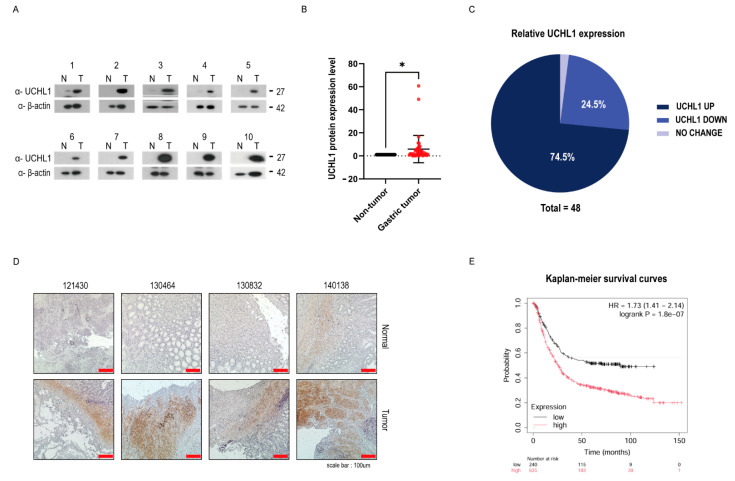
UCHL1 is highly expressed in gastric cancer tissues. (**A**) Representative immunoblot for UCHL1 expression in 48 paired gastric cancer tissues and normal gastric cancer tissues. N refers to the meaning of normal tissue; T refers to the meaning of tumor. (**B**) Immunoblot analysis was performed to quantify UCHL1 protein levels in 48 matched pairs of gastric cancer tissues and normal gastric tissues. Black dots represent normal tissues, while red dots represent gastric cancer tissues. The error bar represents an SD value. The two-sided Student’s *t*-test was used for statistical analysis (* *p* < 0.05). (**C**) The circular chart shows the ratios of each UCHL1 protein expression pattern (dark blue: T > N; sky blue: N > T; light blue: no significant difference). (**D**) Immunohistochemical analysis of gastric cancer tissues and normal gastric tissues was performed using anti-UCHL1 antibodies. The red line represents scale bar. Scale bar: 100 μm. (**E**) Kaplan–Meier analysis of the overall survival in gastric cancer patients from different UCHL1 protein expressions (high: red line; low: black line).

The role of UCHL1 in GC has not been well elucidated [3]. First, we identified the expression level of UCHL1 to investigate the biological function of UCHL1 in GC. We analyzed UCHL1 protein levels in 48 pairs of GC tissues from patients with normal gastric tissues using Western blotting (Figure 1A and Figure S1). UCHL1 protein was highly expressed in about 70% of GC samples compared to normal tissues (Figure 1B,C). Furthermore, we conducted an immunohistochemical analysis using antibodies against UCHL1 to further examine its expression. As observed in Figure 1A,D, we confirmed a significantly upregulated protein expression of UCHL1 in GC compared to normal tissues (Figure 1A,D). Additionally, elevated UCHL1 expression was associated with poor overall survival in patients with GC through Kaplan–Meier survival analysis (Kaplan–Meier plotter, http://kmplot.com/analysis/, accessed on 9 July 2025) (Figure 1E). Notably, even among patients with low UCHL1 expression, tumor stage and Lauren classification remained significantly associated with clinical prognosis (Figure S2). Thus, UCHL1 plays an oncogenic role in GC and is correlated with poor clinical outcomes.

### 2.2. UCHL1 Knockdown Suppresses Cell Growth in Gastric Cancer Cells

**Figure 2:**
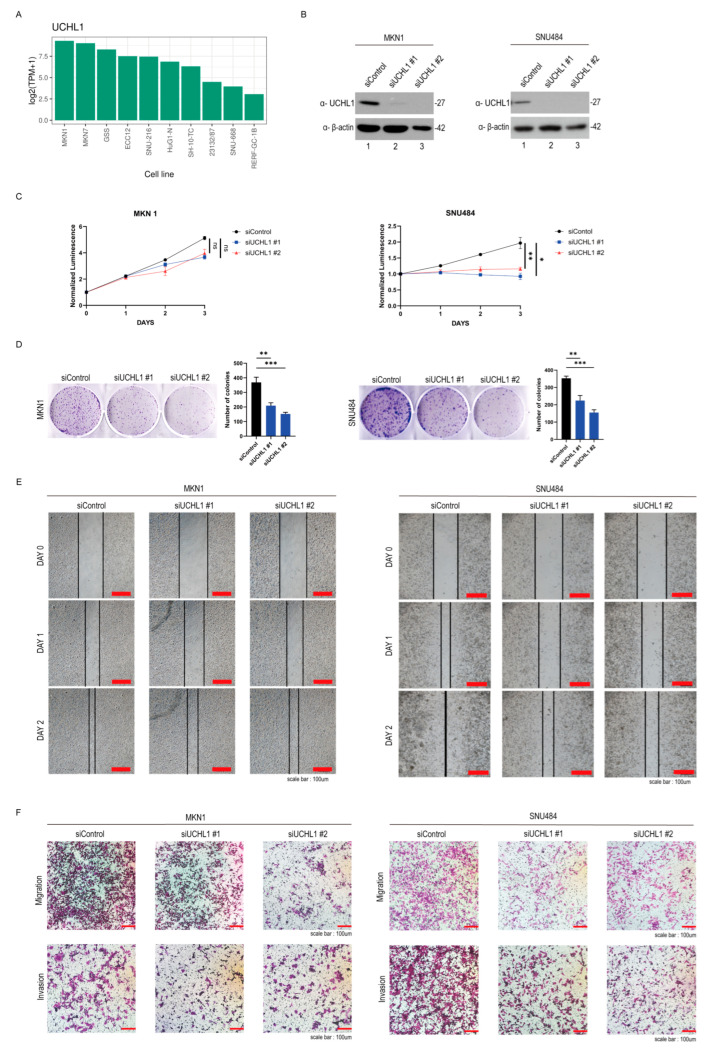
UCHL1 knockdown suppresses cell growth in gastric cancer cells. (**A**) The mRNA expression of UCHL1 was analyzed in multiple gastric cancer cell lines using the ShinyThor database. (**B**) Western blot analysis of UCHL1 in MKN1 and SNU484 cells at 48 h after transfection with two different siRNAs. (**C**) The effect of UCHL1 knockdown on cell viability in MKN1 and SNU484 cells. Both cells were seeded in 96-well white bottom dishes and monitored over 3 days using a Cell Titer-Glo luminescence assay (*n* = 5, normalized to day 0). The error bar represents the SD value. The two-sided Student’s *t*-test was used for statistical analysis (* *p* < 0.05). (ns, not significant; * *p* < 0.05, ** *p* < 0.001). (**D**) Colony formation of MKN1 and SNU484 gastric cancer cells following the silencing of UCHL1. Transfected with or without cells using siUCHL1s were incubated for at least 7 days. The quantitative data was analyzed by a two-sided Student’s *t*-test (** *p* < 0.001, *** *p* < 0.0001). (**E**) The effects of the UCHL1 knockdown on the potential of migration by using a wound healing assay. Cell migration was assessed in control and *UCHL1*-silenced cells at each time point. (24, 48, and 72 h) (**F**) The effect of knockdown of UCHL1 on the potential of migratory and invasive ability in MKN1 and SNU484 cells. Transwell inserts were covered with Matrigel-coated membranes in the case of the invasion assay. The migratory potential was assessed after a 36 h incubation, and the invasive potential was assessed after a 24 h incubation.

To investigate the function of UCHL1, which is highly expressed in gastric cancer according to ShinyThor (https://doi.org/10.1093/bioadv/vbaf061, accessed on 7 September 2025) and Human Protein Atlas (https://www.proteinatlas.org/, accessed on 7 September 2025) databases, we performed knockdown experiments using MKN1 and SNU484 cell lines, both of which show high levels of UCHL1 expression (Figure 2A and Figure S3). We evaluated the cell growth in response to *UCHL1* knockdown by siUCHL1 in MKN1 and SNU484 cells to further explore the oncogenic role of UCHL1 in GC cells (Figure 2B). Reduced UCHL1 expression through siRNA transfection significantly decreased cell proliferation in MKN1 and SNU484 cells (Figure 2C,D). Afterward, we performed wound-healing and transwell migration assays to assess the cell migration property as a characteristic of tumorigenesis. *UCHL1* knockdown substantially suppressed cell migration in MKN1 and SNU484 cells (Figure 2E,F). Consistent with the migration assay in Figure 2E,F, UCHL1 inhibition in the cell invasion assay showed that the cell invasiveness was decreased in MKN1 and SNU484 cells (Figure 2F). Overall, these results collectively support the role of UCHL1 in GC, revealing its impact on cell growth.

### 2.3. UCHL1 Interacts with and Deubiquitinates CIP2A

**Figure 3:**
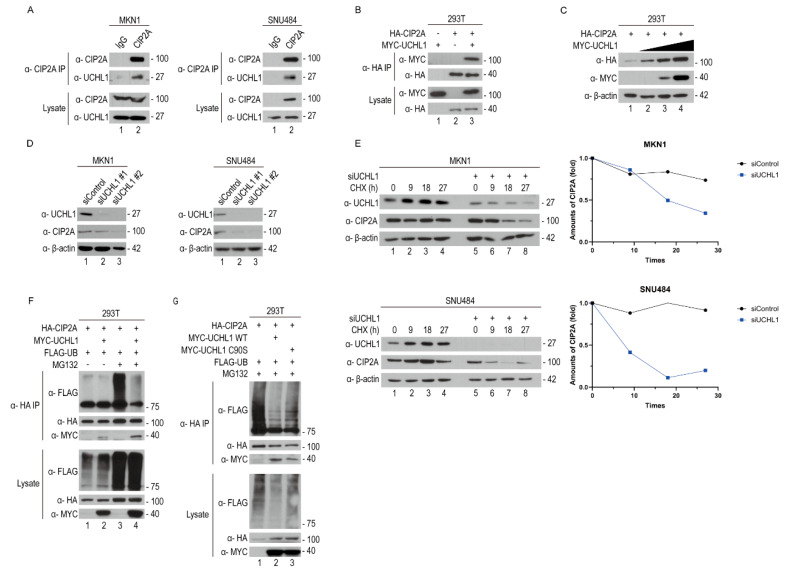
UCHL1 interacts with and deubiquitinates CIP2A. (**A**) Lysates from MKN1 and SNU484 cells were lysed and immunoprecipitated with control mouse IgG or antibodies against CIP2A. Western blot analysis was then performed using antibodies against UCHL1 and CIP2A. (**B**) Lysates from HEK293T cells, which were transfected with HA-CIP2A and MYC-UCHL1 plasmids, were lysed and immunoprecipitated with HA-antibody–conjugated beads. (**C**) HA-CIP2A protein levels increased in a dose-dependent manner upon MYC-UCHL1 overexpression. HEK 293T cells were transfected with a fixed amount (1 μg) of HA-CIP2A and a gradient (from 0.3 to 3 μg) of MYC-UCHL1 constructs. Lysates of HEK293T cells were lysed and immunoblotted with the indicated antibodies. (**D**) Western blot analysis of MKN1 and SNU484 cells following the silencing of UCHL1. (**E**) Cycloheximide (CHX) chase assays were performed in cells transfected with Control or siUCHL1 to analyze the half-life of CIP2A. (**F**) Lysates from HEK293T cells, which were transfected with HA-CIP2A, MYC-UCHL1 WT, and FLAG-ubiquitin plasmids. After 12 h of MG132 treatment prior to lysis, the cell lysates were lysed and immunoprecipitated with HA-antibody-conjugated beads. Western blot analysis was then performed using antibodies against the tag. (**G**) Lysates from HEK293T cells, which were transfected with the same amount of MYC-UCHL1 and MYC-UCHL1 C90S plasmids, were lysed and immunoprecipitated with HA-antibodies-conjugated beads. Western blot analysis was then performed using antibodies as shown.

We investigated the UCHL1-associated proteins using the Biogrid database (BioGRID, http://thebiogrid.org/, accessed on 17 June 2025) to understand the molecular mechanism by which UCHL1 facilitates GC cell progression. Furthermore, CIP2A, which is expressed in multiple cancer types, including gastric cancer, has been reported to regulate key proliferation pathways such as AKT. Its elevated expression may contribute to tumor progression and influence patient prognosis, suggesting a potential interaction with UCHL1 [29]. Based on data from the Biogrid database (BioGRID, http://thebiogrid.org/, accessed on 17 June 2025), CIP2A (encoded by the *KIAA1524* gene) was identified as a high-confidence physical interactor of UCHL1, as supported by mass spectrometry data. We also confirmed the interaction between CIP2A and UCHL1 using CIP2A interaction proteomics (Table S1). To validate the physical interaction with UCHL1, we analyzed endogenous CIP2A and UCHL1 expression using an endogenous-immunoprecipitation (endo-IP) assay with anti-CIP2A, identifying their interaction in MKN1 and SNU484 cells (Figure 3A). Then, we examined the interaction of UCHL1 with CIP2A using a co-immunoprecipitation (co-IP) assay in 293T cells overexpressing tagged plasmids. As observed in the endo-IP assay, we confirmed the interaction between UCHL1 and CIP2A in the co-IP assay as well (Figure 3B). Furthermore, as UCHL1 was overexpressed in a dose-dependent manner, CIP2A protein expression was correspondingly increased depending on UCHL1 expression (Figure 3C). Additionally, as UCHL1 was downregulated using transfected siRNAs, CIP2A protein expression decreased (Figure 3D). Therefore, UCHL1 interacts with CIP2A and regulates its expression.

Following the above-provided results, we identified CIP2A as both a binding partner and substrate of UCHL1. Hence, we hypothesized that UCHL1 regulates CIP2A through a mechanism dependent on UPS. Consequently, we conducted a cycloheximide (CHX) chase assay in UCHL1-depleted MKN1 and SNU484 cells to confirm whether UCHL1 regulates the stability of CIP2A through its deubiquitinating enzyme activity. We discovered that UCHL1 knockdown reduced the half-life of CIP2A protein (Figure 3E). Then, we performed a ubiquitination assay in 293T cells to verify whether CIP2A was indeed regulated by the UPS with UCHL1. Upon MG132 treatment, UCHL1 decreased the ubiquitination of CIP2A in 293T cells, indicating that UCHL1 functions as a DUB for CIP2A (Figure 3F). Additionally, we found that the catalytic mutant form of UCHL1 (UCHL1 C90S), which lacks the enzymatic capacity to remove ubiquitin, increased the ubiquitination of CIP2A (Figure 3G). Therefore, UCHL1 binds to CIP2A and acts as its DUB, regulating its stability via the UPS.

### 2.4. UCHL1 Regulates Cyclin D1 Through the CIP2A-Mediated Signaling Pathway

**Figure 4:**
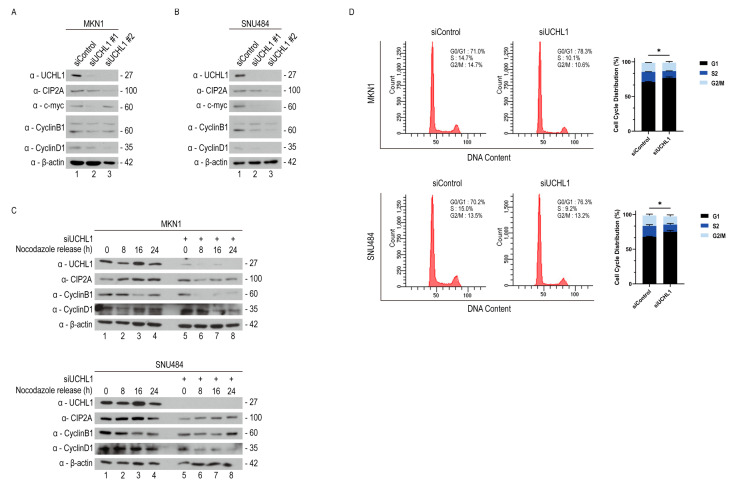
UCHL1 regulates cycling D1 through the CIP2A-mediated signaling pathway. (**A**) Western blot analysis of MKN1 following the silencing of UCHL1. (**B**) Western blot analysis of SNU484 cells following the silencing of UCHL1. (**C**) Effects of the UCHL1 knockdown on cell cycle regulatory protein following 16 h nocodazole treatment in MKN1 and SNU484 cells, after which the medium was replaced with complete growth medium to allow a cell cycle re-entry. (**D**) Flow cytometric analysis of the G1 phase cell cycle arrest following UCHL1 knockdown in MKN1 and SNU484 cells. The error bar represents SD. The two-sided Student’s *t*-test was used for statistical analysis (* *p* < 0.05; *n* = 4).

A previous study indicated that CIP2A stabilizes oncogenic transcription factor c-Myc by inhibiting its PP2A-mediated dephosphorylation and subsequent degradation [30]. Continuously, c-Myc directly promotes cell cycle progression by upregulating cyclins and other cell cycle regulators, including cyclin D1 and cyclin B1 [31]. Thus, we hypothesized that UCHL1 promotes GC cell cycle progression through the UCHL1-CIP2A-c-Myc-cyclin D1 axis. To confirm this hypothesis, we analyzed immunoblotting of c-Myc, cyclin B1, and cyclin D1 in MKN1 and SNU484 cells following *UCHL1* knockdown. c-Myc, cyclin B1, and cyclin D1 expression were markedly reduced in both cell lines (Figure 4A). Consistent with this, *UCHL1* knockdown induced cell cycle arrest at the G1 phase due to the downregulated cyclin D1. To further validate this finding, we conducted a nocodazole release assay, confirming that cyclin D1 levels were decreased in siUCHL1-transfected MKN1 and SNU484 cells (Figure 4B). Additionally, we revealed that the population of cells in the G1 phase following UCHL1 silencing increased compared to the control group using flow cytometry analysis with PI staining (Figure 4C). Hence, UCHL1 modulates the cell cycle by regulating CIP2A degradation.

### 2.5. LDN-57444 Treatment Resulted in the Cell Cycle Arrest in Gastric Cancer Cells

**Figure 5:**
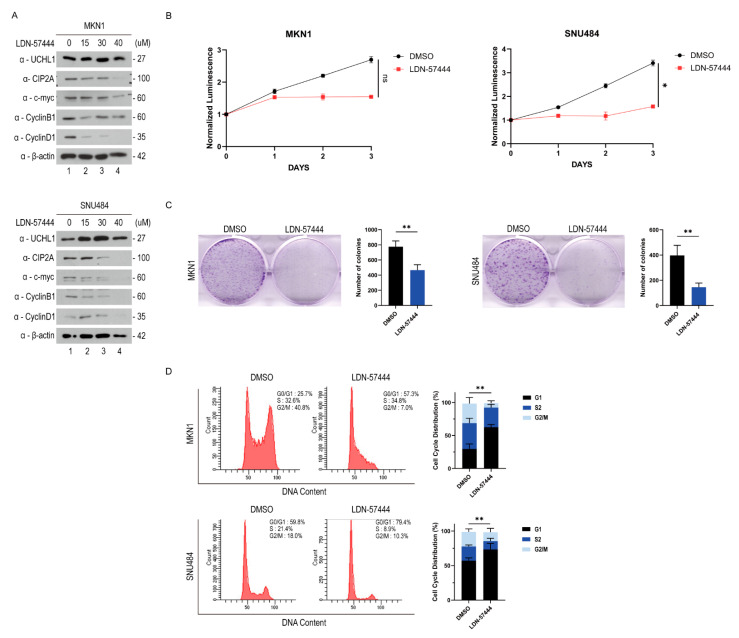
LDN-57444 treatment resulted in cell cycle arrest in gastric cancer cells. (**A**) MKN1 and SNU484 cells were treated with LDN-57444 for 48 h and immunoblotted with the indicated antibodies. (**B**) The inhibition of UCHL1 with LDN-57444 on cell viability in MKN1 and SNU484. The error bar represents SD. The two-sided Student’s *t*-test was used for statistical analysis (ns, not significant, * *p* < 0.05; *n* = 5, with normalization at day 0). (**C**) Effects of UCHL1 inhibition by LDN-57444 on cell proliferation in MKN1 and SNU484 cells. Cells were treated with or without LDN-57444 for at least 7 days. The error bar represents SD. The two-sided Student’s *t*-test was used for statistical analysis (** *p* < 0.05; n = 3). (**D**) Analyzed the G1 phase cell cycle arrest induced by a UCHL1 inhibitor, LDN-57444, using flow cytometric analysis. The error bar represents SD. The two-sided Student’s *t*-test was used for statistical analysis (** *p* < 0.01; *n* = 4).

Although numerous studies have demonstrated that LDN-57444, a UCHL1 inhibitor, suppresses cell proliferation, we conducted further analyses to determine whether it was mediated through the UCHL1-CIP2A-c-Myc-cyclin D1 axis, which we specifically identified in GC [32,33,34]. We examined CIP2A, c-Myc, and cyclin D1 protein expression in MKN1 and SNU484 cells treated with increasing doses of LDN-57444 to elucidate the downstream mechanisms underlying the inhibitory effects of LDN-57444 on the GC cell growth. In a dose-dependent manner, the protein expression of CIP2A, c-Myc, cyclin B1, and cyclin D1 was progressively reduced with an increasing concentration of LDN/57444, indicating that pharmacological inhibition of UCHL1 activity induces the G1 phase cell cycle arrest in GC cells, similar to the effect observed with *UCHL1* knockdown by siRNA (Figure 5A). Furthermore, we confirmed the effect of UCHL1 inhibition by LDN-57444 on cell proliferation in MKN1 and SNU484 cells. Treatment with LDN-57444 significantly reduced cell proliferation in both cell lines (Figure 5B). Additionally, we identified that the population of cells in the G1 phase was accumulated in the LDN-57444-treated group against the DMSO control using flow cytometry analysis with PI staining (Figure 5C). Therefore, UCHL1 regulates the cell cycle. Both physiological and pharmacological inhibition of UCHL1 suppress GC cell growth, indicating its potential as a therapeutic marker in GC.

## 3. Discussion

Our study reveals novel insight into the oncogenic role of UCHL1 in GC. Among various DUBs implicated in tumorigenesis, UCHL1 is relatively understudied, with its function in GC remaining poorly characterized [3]. In this study, we showed that UCHL1 expression was notably elevated in gastric tumors compared with normal gastric tissues. Additionally, high UCHL1 expression was significantly associated with patient survival in GC. Nevertheless, although the number of patient samples in our study was limited, the concordance with survival analyses based on Lauren classification and tumor stage (Figure S2) supports the relevance of our findings despite the small cohort size. Hence, UCHL1 contributes to GC pathogenesis.

CIP2A, a known oncoprotein overexpressed in multiple malignancies, modulates multiple oncogenic signaling pathways driving tumor growth and progression [21]. Specifically, CIP2A promotes cancer cell proliferation, survival, and migration by inhibiting PP2A-mediated dephosphorylation of key substrates, such as c-Myc [27,28]. Through these interactions, CIP2A plays a pivotal role in the regulation of tumor-promoting signaling networks.

This study uncovered a novel regulatory mechanism through which UCHL1 directly controls the protein stability of CIP2A via ubiquitination-dependent degradation. We demonstrated that UCHL1 physically interacts with CIP2A and removes its ubiquitin chains, thereby preventing its proteasomal degradation (Figure 3F). Importantly, mutant UCHL1 with dead catalytic activity (UCHL1 C90S) failed to mediate this ubiquitination, confirming that the enzymatic activity of UCHL1 is essential for CIP2A stabilization (Figure 3G).

Previous studies linked UCHL1 to the regulation of the cell cycle in tumor growth and progression [35]. Generally, UPS is essential for maintaining cell cycle homeostasis [6]. Among its components, UCHL1 functions as a key regulator and influences this process [36]. For example, pharmacological inhibition of UCHL1 with LDN-57444 induces the G1 phase cell cycle arrest in multiple myeloma [37]. Consistent with the observation, our study demonstrated that the UCHL1-CIP2A axis contributes to cell cycle regulation in GC. Both UCHL1 genetic knockdown and pharmacological inhibition with LDN-57444 significantly reduced cyclin B1 and cyclin D1 expression, resulting in the pronounced G1 phase cell cycle arrest (Figure 5A,C). Of note, the siRNA sequences used in this study are relatively short, and although random matches may be limited, potential off-target effects cannot be completely ruled out. Collectively, our findings support a mechanism through which UCHL1 promotes tumor progression by modulating the CIP2A-c-Myc-cyclin signaling cascade.

Taken together, we identified a novel cell progression mechanism of UCHL1 in GC, through which it regulates CIP2A stability through the UPS, particularly via its deubiquitinating enzyme activity. UCHL1 suppression, either through physiological downregulation or pharmacological inhibition, markedly reduced GC progression. Notably, this anti-cancer effect was mediated through CIP2A deubiquitination, highlighting UCHL1 as a potential therapeutic target for future clinical investigation in GC.

## 4. Materials and Methods

### 4.1. Reagents and Antibodies

The antibodies used in this study were obtained: CIP2A (sc-80659, Santa Cruz Biotechnology, CA, USA), β-actin (sc-47778, Santa Cruz Biotechnology), c-Myc (sc-40, Santa Cruz Biotechnology), MYC tag (sc-40, Santa Cruz Biotechnology), UCHL1 (13179, Cell Signaling Technology), cyclin D1 (2978, Cell Signaling Technology, Danvers, MA, USA), cyclin B1 (4138, Cell Signaling Technology), DYKDDDDK tag (2368, Cell Signaling Technology), and HA tag (MMS-101P, Covance, Princeton, NJ, USA). Anti-HA antibody–conjugated agarose beads (2095), cycloheximide (01810), and MG132 (474790) were purchased from Sigma Aldrich (St. Louis, MO, USA). Protein A/G agarose beads (20422) were obtained from Thermo Fisher Scientific (Waltham, MA, USA).

### 4.2. Patients and Gastric Tissue Samples

Written informed consent was obtained from all the patients with GC who participated in this study. The Institutional Review Board of Asan Medical Center and the University of Ulsan College of Medicine approved all experimental protocols (2016-1275). Human gastric tissues were obtained from the Asan Bio-Resource Center (2016-24(136)), which waived the need for informed consent due to its retrospective design.

### 4.3. Cell Culture

GC cell lines MKN1 and SNU484 were acquired from the Korean Cell Line Bank (KCLB, Seoul, Republic of Korea) and maintained with a Roswell Park Memorial Institute (RPMI) 1640 supplemented with 10% fetal bovine serum (FBS) and 1% penicillin/streptomycin (P/S). The 293T cells were obtained from the American Type Culture Collection (ATCC) and cultured in a Dulbecco’s Modified Eagle’s Medium (DMEM) supplemented with 10% FBS (Corning, 35-015-CV, NY, USA) and 1% P/S (Corning, 30-002-CI).

### 4.4. Immunohistochemistry (IHC) Staining

Section slides with human gastric tumor and normal tissues were dewaxed in xylene (10 min, two times) and rehydrated in ethanol of different gradients. Dewaxed slides were incubated with 1% bovine serum albumin (BSA) in phosphate-buffered saline (PBS)-T for at least 1 h at room temperature and incubated with anti-UCHL1 antibody (1:100) at 4 °C overnight, followed by 15 min incubation with 3% H_2_O_2_ solution to inactivate endogenous peroxidases. The section slides were treated with secondary antibody against the host for at least 1 h at room temperature and continuously exposed to ABC reagent (Vector Lab, PK-7200, Newark, CA, USA). Subsequently, the slides were stained with DAB (Agilent, K3468, Santa Clara, CA, USA) and hematoxylin (Sigma Aldrich, MHS32, St. Louis, MO, USA). The slides were rehydrated through ethanol and xylene to prepare them for mounting. Then, the mounting slides were analyzed using a light microscope.

### 4.5. Transfection of Plasmids or siRNAs

All the plasmids (HA-CIP2A, MYC-UCHL1 WT, MYC-UCHL1 C90A, and FLAG-UB) were cloned into CMV-HA, CMV-MYC, and CMV-FLAG vectors for transient transfection using Gateway technology (Invitrogen^TM^, 11791920, Carlsbad, CA, USA). HEK293T cells were transiently transfected using Lipofectamine 3000™ (Invitrogen, 15222475). Lipofectamine 3000 was mixed in OptiMEM (Thermo Fisher Scientific, 31985-070, Waltham, MA, USA), and plasmid DNAs were mixed with the P3000 in OptiMEM for 10 min. Then, each tube was mixed for 10 min. The transfected cells during 48 h were harvested and lysed for Western blot analysis.

All small interfering RNAs (siRNAs) for human UCHL1 were commercially synthesized by Genolution and Bioneer. MKN1 and SNU484 cells were transiently knocked down using Lipofectamine™ RNAiMAX (Invitrogen, 13778500). The siRNA was incubated with the transfection mixture for 15 min. The transfected cells during 48 h were harvested and lysed for Western blot analysis. The following specific sequences of UCHL1 were used: siUCHL1 #1 (sense, 5′GGACAAGAAGUUAGUCCUA3′), siUCHL1 #2 (sense, 5′GGCCAAUAAUCAAGACAAA-3′), and siUCHL1 #3 (sense, 5′-AAGUUAGUCCUAAAGUGUA-3′).

### 4.6. Western Blot Analysis

Patient-derived gastric cancer tissue samples were lysed in RIPA buffer containing 50 mM Tris (pH 7.5), 0.1% SDS, 1% Triton X-100, 150 mM NaCl, 0.5% sodium deoxycholate, 2 mM EDTA, and an EDTA-free protease inhibitor cocktail (Abcam, 270055, Cambridge, MA, USA). The lysates were centrifuged at maximum speed for 30 min, and total extracted protein levels were determined using a BCA assay kit (Pierce, 23225, Appleton, WI, USA). Subsequently, 40 μg of protein extracts were loaded onto an SDS-PAGE gel for Western blot analysis. Also, cells were lysed in 1% SDS lysis buffer composed of 40 mM Tris (pH 8.0), 150 mM NaCl, 1% SDS, and 1 mM EDTA. Total extracted protein levels were determined using a BCA assay kit, and appropriate amounts of protein were loaded onto SDS-PAGE gels for Western blot analysis.

### 4.7. Immunoprecipitation

For co-immunoprecipitation (Co-IP) experiments, cells were lysed in MCLB buffer containing 50 mM Tris (pH 7.4), 150 mM NaCl, 0.5% NP-40, and an EDTA-free protease inhibitor cocktail. The lysates were incubated with anti-HA antibody–conjugated agarose beads for at least 12 h at 4 °C. The beads were then washed five times with MCLB buffer and analyzed by Western blot. For endogenous immunoprecipitation (endo-IP), cells were lysed using the same method as in the Co-IP experiments. The supernatants were rotated with anti-CIP2A antibody for at least 12 h at 4 °C, and the resulting antibody–protein complexes were washed as described above before Western blot analysis.

### 4.8. Mass Spectrometry Analysis

For proteomic analysis, the CIP2A-HA plasmid was stably expressed in 293T cells. Cells were lysed and subjected to immunoprecipitation experiments, and any complexes formed were analyzed on a Thermo Orbitrap-XL mass spectrometer, as previously described [38,39,40].

### 4.9. Flow Cytometry for the Detection of Cell Cycle

To analyze cell cycle distribution, cells (1 × 10^6^ cells) were harvested and washed with PBS. The cells were then fixed with 70% ethanol 4 °C for at least 2 h. For DNA staining, propidium iodide (0.1 mg/mL PI and 0.1 mg/mL RNase in Auto DW) was added and stayed at room temperature for at least 20 min. The cell cycle phases were analyzed using FlowJo software (version 10.8.1, BD Biosciences, Ashland, OR, USA, accessed on 17 May 2025).

### 4.10. Cell Synchronization

G2/M phase arrest was stimulated by treating cells with 0.1 μg/mL nocodazole (Sigma Aldrich, M1404, St. Louis, MO, USA) for 16 h. To allow cell cycle re-entry, the cells were released into complete medium. Simultaneously, cells were harvested every 8 h and subjected to Western blot analysis.

### 4.11. Statistical Analysis

The results from at least three independent experiments are presented as the standard deviation (SD) and were analyzed statistically using GraphPad Prism 10. Statistical significance was determined using a two-sided Student’s *t*-test and defined as *p* < 0.05.

## Data Availability

The original contributions presented in this study are included in the article/Supplementary Material. Further inquiries can be directed to the corresponding author(s).

## Supplementary Materials

The following supporting information can be downloaded at https://www.mdpi.com/article/10.3390/ph18101468/s1: Figure S1: The expression of UCHL1 in gastric cancer tissues. Figure S2. Kaplan–Meier Analysis of Overall Survival in Gastric Cancer Patients Stratified by UCHL1 Expression Across Lauren Classification Types (A) Kaplan-Meier survival curves for high (red line) and low (blue line) UCHL1 expression groups in Stage I patients. (B) Kaplan-Meier survival curves for high (red line) and low (blue line) UCHL1 expression groups in Stage II patients. (C) Kaplan-Meier survival curves for high (red line) and low (black line) UCHL1 expression groups in Stage III patients. (D) Kaplan-Meier survival curves for high (red line) and low (black line) UCHL1 expression groups in Stage IV patients. (E) Kaplan-Meier survival curves for high (red line) and low (blue line) UCHL1 expression groups in Intestinal type. (F) Kaplan-Meier survival curves for high (red line) and low (blue line) UCHL1 expression groups in Diffuse type. Figure S3. The mRNA and Protein Expression level of UCHL1 in Gastric Cancer Cell Lines. Figure S3. The mRNA and Protein Expression level of UCHL1 in Gastric Cancer Cell Lines (A) The expression levels of UCHL1 mRNA (nTPM, bar graph) and protein (circle markers) were analyzed across multiple gastric cancer cell lines using the Human Protein Atlas. Table S1: Mass spectrometry Analysis on CIP2A-interacting proteins in 293T cells.

## Abbreviations

The following abbreviations are used in this manuscript:

GC	Gastric Cancer
UPS	Ubiquitin Proteasome System
DUB	Deubiquitinating Enzyme
CIP2A	Cancerous Inhibitor of Protein Phosphatase
PP2A	Protein Phosphatase 2A
BAP1	BRCA1-associated protein 1
E2F1	E2F transcription factor 1
UCHL1	Ubiquitin C-terminal Hydrolase L1
