# Supplementary material for: UCHL1 Promotes Gastric Cancer Progression by Regulating CIP2A Degradation
Source: Pharmaceuticals (Basel). 2025 Sep 29;18(10):1468. doi: 10.3390/ph18101468 (PMC12567482; doi:10.3390/ph18101468)
Supplement: Supplementary file 1 [file pharmaceuticals-18-01468-s001.zip › pharmaceuticals-3863805-supplementary.pptx]

## Slide 1
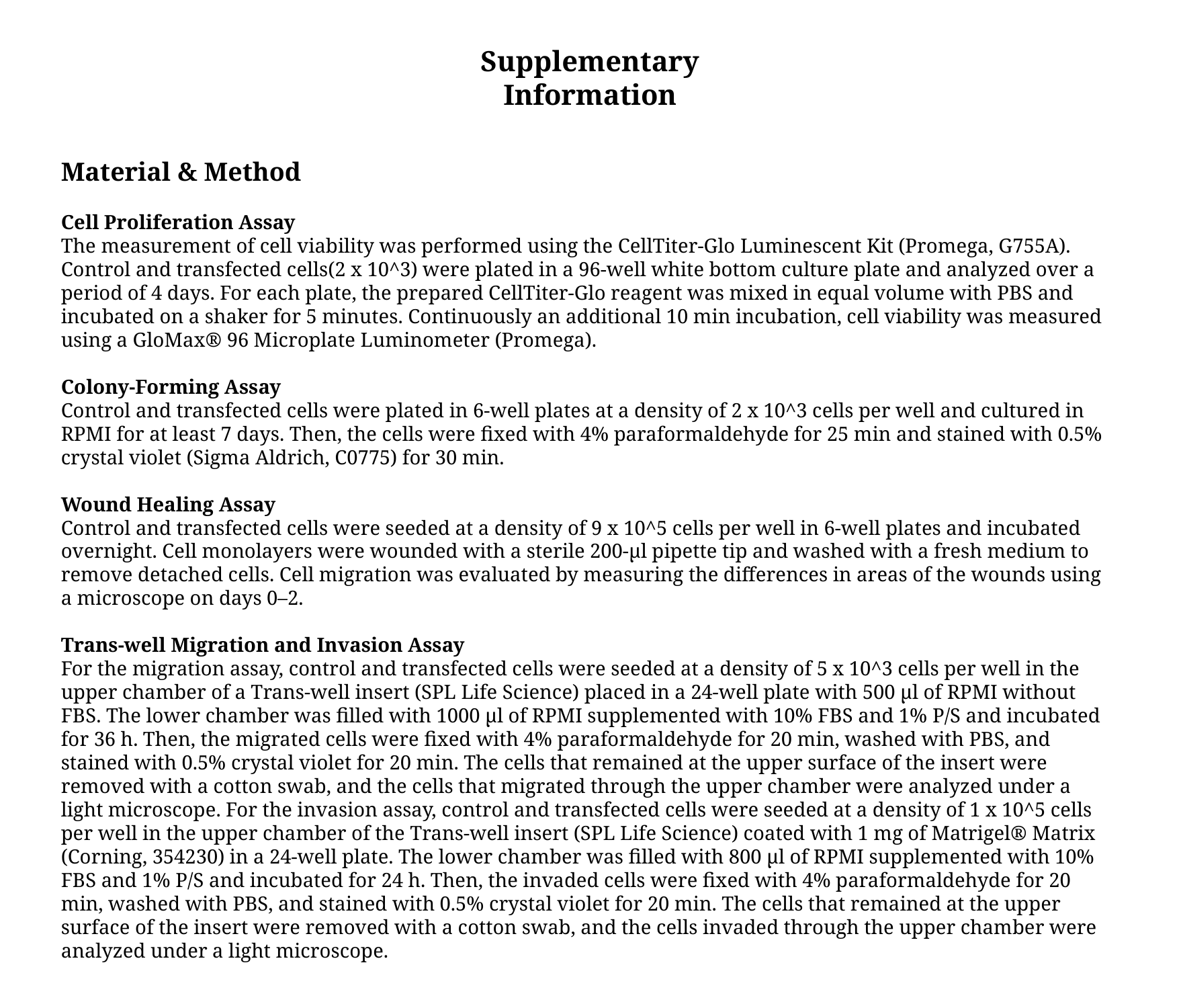

Supplementary Information
Material & Method
Cell Proliferation Assay
The measurement of cell viability was performed using the CellTiter-Glo Luminescent Kit (Promega, G755A). Control and transfected cells(2 x 10^3) were plated in a 96-well white bottom culture plate and analyzed over a period of 4 days. For each plate, the prepared CellTiter-Glo reagent was mixed in equal volume with PBS and incubated on a shaker for 5 minutes. Continuously an additional 10 min incubation, cell viability was measured using a GloMax® 96 Microplate Luminometer (Promega).
Colony-Forming Assay
Control and transfected cells were plated in 6-well plates at a density of 2 x 10^3 cells per well and cultured in RPMI for at least 7 days. Then, the cells were fixed with 4% paraformaldehyde for 25 min and stained with 0.5% crystal violet (Sigma Aldrich, C0775) for 30 min.
Wound Healing Assay
Control and transfected cells were seeded at a density of 9 x 10^5 cells per well in 6-well plates and incubated overnight. Cell monolayers were wounded with a sterile 200-μl pipette tip and washed with a fresh medium to remove detached cells. Cell migration was evaluated by measuring the differences in areas of the wounds using a microscope on days 0–2.
Trans-well Migration and Invasion Assay
For the migration assay, control and transfected cells were seeded at a density of 5 x 10^3 cells per well in the upper chamber of a Trans-well insert (SPL Life Science) placed in a 24-well plate with 500 μl of RPMI without FBS. The lower chamber was filled with 1000 μl of RPMI supplemented with 10% FBS and 1% P/S and incubated for 36 h. Then, the migrated cells were fixed with 4% paraformaldehyde for 20 min, washed with PBS, and stained with 0.5% crystal violet for 20 min. The cells that remained at the upper surface of the insert were removed with a cotton swab, and the cells that migrated through the upper chamber were analyzed under a light microscope. For the invasion assay, control and transfected cells were seeded at a density of 1 x 10^5 cells per well in the upper chamber of the Trans-well insert (SPL Life Science) coated with 1 mg of Matrigel® Matrix (Corning, 354230) in a 24-well plate. The lower chamber was filled with 800 μl of RPMI supplemented with 10% FBS and 1% P/S and incubated for 24 h. Then, the invaded cells were fixed with 4% paraformaldehyde for 20 min, washed with PBS, and stained with 0.5% crystal violet for 20 min. The cells that remained at the upper surface of the insert were removed with a cotton swab, and the cells invaded through the upper chamber were analyzed under a light microscope.

## Slide 2
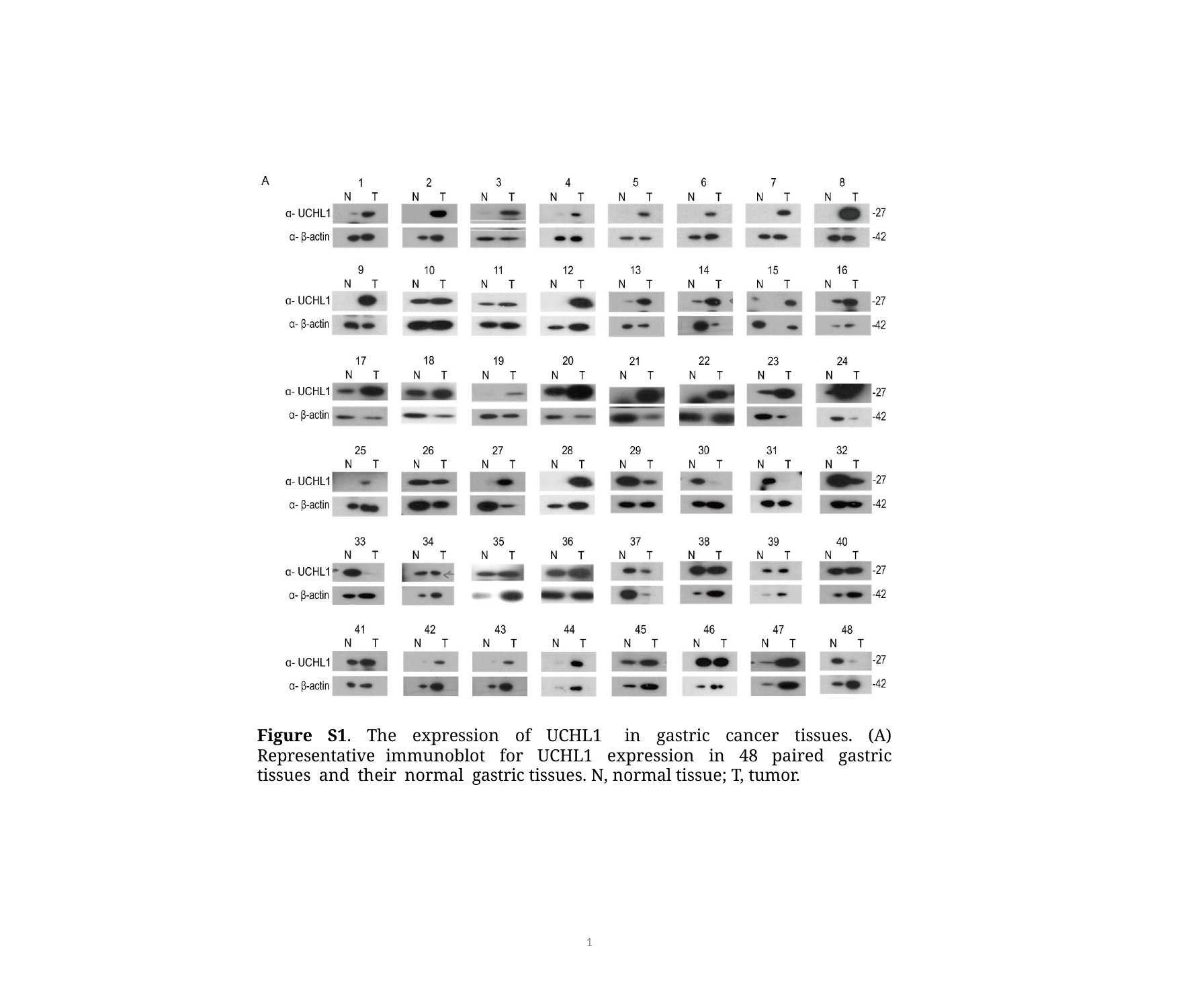

Figure S1. The expression of UCHL1 in gastric cancer tissues. (A) Representative immunoblot for UCHL1 expression in 48 paired gastric tissues and their normal gastric tissues. N, normal tissue; T, tumor.
1

## Slide 3
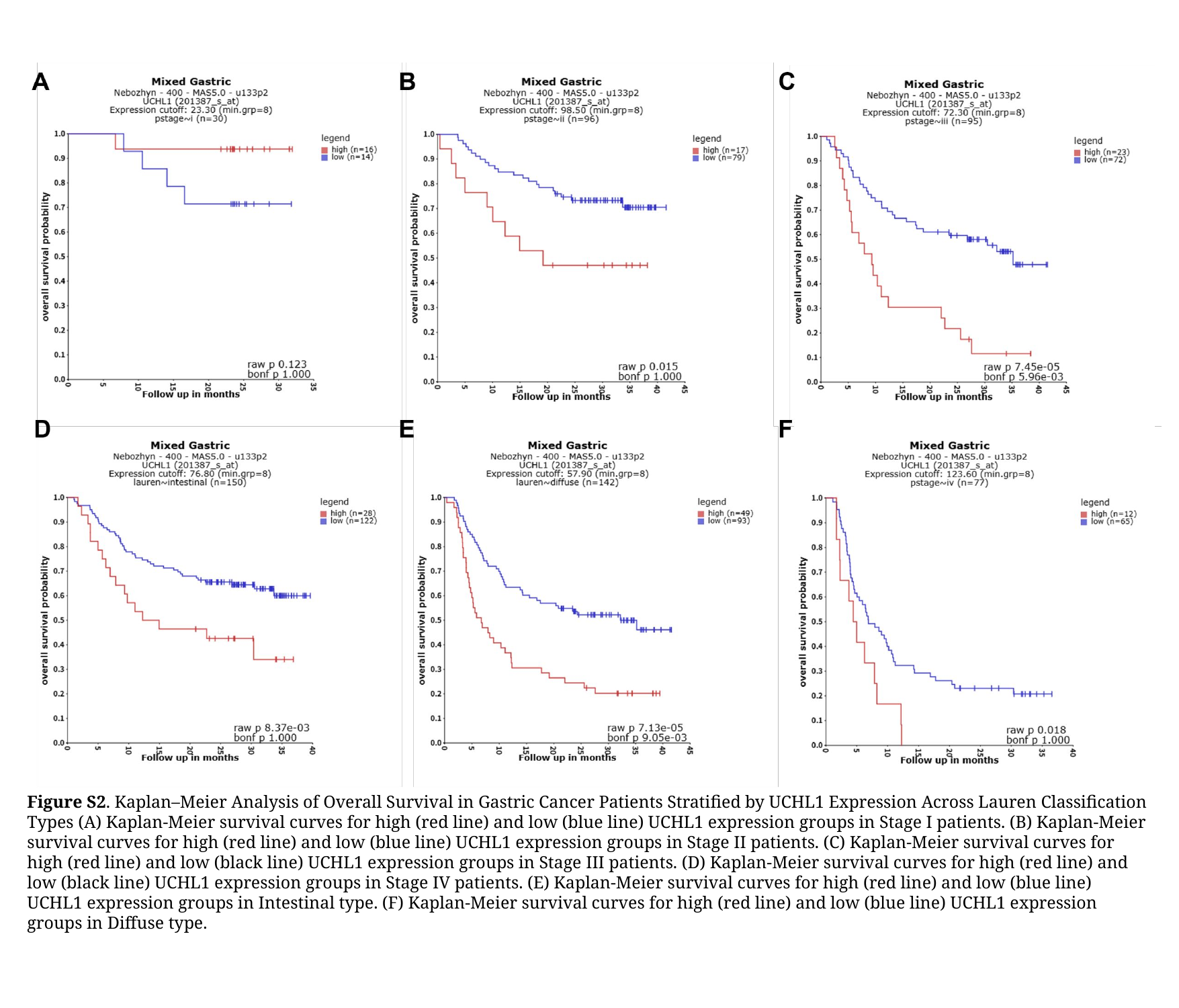

Figure S2. Kaplan–Meier Analysis of Overall Survival in Gastric Cancer Patients Stratified by UCHL1 Expression Across Lauren Classification Types (A) Kaplan-Meier survival curves for high (red line) and low (blue line) UCHL1 expression groups in Stage I patients. (B) Kaplan-Meier survival curves for high (red line) and low (blue line) UCHL1 expression groups in Stage II patients. (C) Kaplan-Meier survival curves for high (red line) and low (black line) UCHL1 expression groups in Stage III patients. (D) Kaplan-Meier survival curves for high (red line) and low (black line) UCHL1 expression groups in Stage IV patients. (E) Kaplan-Meier survival curves for high (red line) and low (blue line) UCHL1 expression groups in Intestinal type. (F) Kaplan-Meier survival curves for high (red line) and low (blue line) UCHL1 expression groups in Diffuse type.

## Slide 4
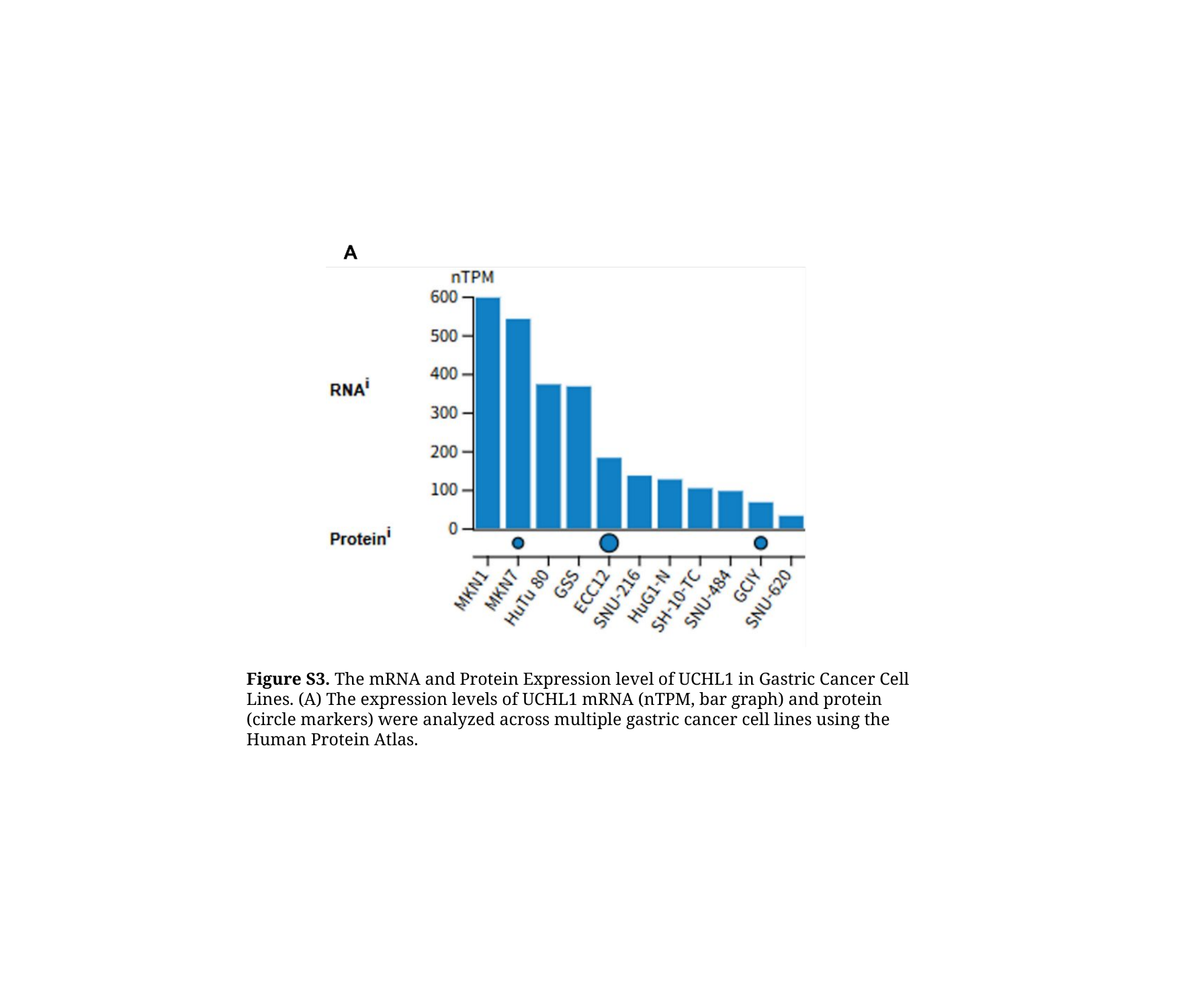

Figure S3. The mRNA and Protein Expression level of UCHL1 in Gastric Cancer Cell Lines. (A) The expression levels of UCHL1 mRNA (nTPM, bar graph) and protein (circle markers) were analyzed across multiple gastric cancer cell lines using the Human Protein Atlas.

## Slide 5
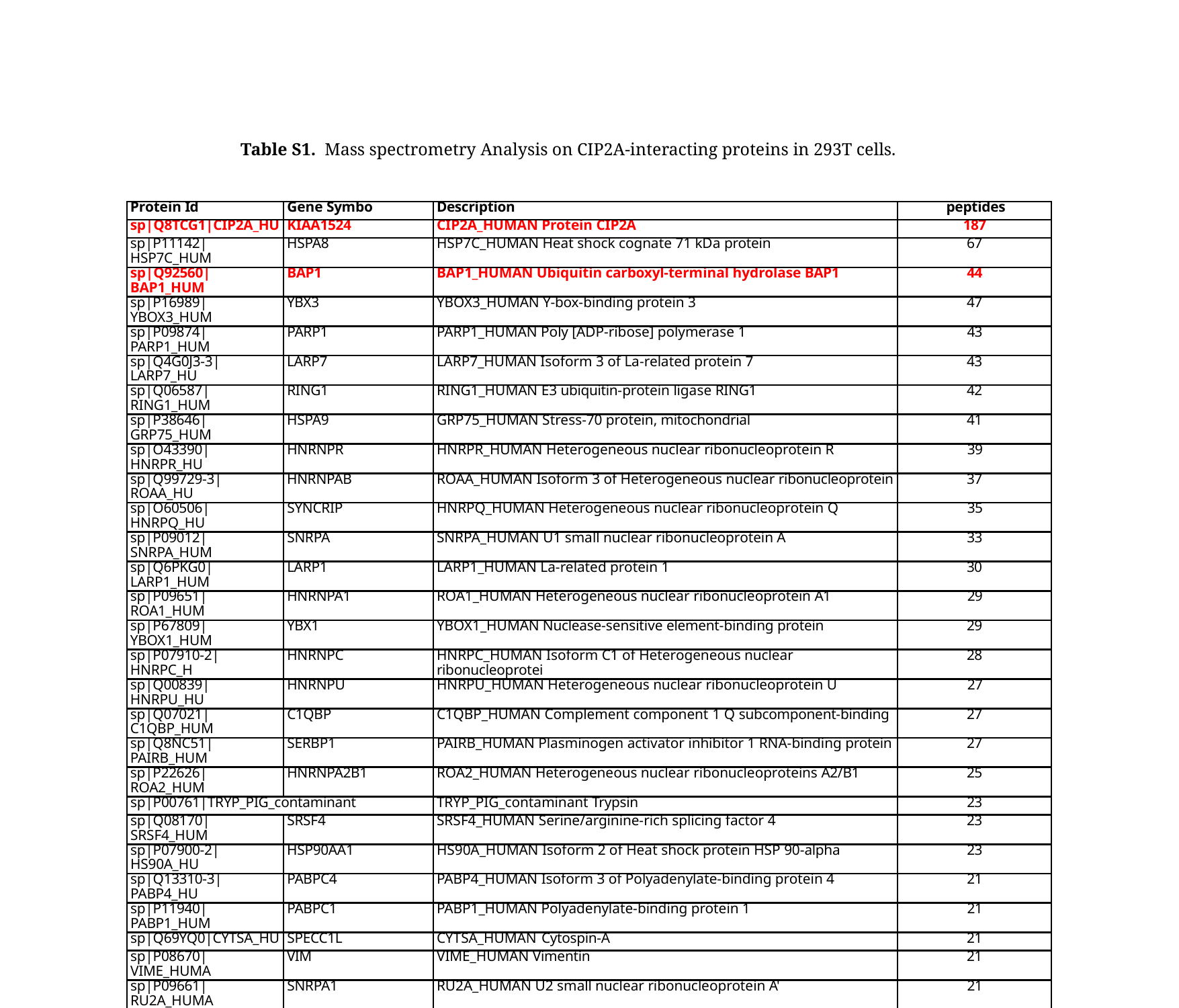

Table S1.  Mass spectrometry Analysis on CIP2A-interacting proteins in 293T cells.
| Protein Id | Gene Symbo | Description | peptides |
| --- | --- | --- | --- |
| sp|Q8TCG1|CIP2A\_HU | KIAA1524 | CIP2A\_HUMAN Protein CIP2A | 187 |
| sp|P11142|HSP7C\_HUM | HSPA8 | HSP7C\_HUMAN Heat shock cognate 71 kDa protein | 67 |
| sp|Q92560|BAP1\_HUM | BAP1 | BAP1\_HUMAN Ubiquitin carboxyl-terminal hydrolase BAP1 | 44 |
| sp|P16989|YBOX3\_HUM | YBX3 | YBOX3\_HUMAN Y-box-binding protein 3 | 47 |
| sp|P09874|PARP1\_HUM | PARP1 | PARP1\_HUMAN Poly [ADP-ribose] polymerase 1 | 43 |
| sp|Q4G0J3-3|LARP7\_HU | LARP7 | LARP7\_HUMAN Isoform 3 of La-related protein 7 | 43 |
| sp|Q06587|RING1\_HUM | RING1 | RING1\_HUMAN E3 ubiquitin-protein ligase RING1 | 42 |
| sp|P38646|GRP75\_HUM | HSPA9 | GRP75\_HUMAN Stress-70 protein, mitochondrial | 41 |
| sp|O43390|HNRPR\_HU | HNRNPR | HNRPR\_HUMAN Heterogeneous nuclear ribonucleoprotein R | 39 |
| sp|Q99729-3|ROAA\_HU | HNRNPAB | ROAA\_HUMAN Isoform 3 of Heterogeneous nuclear ribonucleoprotein | 37 |
| sp|O60506|HNRPQ\_HU | SYNCRIP | HNRPQ\_HUMAN Heterogeneous nuclear ribonucleoprotein Q | 35 |
| sp|P09012|SNRPA\_HUM | SNRPA | SNRPA\_HUMAN U1 small nuclear ribonucleoprotein A | 33 |
| sp|Q6PKG0|LARP1\_HUM | LARP1 | LARP1\_HUMAN La-related protein 1 | 30 |
| sp|P09651|ROA1\_HUM | HNRNPA1 | ROA1\_HUMAN Heterogeneous nuclear ribonucleoprotein A1 | 29 |
| sp|P67809|YBOX1\_HUM | YBX1 | YBOX1\_HUMAN Nuclease-sensitive element-binding protein | 29 |
| sp|P07910-2|HNRPC\_H | HNRNPC | HNRPC\_HUMAN Isoform C1 of Heterogeneous nuclear ribonucleoprotei | 28 |
| sp|Q00839|HNRPU\_HU | HNRNPU | HNRPU\_HUMAN Heterogeneous nuclear ribonucleoprotein U | 27 |
| sp|Q07021|C1QBP\_HUM | C1QBP | C1QBP\_HUMAN Complement component 1 Q subcomponent-binding | 27 |
| sp|Q8NC51|PAIRB\_HUM | SERBP1 | PAIRB\_HUMAN Plasminogen activator inhibitor 1 RNA-binding protein | 27 |
| sp|P22626|ROA2\_HUM | HNRNPA2B1 | ROA2\_HUMAN Heterogeneous nuclear ribonucleoproteins A2/B1 | 25 |
| sp|P00761|TRYP\_PIG\_contaminant | | TRYP\_PIG\_contaminant Trypsin | 23 |
| sp|Q08170|SRSF4\_HUM | SRSF4 | SRSF4\_HUMAN Serine/arginine-rich splicing factor 4 | 23 |
| sp|P07900-2|HS90A\_HU | HSP90AA1 | HS90A\_HUMAN Isoform 2 of Heat shock protein HSP 90-alpha | 23 |
| sp|Q13310-3|PABP4\_HU | PABPC4 | PABP4\_HUMAN Isoform 3 of Polyadenylate-binding protein 4 | 21 |
| sp|P11940|PABP1\_HUM | PABPC1 | PABP1\_HUMAN Polyadenylate-binding protein 1 | 21 |
| sp|Q69YQ0|CYTSA\_HU | SPECC1L | CYTSA\_HUMAN Cytospin-A | 21 |
| sp|P08670|VIME\_HUMA | VIM | VIME\_HUMAN Vimentin | 21 |
| sp|P09661|RU2A\_HUMA | SNRPA1 | RU2A\_HUMAN U2 small nuclear ribonucleoprotein A' | 21 |
| sp|Q7KZF4|SND1\_HUM | SND1 | SND1\_HUMAN Staphylococcal nuclease domain-containing protein 1 | 20 |
| sp|P61978-2|HNRPK\_H | HNRNPK | HNRPK\_HUMAN Isoform 2 of Heterogeneous nuclear ribonucleoprotein | 19 |
| sp|Q9BRJ6|CG050\_HUM | C7orf50 | CG050\_HUMAN Uncharacterized protein C7orf50 | 19 |
| sp|Q07955|SRSF1\_HUM | SRSF1 | SRSF1\_HUMAN Serine/arginine-rich splicing factor 1 | 19 |
| sp|Q08211|DHX9\_HUM | DHX9 | DHX9\_HUMAN ATP-dependent RNA helicase A | 18 |
| sp|P62258|1433E\_HUM | YWHAE | 1433E\_HUMAN 14-3-3 protein epsilon | 18 |

## Slide 6
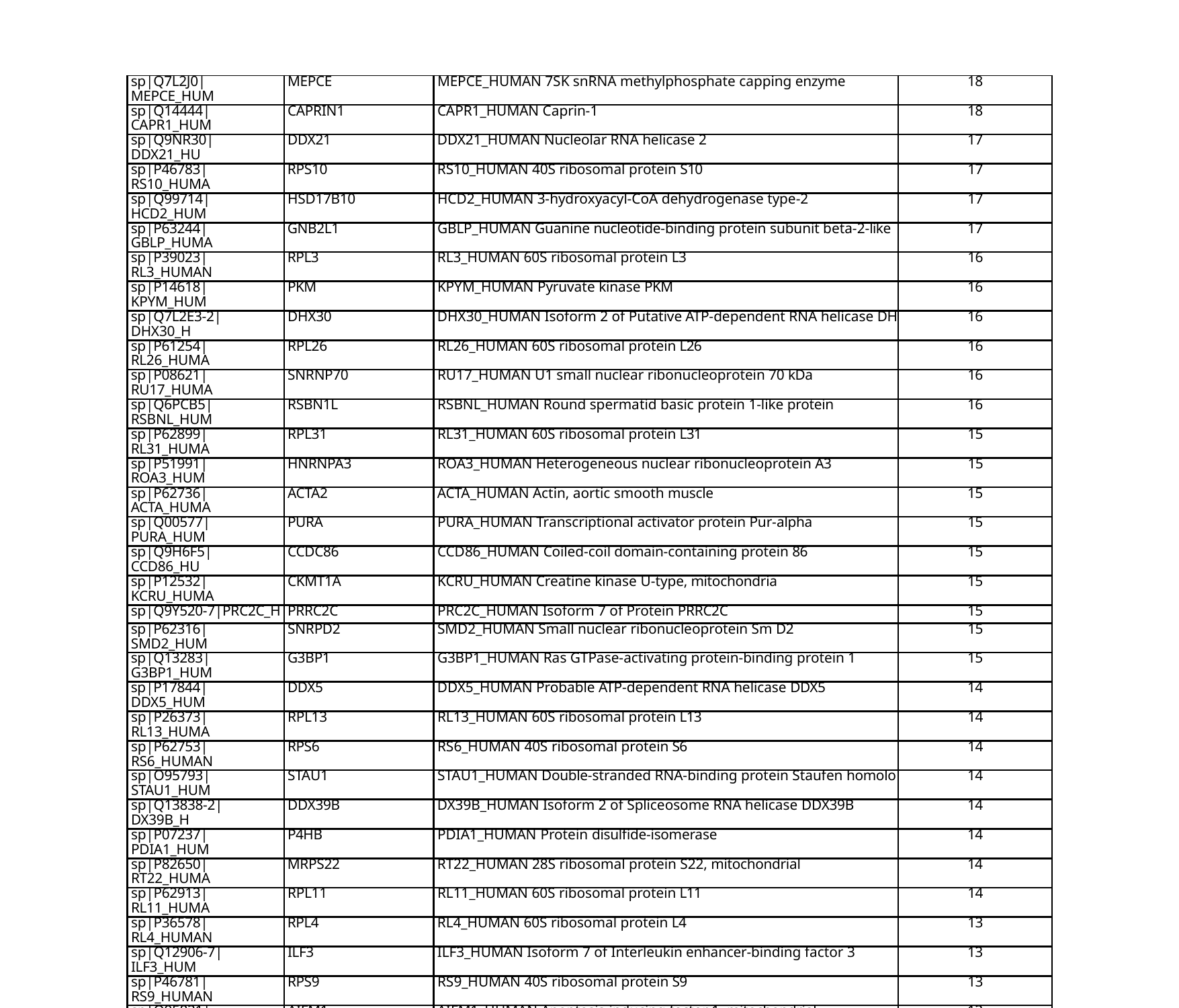

| sp|Q7L2J0|MEPCE\_HUM | MEPCE | MEPCE\_HUMAN 7SK snRNA methylphosphate capping enzyme | 18 |
| --- | --- | --- | --- |
| sp|Q14444|CAPR1\_HUM | CAPRIN1 | CAPR1\_HUMAN Caprin-1 | 18 |
| sp|Q9NR30|DDX21\_HU | DDX21 | DDX21\_HUMAN Nucleolar RNA helicase 2 | 17 |
| sp|P46783|RS10\_HUMA | RPS10 | RS10\_HUMAN 40S ribosomal protein S10 | 17 |
| sp|Q99714|HCD2\_HUM | HSD17B10 | HCD2\_HUMAN 3-hydroxyacyl-CoA dehydrogenase type-2 | 17 |
| sp|P63244|GBLP\_HUMA | GNB2L1 | GBLP\_HUMAN Guanine nucleotide-binding protein subunit beta-2-like | 17 |
| sp|P39023|RL3\_HUMAN | RPL3 | RL3\_HUMAN 60S ribosomal protein L3 | 16 |
| sp|P14618|KPYM\_HUM | PKM | KPYM\_HUMAN Pyruvate kinase PKM | 16 |
| sp|Q7L2E3-2|DHX30\_H | DHX30 | DHX30\_HUMAN Isoform 2 of Putative ATP-dependent RNA helicase DH | 16 |
| sp|P61254|RL26\_HUMA | RPL26 | RL26\_HUMAN 60S ribosomal protein L26 | 16 |
| sp|P08621|RU17\_HUMA | SNRNP70 | RU17\_HUMAN U1 small nuclear ribonucleoprotein 70 kDa | 16 |
| sp|Q6PCB5|RSBNL\_HUM | RSBN1L | RSBNL\_HUMAN Round spermatid basic protein 1-like protein | 16 |
| sp|P62899|RL31\_HUMA | RPL31 | RL31\_HUMAN 60S ribosomal protein L31 | 15 |
| sp|P51991|ROA3\_HUM | HNRNPA3 | ROA3\_HUMAN Heterogeneous nuclear ribonucleoprotein A3 | 15 |
| sp|P62736|ACTA\_HUMA | ACTA2 | ACTA\_HUMAN Actin, aortic smooth muscle | 15 |
| sp|Q00577|PURA\_HUM | PURA | PURA\_HUMAN Transcriptional activator protein Pur-alpha | 15 |
| sp|Q9H6F5|CCD86\_HU | CCDC86 | CCD86\_HUMAN Coiled-coil domain-containing protein 86 | 15 |
| sp|P12532|KCRU\_HUMA | CKMT1A | KCRU\_HUMAN Creatine kinase U-type, mitochondria | 15 |
| sp|Q9Y520-7|PRC2C\_H | PRRC2C | PRC2C\_HUMAN Isoform 7 of Protein PRRC2C | 15 |
| sp|P62316|SMD2\_HUM | SNRPD2 | SMD2\_HUMAN Small nuclear ribonucleoprotein Sm D2 | 15 |
| sp|Q13283|G3BP1\_HUM | G3BP1 | G3BP1\_HUMAN Ras GTPase-activating protein-binding protein 1 | 15 |
| sp|P17844|DDX5\_HUM | DDX5 | DDX5\_HUMAN Probable ATP-dependent RNA helicase DDX5 | 14 |
| sp|P26373|RL13\_HUMA | RPL13 | RL13\_HUMAN 60S ribosomal protein L13 | 14 |
| sp|P62753|RS6\_HUMAN | RPS6 | RS6\_HUMAN 40S ribosomal protein S6 | 14 |
| sp|O95793|STAU1\_HUM | STAU1 | STAU1\_HUMAN Double-stranded RNA-binding protein Staufen homolo | 14 |
| sp|Q13838-2|DX39B\_H | DDX39B | DX39B\_HUMAN Isoform 2 of Spliceosome RNA helicase DDX39B | 14 |
| sp|P07237|PDIA1\_HUM | P4HB | PDIA1\_HUMAN Protein disulfide-isomerase | 14 |
| sp|P82650|RT22\_HUMA | MRPS22 | RT22\_HUMAN 28S ribosomal protein S22, mitochondrial | 14 |
| sp|P62913|RL11\_HUMA | RPL11 | RL11\_HUMAN 60S ribosomal protein L11 | 14 |
| sp|P36578|RL4\_HUMAN | RPL4 | RL4\_HUMAN 60S ribosomal protein L4 | 13 |
| sp|Q12906-7|ILF3\_HUM | ILF3 | ILF3\_HUMAN Isoform 7 of Interleukin enhancer-binding factor 3 | 13 |
| sp|P46781|RS9\_HUMAN | RPS9 | RS9\_HUMAN 40S ribosomal protein S9 | 13 |
| sp|O95831|AIFM1\_HUM | AIFM1 | AIFM1\_HUMAN Apoptosis-inducing factor 1, mitochondrial | 13 |
| sp|P16402|H13\_HUMAN | HIST1H1D | H13\_HUMAN Histone H1.3 | 13 |
| sp|Q14103-4|HNRPD\_H | HNRNPD | HNRPD\_HUMAN Isoform 4 of Heterogeneous nuclear ribonucleoprotein | 13 |

## Slide 7
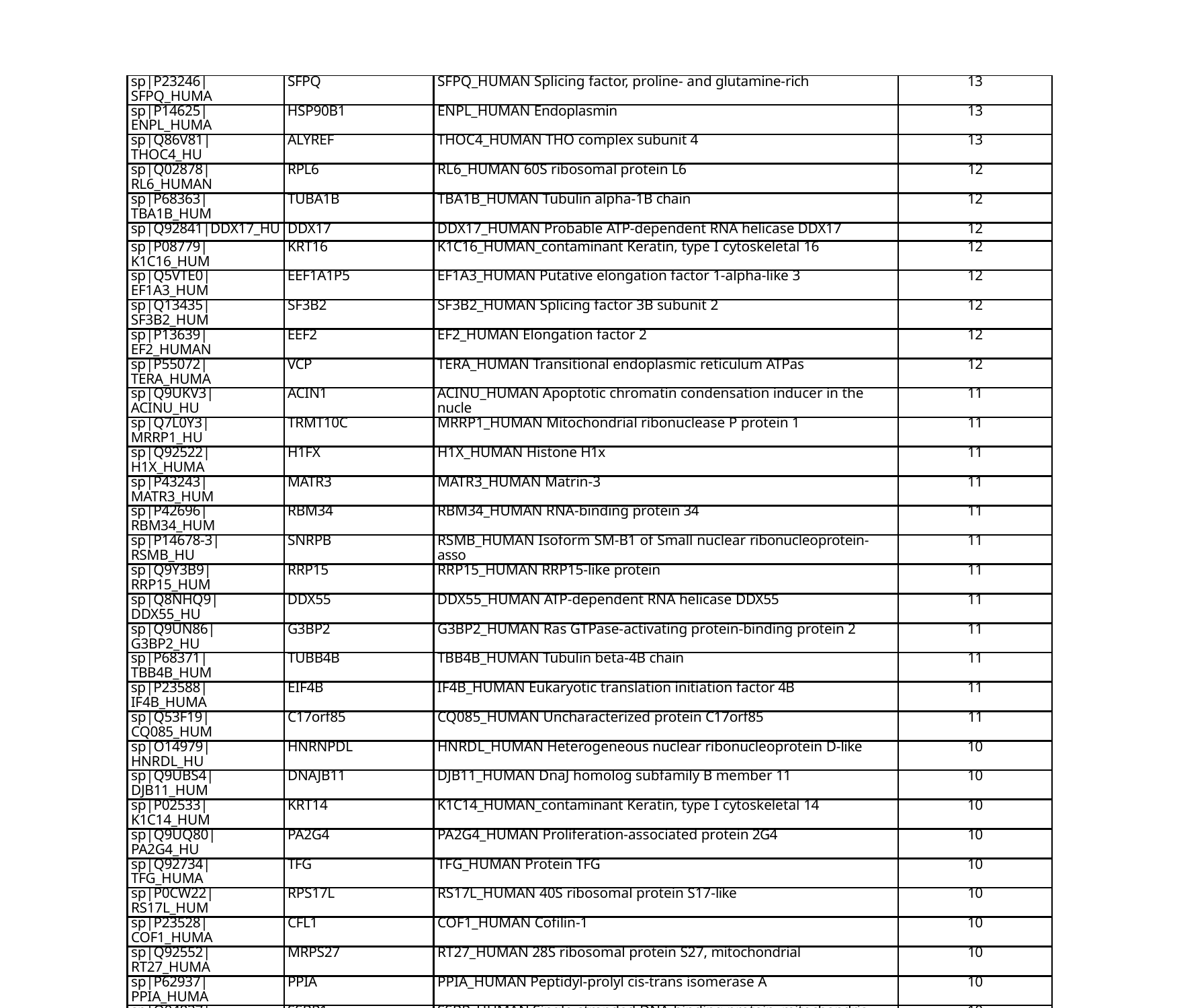

| sp|P23246|SFPQ\_HUMA | SFPQ | SFPQ\_HUMAN Splicing factor, proline- and glutamine-rich | 13 |
| --- | --- | --- | --- |
| sp|P14625|ENPL\_HUMA | HSP90B1 | ENPL\_HUMAN Endoplasmin | 13 |
| sp|Q86V81|THOC4\_HU | ALYREF | THOC4\_HUMAN THO complex subunit 4 | 13 |
| sp|Q02878|RL6\_HUMAN | RPL6 | RL6\_HUMAN 60S ribosomal protein L6 | 12 |
| sp|P68363|TBA1B\_HUM | TUBA1B | TBA1B\_HUMAN Tubulin alpha-1B chain | 12 |
| sp|Q92841|DDX17\_HU | DDX17 | DDX17\_HUMAN Probable ATP-dependent RNA helicase DDX17 | 12 |
| sp|P08779|K1C16\_HUM | KRT16 | K1C16\_HUMAN\_contaminant Keratin, type I cytoskeletal 16 | 12 |
| sp|Q5VTE0|EF1A3\_HUM | EEF1A1P5 | EF1A3\_HUMAN Putative elongation factor 1-alpha-like 3 | 12 |
| sp|Q13435|SF3B2\_HUM | SF3B2 | SF3B2\_HUMAN Splicing factor 3B subunit 2 | 12 |
| sp|P13639|EF2\_HUMAN | EEF2 | EF2\_HUMAN Elongation factor 2 | 12 |
| sp|P55072|TERA\_HUMA | VCP | TERA\_HUMAN Transitional endoplasmic reticulum ATPas | 12 |
| sp|Q9UKV3|ACINU\_HU | ACIN1 | ACINU\_HUMAN Apoptotic chromatin condensation inducer in the nucle | 11 |
| sp|Q7L0Y3|MRRP1\_HU | TRMT10C | MRRP1\_HUMAN Mitochondrial ribonuclease P protein 1 | 11 |
| sp|Q92522|H1X\_HUMA | H1FX | H1X\_HUMAN Histone H1x | 11 |
| sp|P43243|MATR3\_HUM | MATR3 | MATR3\_HUMAN Matrin-3 | 11 |
| sp|P42696|RBM34\_HUM | RBM34 | RBM34\_HUMAN RNA-binding protein 34 | 11 |
| sp|P14678-3|RSMB\_HU | SNRPB | RSMB\_HUMAN Isoform SM-B1 of Small nuclear ribonucleoprotein-asso | 11 |
| sp|Q9Y3B9|RRP15\_HUM | RRP15 | RRP15\_HUMAN RRP15-like protein | 11 |
| sp|Q8NHQ9|DDX55\_HU | DDX55 | DDX55\_HUMAN ATP-dependent RNA helicase DDX55 | 11 |
| sp|Q9UN86|G3BP2\_HU | G3BP2 | G3BP2\_HUMAN Ras GTPase-activating protein-binding protein 2 | 11 |
| sp|P68371|TBB4B\_HUM | TUBB4B | TBB4B\_HUMAN Tubulin beta-4B chain | 11 |
| sp|P23588|IF4B\_HUMA | EIF4B | IF4B\_HUMAN Eukaryotic translation initiation factor 4B | 11 |
| sp|Q53F19|CQ085\_HUM | C17orf85 | CQ085\_HUMAN Uncharacterized protein C17orf85 | 11 |
| sp|O14979|HNRDL\_HU | HNRNPDL | HNRDL\_HUMAN Heterogeneous nuclear ribonucleoprotein D-like | 10 |
| sp|Q9UBS4|DJB11\_HUM | DNAJB11 | DJB11\_HUMAN DnaJ homolog subfamily B member 11 | 10 |
| sp|P02533|K1C14\_HUM | KRT14 | K1C14\_HUMAN\_contaminant Keratin, type I cytoskeletal 14 | 10 |
| sp|Q9UQ80|PA2G4\_HU | PA2G4 | PA2G4\_HUMAN Proliferation-associated protein 2G4 | 10 |
| sp|Q92734|TFG\_HUMA | TFG | TFG\_HUMAN Protein TFG | 10 |
| sp|P0CW22|RS17L\_HUM | RPS17L | RS17L\_HUMAN 40S ribosomal protein S17-like | 10 |
| sp|P23528|COF1\_HUMA | CFL1 | COF1\_HUMAN Cofilin-1 | 10 |
| sp|Q92552|RT27\_HUMA | MRPS27 | RT27\_HUMAN 28S ribosomal protein S27, mitochondrial | 10 |
| sp|P62937|PPIA\_HUMA | PPIA | PPIA\_HUMAN Peptidyl-prolyl cis-trans isomerase A | 10 |
| sp|Q04837|SSBP\_HUMA | SSBP1 | SSBP\_HUMAN Single-stranded DNA-binding protein, mitochondria | 10 |
| sp|Q13601|KRR1\_HUMA | KRR1 | KRR1\_HUMAN KRR1 small subunit processome component homolog | 10 |
| sp|Q15233|NONO\_HUM | NONO | NONO\_HUMAN Non-POU domain-containing octamer-binding protein | 10 |

## Slide 8
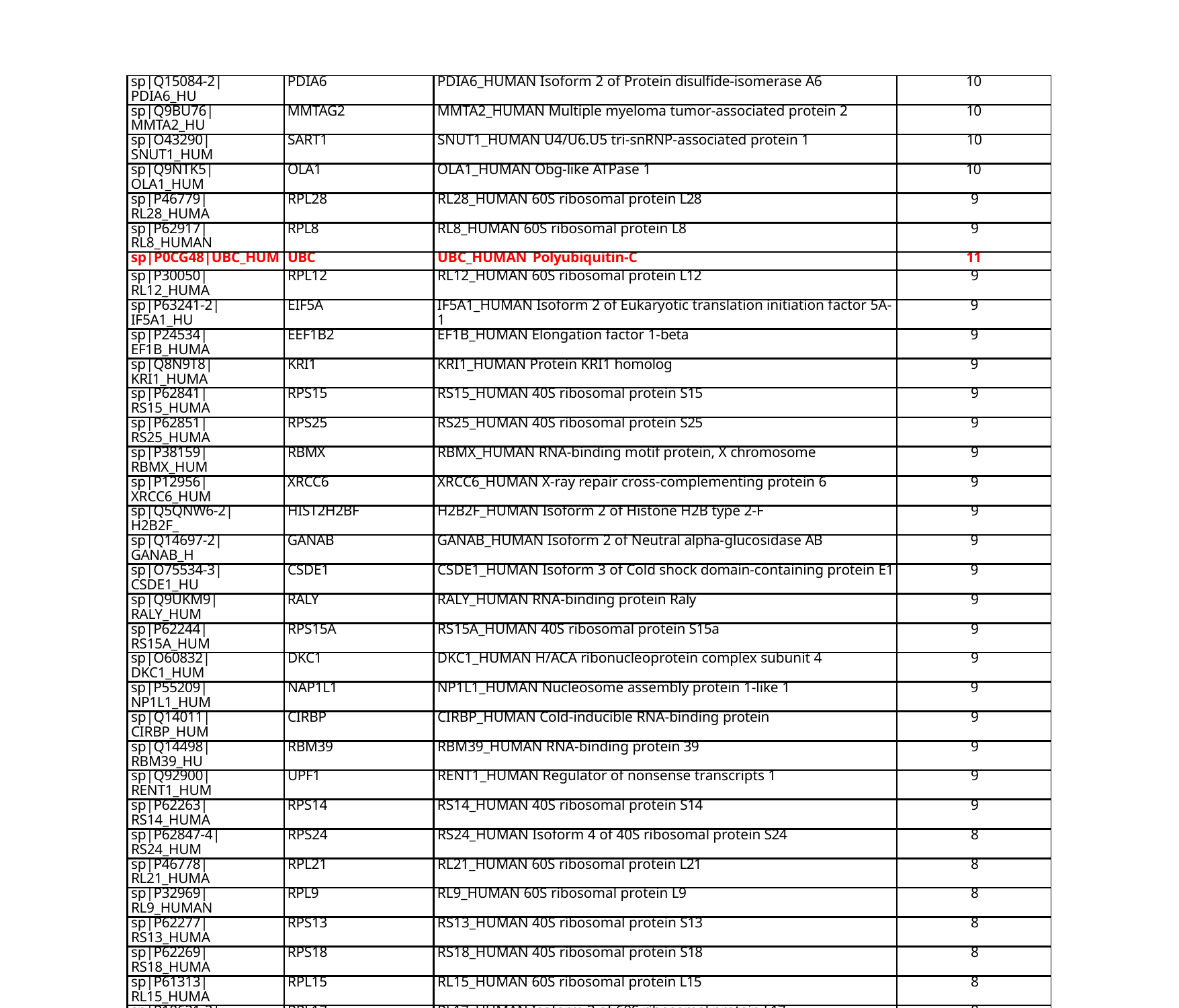

| sp|Q15084-2|PDIA6\_HU | PDIA6 | PDIA6\_HUMAN Isoform 2 of Protein disulfide-isomerase A6 | 10 |
| --- | --- | --- | --- |
| sp|Q9BU76|MMTA2\_HU | MMTAG2 | MMTA2\_HUMAN Multiple myeloma tumor-associated protein 2 | 10 |
| sp|O43290|SNUT1\_HUM | SART1 | SNUT1\_HUMAN U4/U6.U5 tri-snRNP-associated protein 1 | 10 |
| sp|Q9NTK5|OLA1\_HUM | OLA1 | OLA1\_HUMAN Obg-like ATPase 1 | 10 |
| sp|P46779|RL28\_HUMA | RPL28 | RL28\_HUMAN 60S ribosomal protein L28 | 9 |
| sp|P62917|RL8\_HUMAN | RPL8 | RL8\_HUMAN 60S ribosomal protein L8 | 9 |
| sp|P0CG48|UBC\_HUM | UBC | UBC\_HUMAN Polyubiquitin-C | 11 |
| sp|P30050|RL12\_HUMA | RPL12 | RL12\_HUMAN 60S ribosomal protein L12 | 9 |
| sp|P63241-2|IF5A1\_HU | EIF5A | IF5A1\_HUMAN Isoform 2 of Eukaryotic translation initiation factor 5A-1 | 9 |
| sp|P24534|EF1B\_HUMA | EEF1B2 | EF1B\_HUMAN Elongation factor 1-beta | 9 |
| sp|Q8N9T8|KRI1\_HUMA | KRI1 | KRI1\_HUMAN Protein KRI1 homolog | 9 |
| sp|P62841|RS15\_HUMA | RPS15 | RS15\_HUMAN 40S ribosomal protein S15 | 9 |
| sp|P62851|RS25\_HUMA | RPS25 | RS25\_HUMAN 40S ribosomal protein S25 | 9 |
| sp|P38159|RBMX\_HUM | RBMX | RBMX\_HUMAN RNA-binding motif protein, X chromosome | 9 |
| sp|P12956|XRCC6\_HUM | XRCC6 | XRCC6\_HUMAN X-ray repair cross-complementing protein 6 | 9 |
| sp|Q5QNW6-2|H2B2F\_ | HIST2H2BF | H2B2F\_HUMAN Isoform 2 of Histone H2B type 2-F | 9 |
| sp|Q14697-2|GANAB\_H | GANAB | GANAB\_HUMAN Isoform 2 of Neutral alpha-glucosidase AB | 9 |
| sp|O75534-3|CSDE1\_HU | CSDE1 | CSDE1\_HUMAN Isoform 3 of Cold shock domain-containing protein E1 | 9 |
| sp|Q9UKM9|RALY\_HUM | RALY | RALY\_HUMAN RNA-binding protein Raly | 9 |
| sp|P62244|RS15A\_HUM | RPS15A | RS15A\_HUMAN 40S ribosomal protein S15a | 9 |
| sp|O60832|DKC1\_HUM | DKC1 | DKC1\_HUMAN H/ACA ribonucleoprotein complex subunit 4 | 9 |
| sp|P55209|NP1L1\_HUM | NAP1L1 | NP1L1\_HUMAN Nucleosome assembly protein 1-like 1 | 9 |
| sp|Q14011|CIRBP\_HUM | CIRBP | CIRBP\_HUMAN Cold-inducible RNA-binding protein | 9 |
| sp|Q14498|RBM39\_HU | RBM39 | RBM39\_HUMAN RNA-binding protein 39 | 9 |
| sp|Q92900|RENT1\_HUM | UPF1 | RENT1\_HUMAN Regulator of nonsense transcripts 1 | 9 |
| sp|P62263|RS14\_HUMA | RPS14 | RS14\_HUMAN 40S ribosomal protein S14 | 9 |
| sp|P62847-4|RS24\_HUM | RPS24 | RS24\_HUMAN Isoform 4 of 40S ribosomal protein S24 | 8 |
| sp|P46778|RL21\_HUMA | RPL21 | RL21\_HUMAN 60S ribosomal protein L21 | 8 |
| sp|P32969|RL9\_HUMAN | RPL9 | RL9\_HUMAN 60S ribosomal protein L9 | 8 |
| sp|P62277|RS13\_HUMA | RPS13 | RS13\_HUMAN 40S ribosomal protein S13 | 8 |
| sp|P62269|RS18\_HUMA | RPS18 | RS18\_HUMAN 40S ribosomal protein S18 | 8 |
| sp|P61313|RL15\_HUMA | RPL15 | RL15\_HUMAN 60S ribosomal protein L15 | 8 |
| sp|P18621-3|RL17\_HUM | RPL17 | RL17\_HUMAN Isoform 3 of 60S ribosomal protein L17 | 8 |
| sp|P62424|RL7A\_HUMA | RPL7A | RL7A\_HUMAN 60S ribosomal protein L7a | 8 |
| sp|P62280|RS11\_HUMA | RPS11 | RS11\_HUMAN 40S ribosomal protein S11 | 8 |

## Slide 9
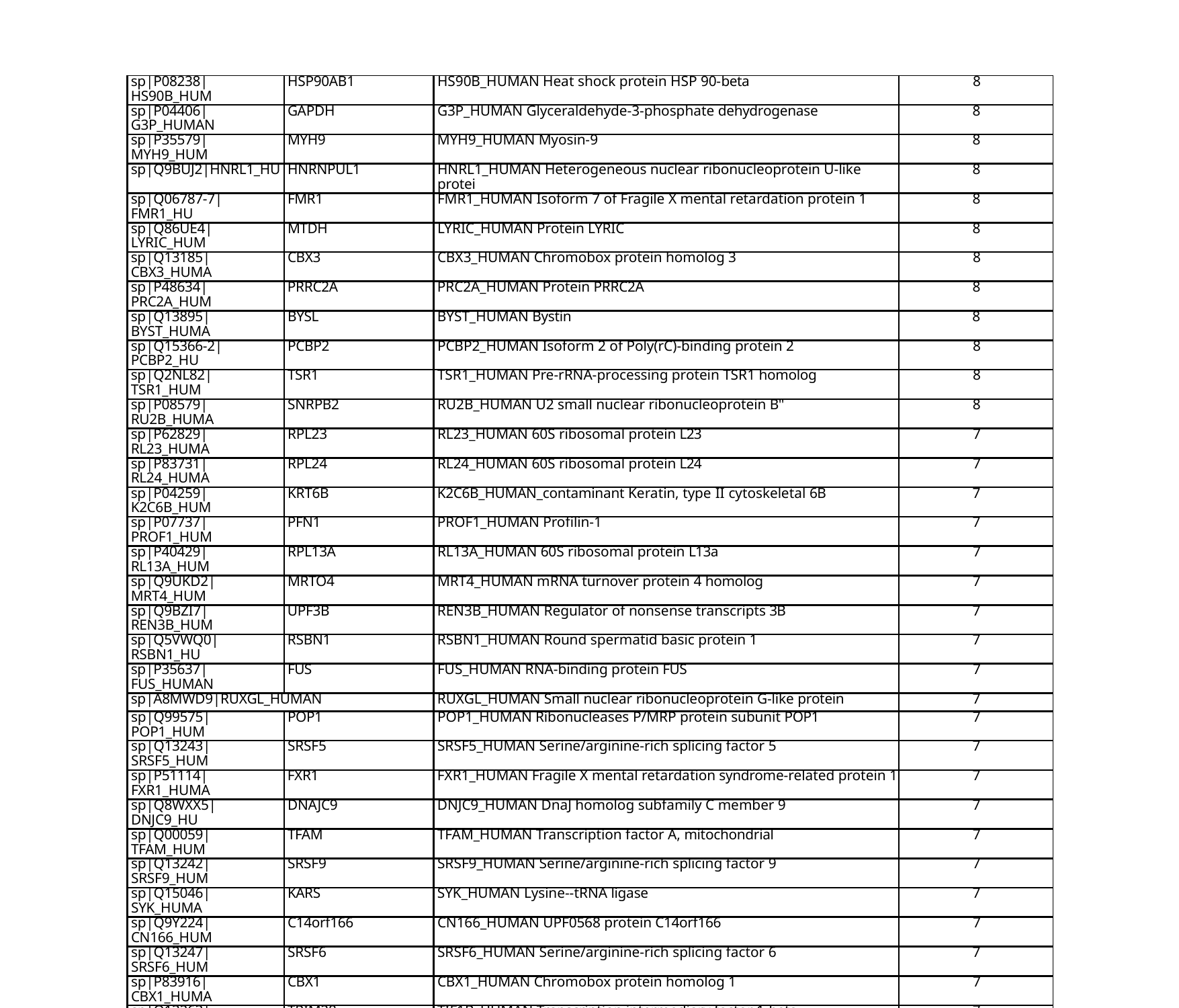

| sp|P08238|HS90B\_HUM | HSP90AB1 | HS90B\_HUMAN Heat shock protein HSP 90-beta | 8 |
| --- | --- | --- | --- |
| sp|P04406|G3P\_HUMAN | GAPDH | G3P\_HUMAN Glyceraldehyde-3-phosphate dehydrogenase | 8 |
| sp|P35579|MYH9\_HUM | MYH9 | MYH9\_HUMAN Myosin-9 | 8 |
| sp|Q9BUJ2|HNRL1\_HU | HNRNPUL1 | HNRL1\_HUMAN Heterogeneous nuclear ribonucleoprotein U-like protei | 8 |
| sp|Q06787-7|FMR1\_HU | FMR1 | FMR1\_HUMAN Isoform 7 of Fragile X mental retardation protein 1 | 8 |
| sp|Q86UE4|LYRIC\_HUM | MTDH | LYRIC\_HUMAN Protein LYRIC | 8 |
| sp|Q13185|CBX3\_HUMA | CBX3 | CBX3\_HUMAN Chromobox protein homolog 3 | 8 |
| sp|P48634|PRC2A\_HUM | PRRC2A | PRC2A\_HUMAN Protein PRRC2A | 8 |
| sp|Q13895|BYST\_HUMA | BYSL | BYST\_HUMAN Bystin | 8 |
| sp|Q15366-2|PCBP2\_HU | PCBP2 | PCBP2\_HUMAN Isoform 2 of Poly(rC)-binding protein 2 | 8 |
| sp|Q2NL82|TSR1\_HUM | TSR1 | TSR1\_HUMAN Pre-rRNA-processing protein TSR1 homolog | 8 |
| sp|P08579|RU2B\_HUMA | SNRPB2 | RU2B\_HUMAN U2 small nuclear ribonucleoprotein B'' | 8 |
| sp|P62829|RL23\_HUMA | RPL23 | RL23\_HUMAN 60S ribosomal protein L23 | 7 |
| sp|P83731|RL24\_HUMA | RPL24 | RL24\_HUMAN 60S ribosomal protein L24 | 7 |
| sp|P04259|K2C6B\_HUM | KRT6B | K2C6B\_HUMAN\_contaminant Keratin, type II cytoskeletal 6B | 7 |
| sp|P07737|PROF1\_HUM | PFN1 | PROF1\_HUMAN Profilin-1 | 7 |
| sp|P40429|RL13A\_HUM | RPL13A | RL13A\_HUMAN 60S ribosomal protein L13a | 7 |
| sp|Q9UKD2|MRT4\_HUM | MRTO4 | MRT4\_HUMAN mRNA turnover protein 4 homolog | 7 |
| sp|Q9BZI7|REN3B\_HUM | UPF3B | REN3B\_HUMAN Regulator of nonsense transcripts 3B | 7 |
| sp|Q5VWQ0|RSBN1\_HU | RSBN1 | RSBN1\_HUMAN Round spermatid basic protein 1 | 7 |
| sp|P35637|FUS\_HUMAN | FUS | FUS\_HUMAN RNA-binding protein FUS | 7 |
| sp|A8MWD9|RUXGL\_HUMAN | | RUXGL\_HUMAN Small nuclear ribonucleoprotein G-like protein | 7 |
| sp|Q99575|POP1\_HUM | POP1 | POP1\_HUMAN Ribonucleases P/MRP protein subunit POP1 | 7 |
| sp|Q13243|SRSF5\_HUM | SRSF5 | SRSF5\_HUMAN Serine/arginine-rich splicing factor 5 | 7 |
| sp|P51114|FXR1\_HUMA | FXR1 | FXR1\_HUMAN Fragile X mental retardation syndrome-related protein 1 | 7 |
| sp|Q8WXX5|DNJC9\_HU | DNAJC9 | DNJC9\_HUMAN DnaJ homolog subfamily C member 9 | 7 |
| sp|Q00059|TFAM\_HUM | TFAM | TFAM\_HUMAN Transcription factor A, mitochondrial | 7 |
| sp|Q13242|SRSF9\_HUM | SRSF9 | SRSF9\_HUMAN Serine/arginine-rich splicing factor 9 | 7 |
| sp|Q15046|SYK\_HUMA | KARS | SYK\_HUMAN Lysine--tRNA ligase | 7 |
| sp|Q9Y224|CN166\_HUM | C14orf166 | CN166\_HUMAN UPF0568 protein C14orf166 | 7 |
| sp|Q13247|SRSF6\_HUM | SRSF6 | SRSF6\_HUMAN Serine/arginine-rich splicing factor 6 | 7 |
| sp|P83916|CBX1\_HUMA | CBX1 | CBX1\_HUMAN Chromobox protein homolog 1 | 7 |
| sp|Q13263|TIF1B\_HUM | TRIM28 | TIF1B\_HUMAN Transcription intermediary factor 1-beta | 7 |
| sp|O15446-2|RPA34\_HU | CD3EAP | RPA34\_HUMAN Isoform 2 of DNA-directed RNA polymerase I subunit R | 7 |
| sp|Q9H814|PHAX\_HUM | PHAX | PHAX\_HUMAN Phosphorylated adapter RNA export protein | 7 |

## Slide 10
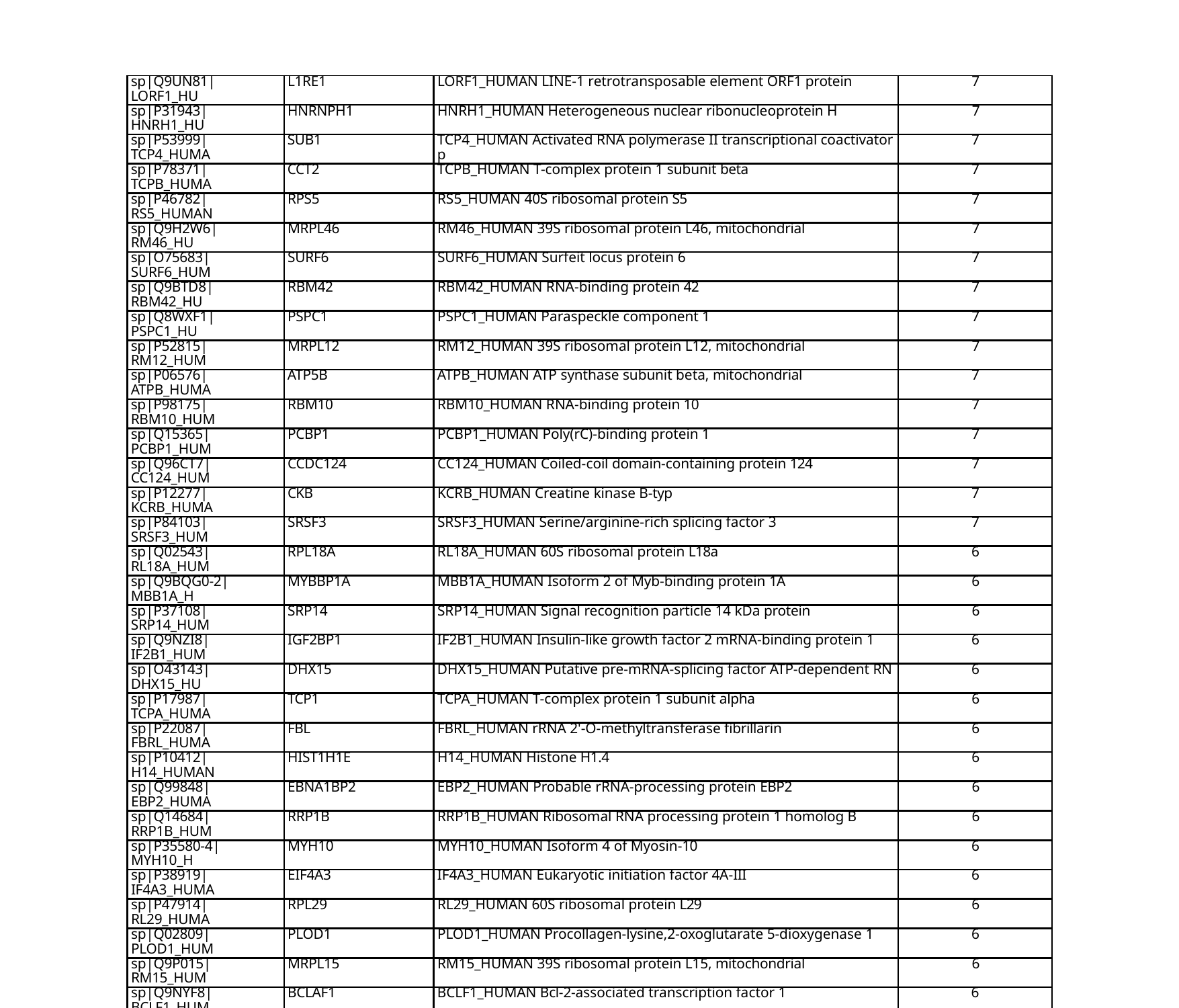

| sp|Q9UN81|LORF1\_HU | L1RE1 | LORF1\_HUMAN LINE-1 retrotransposable element ORF1 protein | 7 |
| --- | --- | --- | --- |
| sp|P31943|HNRH1\_HU | HNRNPH1 | HNRH1\_HUMAN Heterogeneous nuclear ribonucleoprotein H | 7 |
| sp|P53999|TCP4\_HUMA | SUB1 | TCP4\_HUMAN Activated RNA polymerase II transcriptional coactivator p | 7 |
| sp|P78371|TCPB\_HUMA | CCT2 | TCPB\_HUMAN T-complex protein 1 subunit beta | 7 |
| sp|P46782|RS5\_HUMAN | RPS5 | RS5\_HUMAN 40S ribosomal protein S5 | 7 |
| sp|Q9H2W6|RM46\_HU | MRPL46 | RM46\_HUMAN 39S ribosomal protein L46, mitochondrial | 7 |
| sp|O75683|SURF6\_HUM | SURF6 | SURF6\_HUMAN Surfeit locus protein 6 | 7 |
| sp|Q9BTD8|RBM42\_HU | RBM42 | RBM42\_HUMAN RNA-binding protein 42 | 7 |
| sp|Q8WXF1|PSPC1\_HU | PSPC1 | PSPC1\_HUMAN Paraspeckle component 1 | 7 |
| sp|P52815|RM12\_HUM | MRPL12 | RM12\_HUMAN 39S ribosomal protein L12, mitochondrial | 7 |
| sp|P06576|ATPB\_HUMA | ATP5B | ATPB\_HUMAN ATP synthase subunit beta, mitochondrial | 7 |
| sp|P98175|RBM10\_HUM | RBM10 | RBM10\_HUMAN RNA-binding protein 10 | 7 |
| sp|Q15365|PCBP1\_HUM | PCBP1 | PCBP1\_HUMAN Poly(rC)-binding protein 1 | 7 |
| sp|Q96CT7|CC124\_HUM | CCDC124 | CC124\_HUMAN Coiled-coil domain-containing protein 124 | 7 |
| sp|P12277|KCRB\_HUMA | CKB | KCRB\_HUMAN Creatine kinase B-typ | 7 |
| sp|P84103|SRSF3\_HUM | SRSF3 | SRSF3\_HUMAN Serine/arginine-rich splicing factor 3 | 7 |
| sp|Q02543|RL18A\_HUM | RPL18A | RL18A\_HUMAN 60S ribosomal protein L18a | 6 |
| sp|Q9BQG0-2|MBB1A\_H | MYBBP1A | MBB1A\_HUMAN Isoform 2 of Myb-binding protein 1A | 6 |
| sp|P37108|SRP14\_HUM | SRP14 | SRP14\_HUMAN Signal recognition particle 14 kDa protein | 6 |
| sp|Q9NZI8|IF2B1\_HUM | IGF2BP1 | IF2B1\_HUMAN Insulin-like growth factor 2 mRNA-binding protein 1 | 6 |
| sp|O43143|DHX15\_HU | DHX15 | DHX15\_HUMAN Putative pre-mRNA-splicing factor ATP-dependent RN | 6 |
| sp|P17987|TCPA\_HUMA | TCP1 | TCPA\_HUMAN T-complex protein 1 subunit alpha | 6 |
| sp|P22087|FBRL\_HUMA | FBL | FBRL\_HUMAN rRNA 2'-O-methyltransferase fibrillarin | 6 |
| sp|P10412|H14\_HUMAN | HIST1H1E | H14\_HUMAN Histone H1.4 | 6 |
| sp|Q99848|EBP2\_HUMA | EBNA1BP2 | EBP2\_HUMAN Probable rRNA-processing protein EBP2 | 6 |
| sp|Q14684|RRP1B\_HUM | RRP1B | RRP1B\_HUMAN Ribosomal RNA processing protein 1 homolog B | 6 |
| sp|P35580-4|MYH10\_H | MYH10 | MYH10\_HUMAN Isoform 4 of Myosin-10 | 6 |
| sp|P38919|IF4A3\_HUMA | EIF4A3 | IF4A3\_HUMAN Eukaryotic initiation factor 4A-III | 6 |
| sp|P47914|RL29\_HUMA | RPL29 | RL29\_HUMAN 60S ribosomal protein L29 | 6 |
| sp|Q02809|PLOD1\_HUM | PLOD1 | PLOD1\_HUMAN Procollagen-lysine,2-oxoglutarate 5-dioxygenase 1 | 6 |
| sp|Q9P015|RM15\_HUM | MRPL15 | RM15\_HUMAN 39S ribosomal protein L15, mitochondrial | 6 |
| sp|Q9NYF8|BCLF1\_HUM | BCLAF1 | BCLF1\_HUMAN Bcl-2-associated transcription factor 1 | 6 |
| sp|Q9BVP2|GNL3\_HUM | GNL3 | GNL3\_HUMAN Guanine nucleotide-binding protein-like 3 | 6 |
| sp|Q07666|KHDR1\_HU | KHDRBS1 | KHDR1\_HUMAN KH domain-containing, RNA-binding, signal transducti | 6 |
| sp|Q09028|RBBP4\_HUM | RBBP4 | RBBP4\_HUMAN Histone-binding protein RBBP4 | 6 |

## Slide 11
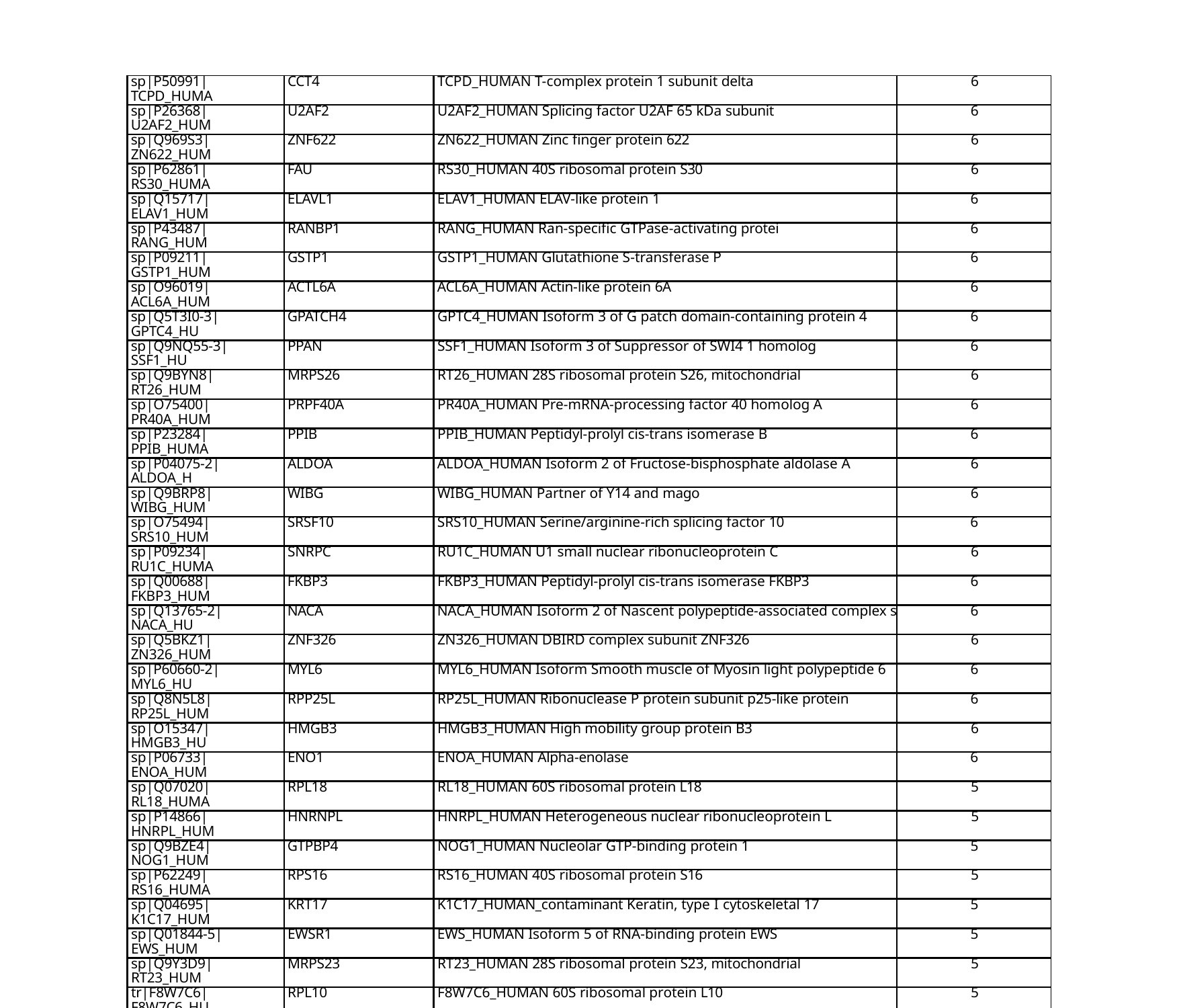

| sp|P50991|TCPD\_HUMA | CCT4 | TCPD\_HUMAN T-complex protein 1 subunit delta | 6 |
| --- | --- | --- | --- |
| sp|P26368|U2AF2\_HUM | U2AF2 | U2AF2\_HUMAN Splicing factor U2AF 65 kDa subunit | 6 |
| sp|Q969S3|ZN622\_HUM | ZNF622 | ZN622\_HUMAN Zinc finger protein 622 | 6 |
| sp|P62861|RS30\_HUMA | FAU | RS30\_HUMAN 40S ribosomal protein S30 | 6 |
| sp|Q15717|ELAV1\_HUM | ELAVL1 | ELAV1\_HUMAN ELAV-like protein 1 | 6 |
| sp|P43487|RANG\_HUM | RANBP1 | RANG\_HUMAN Ran-specific GTPase-activating protei | 6 |
| sp|P09211|GSTP1\_HUM | GSTP1 | GSTP1\_HUMAN Glutathione S-transferase P | 6 |
| sp|O96019|ACL6A\_HUM | ACTL6A | ACL6A\_HUMAN Actin-like protein 6A | 6 |
| sp|Q5T3I0-3|GPTC4\_HU | GPATCH4 | GPTC4\_HUMAN Isoform 3 of G patch domain-containing protein 4 | 6 |
| sp|Q9NQ55-3|SSF1\_HU | PPAN | SSF1\_HUMAN Isoform 3 of Suppressor of SWI4 1 homolog | 6 |
| sp|Q9BYN8|RT26\_HUM | MRPS26 | RT26\_HUMAN 28S ribosomal protein S26, mitochondrial | 6 |
| sp|O75400|PR40A\_HUM | PRPF40A | PR40A\_HUMAN Pre-mRNA-processing factor 40 homolog A | 6 |
| sp|P23284|PPIB\_HUMA | PPIB | PPIB\_HUMAN Peptidyl-prolyl cis-trans isomerase B | 6 |
| sp|P04075-2|ALDOA\_H | ALDOA | ALDOA\_HUMAN Isoform 2 of Fructose-bisphosphate aldolase A | 6 |
| sp|Q9BRP8|WIBG\_HUM | WIBG | WIBG\_HUMAN Partner of Y14 and mago | 6 |
| sp|O75494|SRS10\_HUM | SRSF10 | SRS10\_HUMAN Serine/arginine-rich splicing factor 10 | 6 |
| sp|P09234|RU1C\_HUMA | SNRPC | RU1C\_HUMAN U1 small nuclear ribonucleoprotein C | 6 |
| sp|Q00688|FKBP3\_HUM | FKBP3 | FKBP3\_HUMAN Peptidyl-prolyl cis-trans isomerase FKBP3 | 6 |
| sp|Q13765-2|NACA\_HU | NACA | NACA\_HUMAN Isoform 2 of Nascent polypeptide-associated complex s | 6 |
| sp|Q5BKZ1|ZN326\_HUM | ZNF326 | ZN326\_HUMAN DBIRD complex subunit ZNF326 | 6 |
| sp|P60660-2|MYL6\_HU | MYL6 | MYL6\_HUMAN Isoform Smooth muscle of Myosin light polypeptide 6 | 6 |
| sp|Q8N5L8|RP25L\_HUM | RPP25L | RP25L\_HUMAN Ribonuclease P protein subunit p25-like protein | 6 |
| sp|O15347|HMGB3\_HU | HMGB3 | HMGB3\_HUMAN High mobility group protein B3 | 6 |
| sp|P06733|ENOA\_HUM | ENO1 | ENOA\_HUMAN Alpha-enolase | 6 |
| sp|Q07020|RL18\_HUMA | RPL18 | RL18\_HUMAN 60S ribosomal protein L18 | 5 |
| sp|P14866|HNRPL\_HUM | HNRNPL | HNRPL\_HUMAN Heterogeneous nuclear ribonucleoprotein L | 5 |
| sp|Q9BZE4|NOG1\_HUM | GTPBP4 | NOG1\_HUMAN Nucleolar GTP-binding protein 1 | 5 |
| sp|P62249|RS16\_HUMA | RPS16 | RS16\_HUMAN 40S ribosomal protein S16 | 5 |
| sp|Q04695|K1C17\_HUM | KRT17 | K1C17\_HUMAN\_contaminant Keratin, type I cytoskeletal 17 | 5 |
| sp|Q01844-5|EWS\_HUM | EWSR1 | EWS\_HUMAN Isoform 5 of RNA-binding protein EWS | 5 |
| sp|Q9Y3D9|RT23\_HUM | MRPS23 | RT23\_HUMAN 28S ribosomal protein S23, mitochondrial | 5 |
| tr|F8W7C6|F8W7C6\_HU | RPL10 | F8W7C6\_HUMAN 60S ribosomal protein L10 | 5 |
| sp|P49207|RL34\_HUMA | RPL34 | RL34\_HUMAN 60S ribosomal protein L34 | 5 |
| sp|P62241|RS8\_HUMAN | RPS8 | RS8\_HUMAN 40S ribosomal protein S8 | 5 |
| sp|Q96GA3|LTV1\_HUM | LTV1 | LTV1\_HUMAN Protein LTV1 homolog | 5 |

## Slide 12
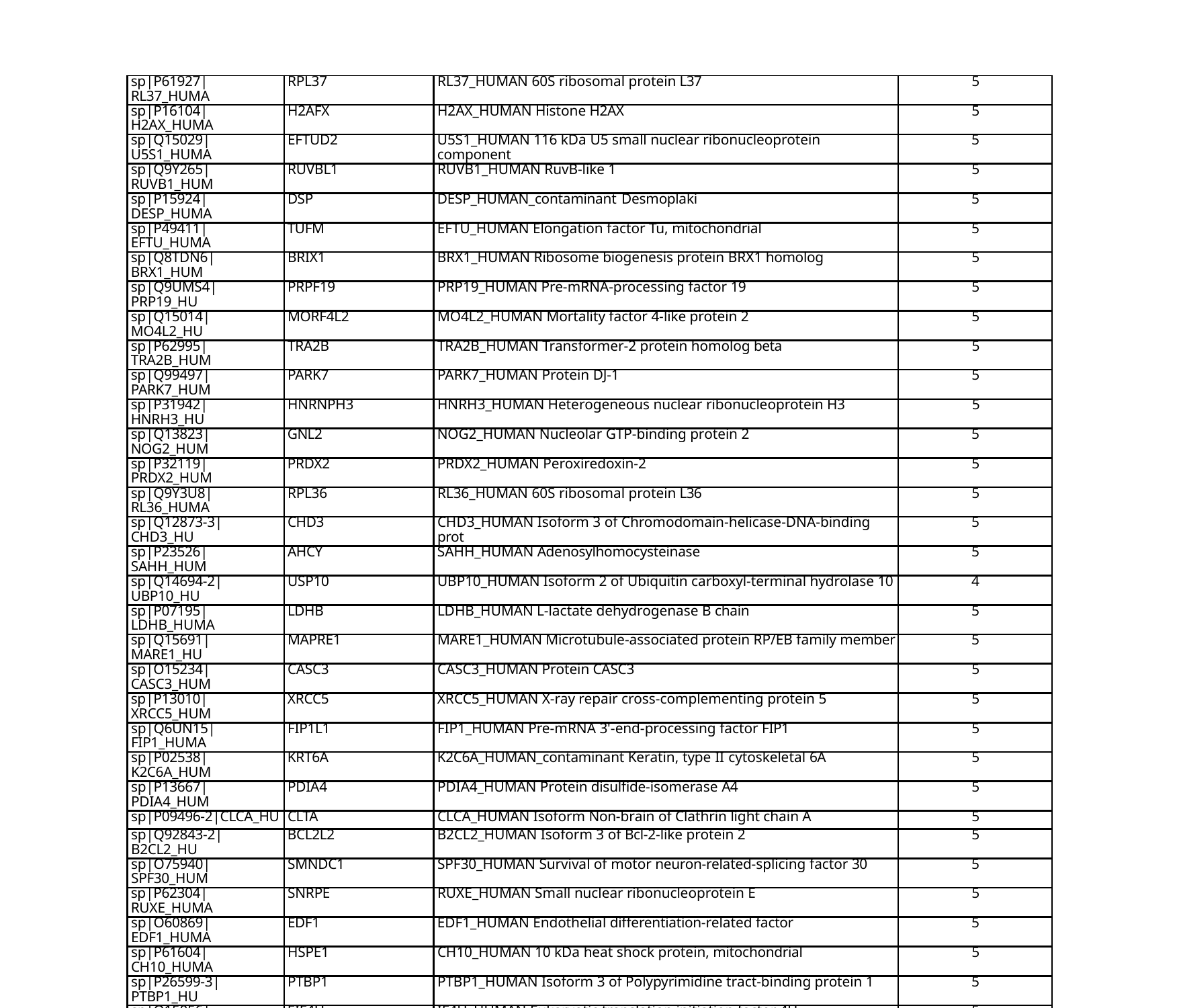

| sp|P61927|RL37\_HUMA | RPL37 | RL37\_HUMAN 60S ribosomal protein L37 | 5 |
| --- | --- | --- | --- |
| sp|P16104|H2AX\_HUMA | H2AFX | H2AX\_HUMAN Histone H2AX | 5 |
| sp|Q15029|U5S1\_HUMA | EFTUD2 | U5S1\_HUMAN 116 kDa U5 small nuclear ribonucleoprotein component | 5 |
| sp|Q9Y265|RUVB1\_HUM | RUVBL1 | RUVB1\_HUMAN RuvB-like 1 | 5 |
| sp|P15924|DESP\_HUMA | DSP | DESP\_HUMAN\_contaminant Desmoplaki | 5 |
| sp|P49411|EFTU\_HUMA | TUFM | EFTU\_HUMAN Elongation factor Tu, mitochondrial | 5 |
| sp|Q8TDN6|BRX1\_HUM | BRIX1 | BRX1\_HUMAN Ribosome biogenesis protein BRX1 homolog | 5 |
| sp|Q9UMS4|PRP19\_HU | PRPF19 | PRP19\_HUMAN Pre-mRNA-processing factor 19 | 5 |
| sp|Q15014|MO4L2\_HU | MORF4L2 | MO4L2\_HUMAN Mortality factor 4-like protein 2 | 5 |
| sp|P62995|TRA2B\_HUM | TRA2B | TRA2B\_HUMAN Transformer-2 protein homolog beta | 5 |
| sp|Q99497|PARK7\_HUM | PARK7 | PARK7\_HUMAN Protein DJ-1 | 5 |
| sp|P31942|HNRH3\_HU | HNRNPH3 | HNRH3\_HUMAN Heterogeneous nuclear ribonucleoprotein H3 | 5 |
| sp|Q13823|NOG2\_HUM | GNL2 | NOG2\_HUMAN Nucleolar GTP-binding protein 2 | 5 |
| sp|P32119|PRDX2\_HUM | PRDX2 | PRDX2\_HUMAN Peroxiredoxin-2 | 5 |
| sp|Q9Y3U8|RL36\_HUMA | RPL36 | RL36\_HUMAN 60S ribosomal protein L36 | 5 |
| sp|Q12873-3|CHD3\_HU | CHD3 | CHD3\_HUMAN Isoform 3 of Chromodomain-helicase-DNA-binding prot | 5 |
| sp|P23526|SAHH\_HUM | AHCY | SAHH\_HUMAN Adenosylhomocysteinase | 5 |
| sp|Q14694-2|UBP10\_HU | USP10 | UBP10\_HUMAN Isoform 2 of Ubiquitin carboxyl-terminal hydrolase 10 | 4 |
| sp|P07195|LDHB\_HUMA | LDHB | LDHB\_HUMAN L-lactate dehydrogenase B chain | 5 |
| sp|Q15691|MARE1\_HU | MAPRE1 | MARE1\_HUMAN Microtubule-associated protein RP/EB family member | 5 |
| sp|O15234|CASC3\_HUM | CASC3 | CASC3\_HUMAN Protein CASC3 | 5 |
| sp|P13010|XRCC5\_HUM | XRCC5 | XRCC5\_HUMAN X-ray repair cross-complementing protein 5 | 5 |
| sp|Q6UN15|FIP1\_HUMA | FIP1L1 | FIP1\_HUMAN Pre-mRNA 3'-end-processing factor FIP1 | 5 |
| sp|P02538|K2C6A\_HUM | KRT6A | K2C6A\_HUMAN\_contaminant Keratin, type II cytoskeletal 6A | 5 |
| sp|P13667|PDIA4\_HUM | PDIA4 | PDIA4\_HUMAN Protein disulfide-isomerase A4 | 5 |
| sp|P09496-2|CLCA\_HU | CLTA | CLCA\_HUMAN Isoform Non-brain of Clathrin light chain A | 5 |
| sp|Q92843-2|B2CL2\_HU | BCL2L2 | B2CL2\_HUMAN Isoform 3 of Bcl-2-like protein 2 | 5 |
| sp|O75940|SPF30\_HUM | SMNDC1 | SPF30\_HUMAN Survival of motor neuron-related-splicing factor 30 | 5 |
| sp|P62304|RUXE\_HUMA | SNRPE | RUXE\_HUMAN Small nuclear ribonucleoprotein E | 5 |
| sp|O60869|EDF1\_HUMA | EDF1 | EDF1\_HUMAN Endothelial differentiation-related factor | 5 |
| sp|P61604|CH10\_HUMA | HSPE1 | CH10\_HUMAN 10 kDa heat shock protein, mitochondrial | 5 |
| sp|P26599-3|PTBP1\_HU | PTBP1 | PTBP1\_HUMAN Isoform 3 of Polypyrimidine tract-binding protein 1 | 5 |
| sp|Q15056|IF4H\_HUMA | EIF4H | IF4H\_HUMAN Eukaryotic translation initiation factor 4H | 5 |
| sp|P22392-2|NDKB\_HU | NME2 | NDKB\_HUMAN Isoform 3 of Nucleoside diphosphate kinase B | 5 |
| sp|Q13151|ROA0\_HUM | HNRNPA0 | ROA0\_HUMAN Heterogeneous nuclear ribonucleoprotein A0 | 5 |

## Slide 13
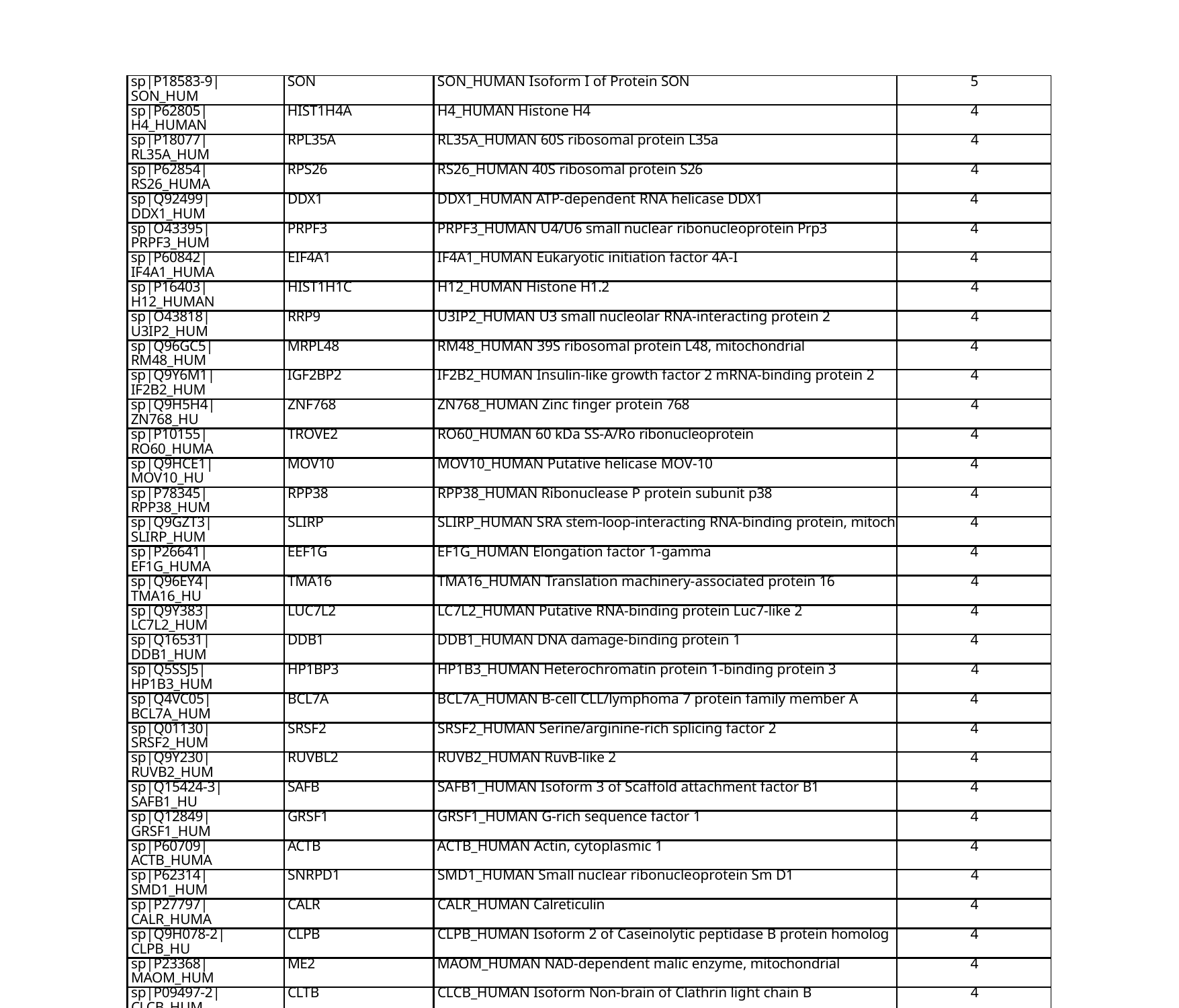

| sp|P18583-9|SON\_HUM | SON | SON\_HUMAN Isoform I of Protein SON | 5 |
| --- | --- | --- | --- |
| sp|P62805|H4\_HUMAN | HIST1H4A | H4\_HUMAN Histone H4 | 4 |
| sp|P18077|RL35A\_HUM | RPL35A | RL35A\_HUMAN 60S ribosomal protein L35a | 4 |
| sp|P62854|RS26\_HUMA | RPS26 | RS26\_HUMAN 40S ribosomal protein S26 | 4 |
| sp|Q92499|DDX1\_HUM | DDX1 | DDX1\_HUMAN ATP-dependent RNA helicase DDX1 | 4 |
| sp|O43395|PRPF3\_HUM | PRPF3 | PRPF3\_HUMAN U4/U6 small nuclear ribonucleoprotein Prp3 | 4 |
| sp|P60842|IF4A1\_HUMA | EIF4A1 | IF4A1\_HUMAN Eukaryotic initiation factor 4A-I | 4 |
| sp|P16403|H12\_HUMAN | HIST1H1C | H12\_HUMAN Histone H1.2 | 4 |
| sp|O43818|U3IP2\_HUM | RRP9 | U3IP2\_HUMAN U3 small nucleolar RNA-interacting protein 2 | 4 |
| sp|Q96GC5|RM48\_HUM | MRPL48 | RM48\_HUMAN 39S ribosomal protein L48, mitochondrial | 4 |
| sp|Q9Y6M1|IF2B2\_HUM | IGF2BP2 | IF2B2\_HUMAN Insulin-like growth factor 2 mRNA-binding protein 2 | 4 |
| sp|Q9H5H4|ZN768\_HU | ZNF768 | ZN768\_HUMAN Zinc finger protein 768 | 4 |
| sp|P10155|RO60\_HUMA | TROVE2 | RO60\_HUMAN 60 kDa SS-A/Ro ribonucleoprotein | 4 |
| sp|Q9HCE1|MOV10\_HU | MOV10 | MOV10\_HUMAN Putative helicase MOV-10 | 4 |
| sp|P78345|RPP38\_HUM | RPP38 | RPP38\_HUMAN Ribonuclease P protein subunit p38 | 4 |
| sp|Q9GZT3|SLIRP\_HUM | SLIRP | SLIRP\_HUMAN SRA stem-loop-interacting RNA-binding protein, mitoch | 4 |
| sp|P26641|EF1G\_HUMA | EEF1G | EF1G\_HUMAN Elongation factor 1-gamma | 4 |
| sp|Q96EY4|TMA16\_HU | TMA16 | TMA16\_HUMAN Translation machinery-associated protein 16 | 4 |
| sp|Q9Y383|LC7L2\_HUM | LUC7L2 | LC7L2\_HUMAN Putative RNA-binding protein Luc7-like 2 | 4 |
| sp|Q16531|DDB1\_HUM | DDB1 | DDB1\_HUMAN DNA damage-binding protein 1 | 4 |
| sp|Q5SSJ5|HP1B3\_HUM | HP1BP3 | HP1B3\_HUMAN Heterochromatin protein 1-binding protein 3 | 4 |
| sp|Q4VC05|BCL7A\_HUM | BCL7A | BCL7A\_HUMAN B-cell CLL/lymphoma 7 protein family member A | 4 |
| sp|Q01130|SRSF2\_HUM | SRSF2 | SRSF2\_HUMAN Serine/arginine-rich splicing factor 2 | 4 |
| sp|Q9Y230|RUVB2\_HUM | RUVBL2 | RUVB2\_HUMAN RuvB-like 2 | 4 |
| sp|Q15424-3|SAFB1\_HU | SAFB | SAFB1\_HUMAN Isoform 3 of Scaffold attachment factor B1 | 4 |
| sp|Q12849|GRSF1\_HUM | GRSF1 | GRSF1\_HUMAN G-rich sequence factor 1 | 4 |
| sp|P60709|ACTB\_HUMA | ACTB | ACTB\_HUMAN Actin, cytoplasmic 1 | 4 |
| sp|P62314|SMD1\_HUM | SNRPD1 | SMD1\_HUMAN Small nuclear ribonucleoprotein Sm D1 | 4 |
| sp|P27797|CALR\_HUMA | CALR | CALR\_HUMAN Calreticulin | 4 |
| sp|Q9H078-2|CLPB\_HU | CLPB | CLPB\_HUMAN Isoform 2 of Caseinolytic peptidase B protein homolog | 4 |
| sp|P23368|MAOM\_HUM | ME2 | MAOM\_HUMAN NAD-dependent malic enzyme, mitochondrial | 4 |
| sp|P09497-2|CLCB\_HUM | CLTB | CLCB\_HUMAN Isoform Non-brain of Clathrin light chain B | 4 |
| sp|Q9BS26|ERP44\_HUM | ERP44 | ERP44\_HUMAN Endoplasmic reticulum resident protein 44 | 4 |
| sp|P31948|STIP1\_HUMA | STIP1 | STIP1\_HUMAN Stress-induced-phosphoprotein 1 | 4 |
| sp|P30405|PPIF\_HUMA | PPIF | PPIF\_HUMAN Peptidyl-prolyl cis-trans isomerase F, mitochondria | 4 |

## Slide 14
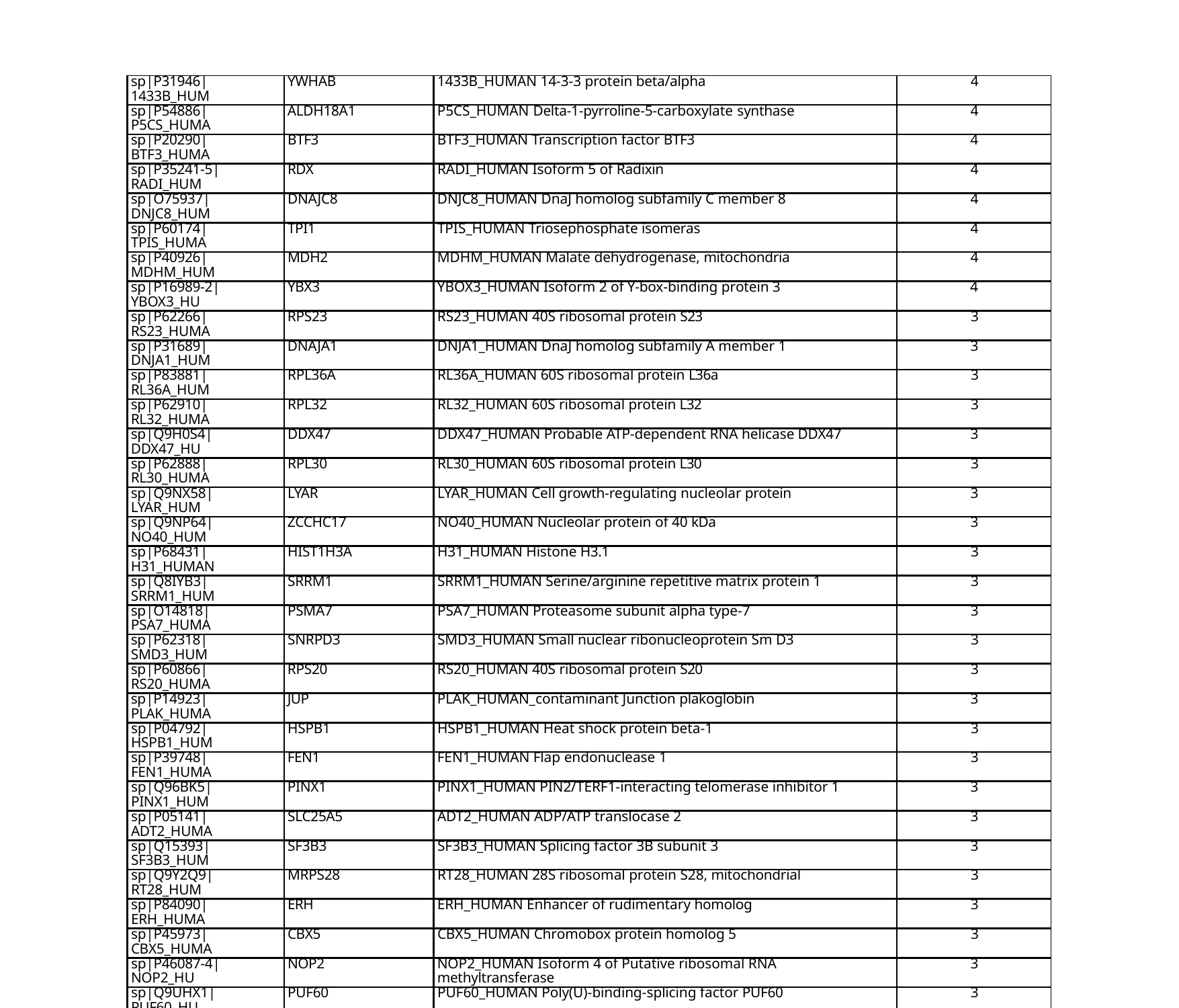

| sp|P31946|1433B\_HUM | YWHAB | 1433B\_HUMAN 14-3-3 protein beta/alpha | 4 |
| --- | --- | --- | --- |
| sp|P54886|P5CS\_HUMA | ALDH18A1 | P5CS\_HUMAN Delta-1-pyrroline-5-carboxylate synthase | 4 |
| sp|P20290|BTF3\_HUMA | BTF3 | BTF3\_HUMAN Transcription factor BTF3 | 4 |
| sp|P35241-5|RADI\_HUM | RDX | RADI\_HUMAN Isoform 5 of Radixin | 4 |
| sp|O75937|DNJC8\_HUM | DNAJC8 | DNJC8\_HUMAN DnaJ homolog subfamily C member 8 | 4 |
| sp|P60174|TPIS\_HUMA | TPI1 | TPIS\_HUMAN Triosephosphate isomeras | 4 |
| sp|P40926|MDHM\_HUM | MDH2 | MDHM\_HUMAN Malate dehydrogenase, mitochondria | 4 |
| sp|P16989-2|YBOX3\_HU | YBX3 | YBOX3\_HUMAN Isoform 2 of Y-box-binding protein 3 | 4 |
| sp|P62266|RS23\_HUMA | RPS23 | RS23\_HUMAN 40S ribosomal protein S23 | 3 |
| sp|P31689|DNJA1\_HUM | DNAJA1 | DNJA1\_HUMAN DnaJ homolog subfamily A member 1 | 3 |
| sp|P83881|RL36A\_HUM | RPL36A | RL36A\_HUMAN 60S ribosomal protein L36a | 3 |
| sp|P62910|RL32\_HUMA | RPL32 | RL32\_HUMAN 60S ribosomal protein L32 | 3 |
| sp|Q9H0S4|DDX47\_HU | DDX47 | DDX47\_HUMAN Probable ATP-dependent RNA helicase DDX47 | 3 |
| sp|P62888|RL30\_HUMA | RPL30 | RL30\_HUMAN 60S ribosomal protein L30 | 3 |
| sp|Q9NX58|LYAR\_HUM | LYAR | LYAR\_HUMAN Cell growth-regulating nucleolar protein | 3 |
| sp|Q9NP64|NO40\_HUM | ZCCHC17 | NO40\_HUMAN Nucleolar protein of 40 kDa | 3 |
| sp|P68431|H31\_HUMAN | HIST1H3A | H31\_HUMAN Histone H3.1 | 3 |
| sp|Q8IYB3|SRRM1\_HUM | SRRM1 | SRRM1\_HUMAN Serine/arginine repetitive matrix protein 1 | 3 |
| sp|O14818|PSA7\_HUMA | PSMA7 | PSA7\_HUMAN Proteasome subunit alpha type-7 | 3 |
| sp|P62318|SMD3\_HUM | SNRPD3 | SMD3\_HUMAN Small nuclear ribonucleoprotein Sm D3 | 3 |
| sp|P60866|RS20\_HUMA | RPS20 | RS20\_HUMAN 40S ribosomal protein S20 | 3 |
| sp|P14923|PLAK\_HUMA | JUP | PLAK\_HUMAN\_contaminant Junction plakoglobin | 3 |
| sp|P04792|HSPB1\_HUM | HSPB1 | HSPB1\_HUMAN Heat shock protein beta-1 | 3 |
| sp|P39748|FEN1\_HUMA | FEN1 | FEN1\_HUMAN Flap endonuclease 1 | 3 |
| sp|Q96BK5|PINX1\_HUM | PINX1 | PINX1\_HUMAN PIN2/TERF1-interacting telomerase inhibitor 1 | 3 |
| sp|P05141|ADT2\_HUMA | SLC25A5 | ADT2\_HUMAN ADP/ATP translocase 2 | 3 |
| sp|Q15393|SF3B3\_HUM | SF3B3 | SF3B3\_HUMAN Splicing factor 3B subunit 3 | 3 |
| sp|Q9Y2Q9|RT28\_HUM | MRPS28 | RT28\_HUMAN 28S ribosomal protein S28, mitochondrial | 3 |
| sp|P84090|ERH\_HUMA | ERH | ERH\_HUMAN Enhancer of rudimentary homolog | 3 |
| sp|P45973|CBX5\_HUMA | CBX5 | CBX5\_HUMAN Chromobox protein homolog 5 | 3 |
| sp|P46087-4|NOP2\_HU | NOP2 | NOP2\_HUMAN Isoform 4 of Putative ribosomal RNA methyltransferase | 3 |
| sp|Q9UHX1|PUF60\_HU | PUF60 | PUF60\_HUMAN Poly(U)-binding-splicing factor PUF60 | 3 |
| sp|P48643|TCPE\_HUMA | CCT5 | TCPE\_HUMAN T-complex protein 1 subunit epsilon | 3 |
| sp|Q9Y5B9|SP16H\_HUM | SUPT16H | SP16H\_HUMAN FACT complex subunit SPT16 | 3 |
| sp|P82663|RT25\_HUMA | MRPS25 | RT25\_HUMAN 28S ribosomal protein S25, mitochondrial | 3 |

## Slide 15
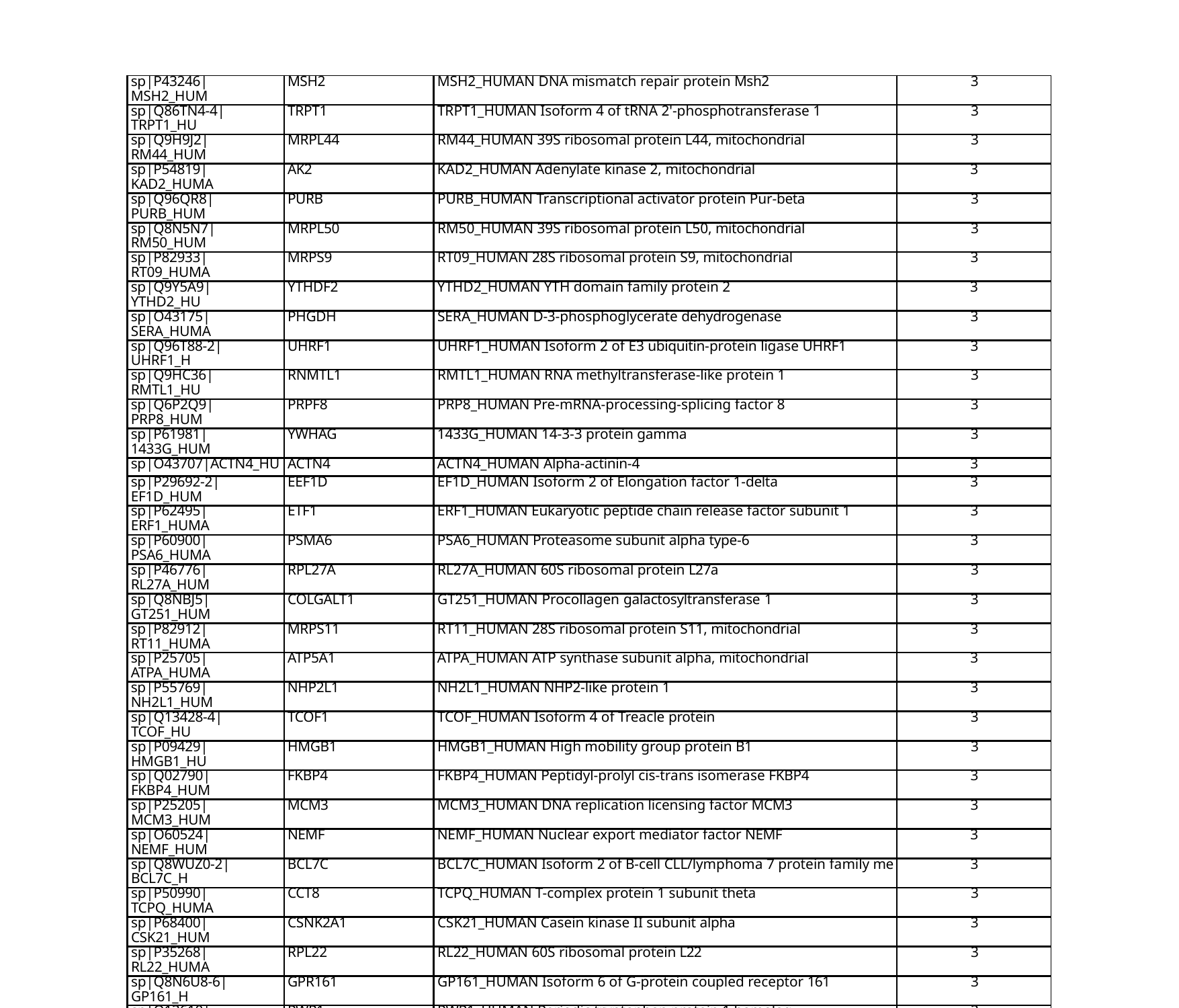

| sp|P43246|MSH2\_HUM | MSH2 | MSH2\_HUMAN DNA mismatch repair protein Msh2 | 3 |
| --- | --- | --- | --- |
| sp|Q86TN4-4|TRPT1\_HU | TRPT1 | TRPT1\_HUMAN Isoform 4 of tRNA 2'-phosphotransferase 1 | 3 |
| sp|Q9H9J2|RM44\_HUM | MRPL44 | RM44\_HUMAN 39S ribosomal protein L44, mitochondrial | 3 |
| sp|P54819|KAD2\_HUMA | AK2 | KAD2\_HUMAN Adenylate kinase 2, mitochondrial | 3 |
| sp|Q96QR8|PURB\_HUM | PURB | PURB\_HUMAN Transcriptional activator protein Pur-beta | 3 |
| sp|Q8N5N7|RM50\_HUM | MRPL50 | RM50\_HUMAN 39S ribosomal protein L50, mitochondrial | 3 |
| sp|P82933|RT09\_HUMA | MRPS9 | RT09\_HUMAN 28S ribosomal protein S9, mitochondrial | 3 |
| sp|Q9Y5A9|YTHD2\_HU | YTHDF2 | YTHD2\_HUMAN YTH domain family protein 2 | 3 |
| sp|O43175|SERA\_HUMA | PHGDH | SERA\_HUMAN D-3-phosphoglycerate dehydrogenase | 3 |
| sp|Q96T88-2|UHRF1\_H | UHRF1 | UHRF1\_HUMAN Isoform 2 of E3 ubiquitin-protein ligase UHRF1 | 3 |
| sp|Q9HC36|RMTL1\_HU | RNMTL1 | RMTL1\_HUMAN RNA methyltransferase-like protein 1 | 3 |
| sp|Q6P2Q9|PRP8\_HUM | PRPF8 | PRP8\_HUMAN Pre-mRNA-processing-splicing factor 8 | 3 |
| sp|P61981|1433G\_HUM | YWHAG | 1433G\_HUMAN 14-3-3 protein gamma | 3 |
| sp|O43707|ACTN4\_HU | ACTN4 | ACTN4\_HUMAN Alpha-actinin-4 | 3 |
| sp|P29692-2|EF1D\_HUM | EEF1D | EF1D\_HUMAN Isoform 2 of Elongation factor 1-delta | 3 |
| sp|P62495|ERF1\_HUMA | ETF1 | ERF1\_HUMAN Eukaryotic peptide chain release factor subunit 1 | 3 |
| sp|P60900|PSA6\_HUMA | PSMA6 | PSA6\_HUMAN Proteasome subunit alpha type-6 | 3 |
| sp|P46776|RL27A\_HUM | RPL27A | RL27A\_HUMAN 60S ribosomal protein L27a | 3 |
| sp|Q8NBJ5|GT251\_HUM | COLGALT1 | GT251\_HUMAN Procollagen galactosyltransferase 1 | 3 |
| sp|P82912|RT11\_HUMA | MRPS11 | RT11\_HUMAN 28S ribosomal protein S11, mitochondrial | 3 |
| sp|P25705|ATPA\_HUMA | ATP5A1 | ATPA\_HUMAN ATP synthase subunit alpha, mitochondrial | 3 |
| sp|P55769|NH2L1\_HUM | NHP2L1 | NH2L1\_HUMAN NHP2-like protein 1 | 3 |
| sp|Q13428-4|TCOF\_HU | TCOF1 | TCOF\_HUMAN Isoform 4 of Treacle protein | 3 |
| sp|P09429|HMGB1\_HU | HMGB1 | HMGB1\_HUMAN High mobility group protein B1 | 3 |
| sp|Q02790|FKBP4\_HUM | FKBP4 | FKBP4\_HUMAN Peptidyl-prolyl cis-trans isomerase FKBP4 | 3 |
| sp|P25205|MCM3\_HUM | MCM3 | MCM3\_HUMAN DNA replication licensing factor MCM3 | 3 |
| sp|O60524|NEMF\_HUM | NEMF | NEMF\_HUMAN Nuclear export mediator factor NEMF | 3 |
| sp|Q8WUZ0-2|BCL7C\_H | BCL7C | BCL7C\_HUMAN Isoform 2 of B-cell CLL/lymphoma 7 protein family me | 3 |
| sp|P50990|TCPQ\_HUMA | CCT8 | TCPQ\_HUMAN T-complex protein 1 subunit theta | 3 |
| sp|P68400|CSK21\_HUM | CSNK2A1 | CSK21\_HUMAN Casein kinase II subunit alpha | 3 |
| sp|P35268|RL22\_HUMA | RPL22 | RL22\_HUMAN 60S ribosomal protein L22 | 3 |
| sp|Q8N6U8-6|GP161\_H | GPR161 | GP161\_HUMAN Isoform 6 of G-protein coupled receptor 161 | 3 |
| sp|Q13610|PWP1\_HUM | PWP1 | PWP1\_HUMAN Periodic tryptophan protein 1 homolog | 3 |
| sp|P30048|PRDX3\_HUM | PRDX3 | PRDX3\_HUMAN Thioredoxin-dependent peroxide reductase, mitochond | 3 |
| sp|Q96DI7|SNR40\_HUM | SNRNP40 | SNR40\_HUMAN U5 small nuclear ribonucleoprotein 40 kDa protein | 3 |

## Slide 16
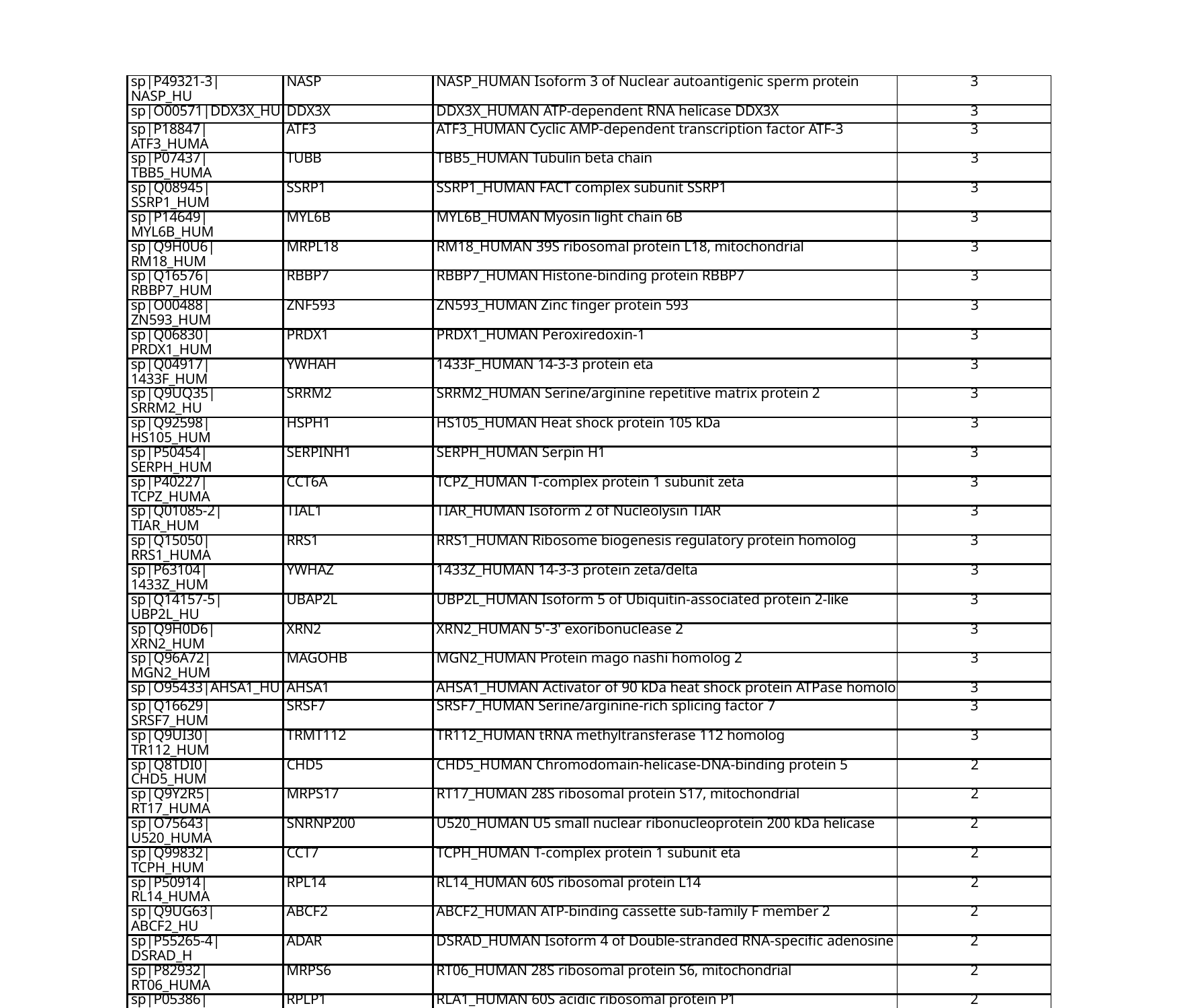

| sp|P49321-3|NASP\_HU | NASP | NASP\_HUMAN Isoform 3 of Nuclear autoantigenic sperm protein | 3 |
| --- | --- | --- | --- |
| sp|O00571|DDX3X\_HU | DDX3X | DDX3X\_HUMAN ATP-dependent RNA helicase DDX3X | 3 |
| sp|P18847|ATF3\_HUMA | ATF3 | ATF3\_HUMAN Cyclic AMP-dependent transcription factor ATF-3 | 3 |
| sp|P07437|TBB5\_HUMA | TUBB | TBB5\_HUMAN Tubulin beta chain | 3 |
| sp|Q08945|SSRP1\_HUM | SSRP1 | SSRP1\_HUMAN FACT complex subunit SSRP1 | 3 |
| sp|P14649|MYL6B\_HUM | MYL6B | MYL6B\_HUMAN Myosin light chain 6B | 3 |
| sp|Q9H0U6|RM18\_HUM | MRPL18 | RM18\_HUMAN 39S ribosomal protein L18, mitochondrial | 3 |
| sp|Q16576|RBBP7\_HUM | RBBP7 | RBBP7\_HUMAN Histone-binding protein RBBP7 | 3 |
| sp|O00488|ZN593\_HUM | ZNF593 | ZN593\_HUMAN Zinc finger protein 593 | 3 |
| sp|Q06830|PRDX1\_HUM | PRDX1 | PRDX1\_HUMAN Peroxiredoxin-1 | 3 |
| sp|Q04917|1433F\_HUM | YWHAH | 1433F\_HUMAN 14-3-3 protein eta | 3 |
| sp|Q9UQ35|SRRM2\_HU | SRRM2 | SRRM2\_HUMAN Serine/arginine repetitive matrix protein 2 | 3 |
| sp|Q92598|HS105\_HUM | HSPH1 | HS105\_HUMAN Heat shock protein 105 kDa | 3 |
| sp|P50454|SERPH\_HUM | SERPINH1 | SERPH\_HUMAN Serpin H1 | 3 |
| sp|P40227|TCPZ\_HUMA | CCT6A | TCPZ\_HUMAN T-complex protein 1 subunit zeta | 3 |
| sp|Q01085-2|TIAR\_HUM | TIAL1 | TIAR\_HUMAN Isoform 2 of Nucleolysin TIAR | 3 |
| sp|Q15050|RRS1\_HUMA | RRS1 | RRS1\_HUMAN Ribosome biogenesis regulatory protein homolog | 3 |
| sp|P63104|1433Z\_HUM | YWHAZ | 1433Z\_HUMAN 14-3-3 protein zeta/delta | 3 |
| sp|Q14157-5|UBP2L\_HU | UBAP2L | UBP2L\_HUMAN Isoform 5 of Ubiquitin-associated protein 2-like | 3 |
| sp|Q9H0D6|XRN2\_HUM | XRN2 | XRN2\_HUMAN 5'-3' exoribonuclease 2 | 3 |
| sp|Q96A72|MGN2\_HUM | MAGOHB | MGN2\_HUMAN Protein mago nashi homolog 2 | 3 |
| sp|O95433|AHSA1\_HU | AHSA1 | AHSA1\_HUMAN Activator of 90 kDa heat shock protein ATPase homolo | 3 |
| sp|Q16629|SRSF7\_HUM | SRSF7 | SRSF7\_HUMAN Serine/arginine-rich splicing factor 7 | 3 |
| sp|Q9UI30|TR112\_HUM | TRMT112 | TR112\_HUMAN tRNA methyltransferase 112 homolog | 3 |
| sp|Q8TDI0|CHD5\_HUM | CHD5 | CHD5\_HUMAN Chromodomain-helicase-DNA-binding protein 5 | 2 |
| sp|Q9Y2R5|RT17\_HUMA | MRPS17 | RT17\_HUMAN 28S ribosomal protein S17, mitochondrial | 2 |
| sp|O75643|U520\_HUMA | SNRNP200 | U520\_HUMAN U5 small nuclear ribonucleoprotein 200 kDa helicase | 2 |
| sp|Q99832|TCPH\_HUM | CCT7 | TCPH\_HUMAN T-complex protein 1 subunit eta | 2 |
| sp|P50914|RL14\_HUMA | RPL14 | RL14\_HUMAN 60S ribosomal protein L14 | 2 |
| sp|Q9UG63|ABCF2\_HU | ABCF2 | ABCF2\_HUMAN ATP-binding cassette sub-family F member 2 | 2 |
| sp|P55265-4|DSRAD\_H | ADAR | DSRAD\_HUMAN Isoform 4 of Double-stranded RNA-specific adenosine | 2 |
| sp|P82932|RT06\_HUMA | MRPS6 | RT06\_HUMAN 28S ribosomal protein S6, mitochondrial | 2 |
| sp|P05386|RLA1\_HUMA | RPLP1 | RLA1\_HUMAN 60S acidic ribosomal protein P1 | 2 |
| sp|P82675|RT05\_HUMA | MRPS5 | RT05\_HUMAN 28S ribosomal protein S5, mitochondrial | 2 |
| sp|P26358-2|DNMT1\_H | DNMT1 | DNMT1\_HUMAN Isoform 2 of DNA (cytosine-5)-methyltransferase 1 | 2 |

## Slide 17
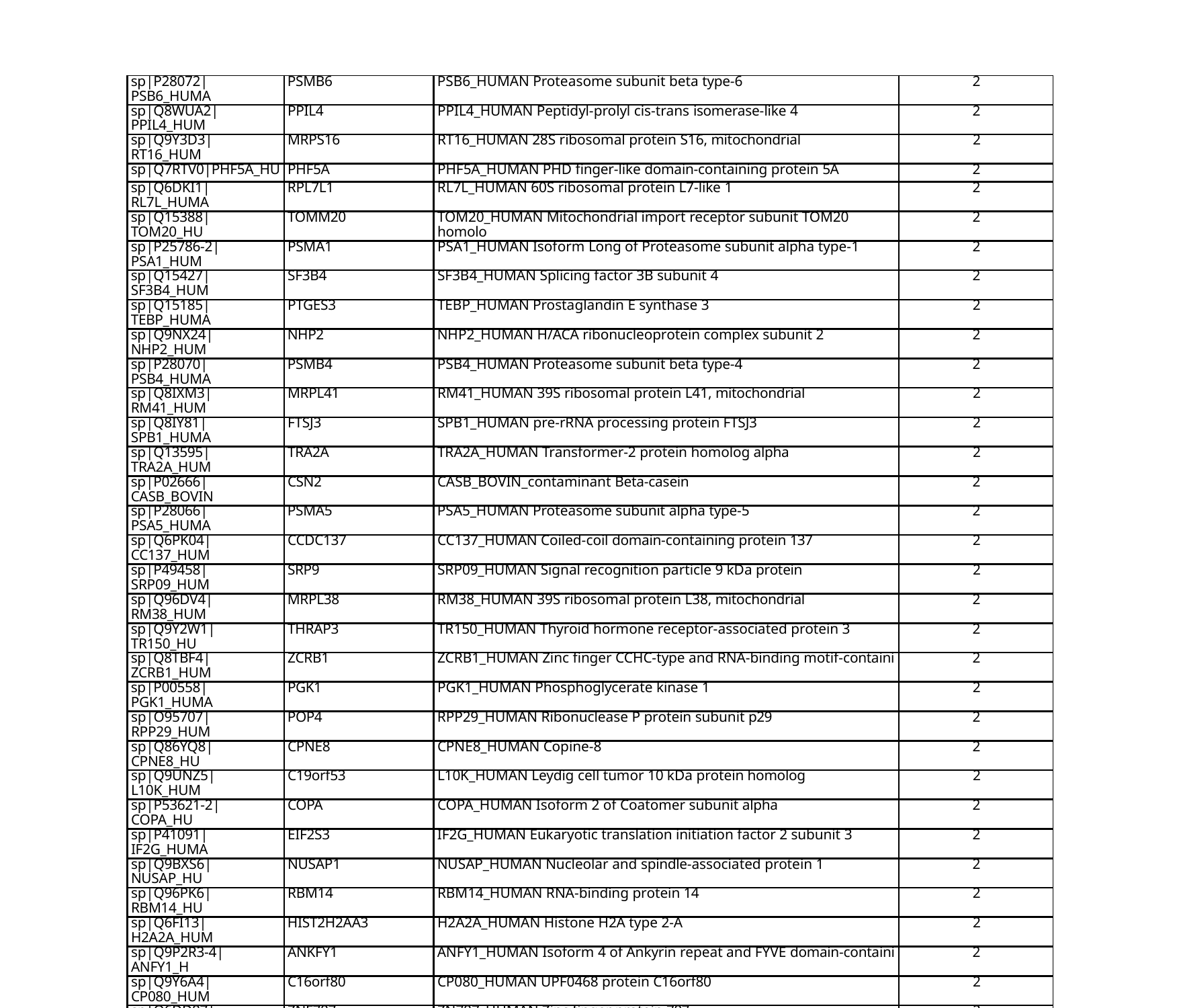

| sp|P28072|PSB6\_HUMA | PSMB6 | PSB6\_HUMAN Proteasome subunit beta type-6 | 2 |
| --- | --- | --- | --- |
| sp|Q8WUA2|PPIL4\_HUM | PPIL4 | PPIL4\_HUMAN Peptidyl-prolyl cis-trans isomerase-like 4 | 2 |
| sp|Q9Y3D3|RT16\_HUM | MRPS16 | RT16\_HUMAN 28S ribosomal protein S16, mitochondrial | 2 |
| sp|Q7RTV0|PHF5A\_HU | PHF5A | PHF5A\_HUMAN PHD finger-like domain-containing protein 5A | 2 |
| sp|Q6DKI1|RL7L\_HUMA | RPL7L1 | RL7L\_HUMAN 60S ribosomal protein L7-like 1 | 2 |
| sp|Q15388|TOM20\_HU | TOMM20 | TOM20\_HUMAN Mitochondrial import receptor subunit TOM20 homolo | 2 |
| sp|P25786-2|PSA1\_HUM | PSMA1 | PSA1\_HUMAN Isoform Long of Proteasome subunit alpha type-1 | 2 |
| sp|Q15427|SF3B4\_HUM | SF3B4 | SF3B4\_HUMAN Splicing factor 3B subunit 4 | 2 |
| sp|Q15185|TEBP\_HUMA | PTGES3 | TEBP\_HUMAN Prostaglandin E synthase 3 | 2 |
| sp|Q9NX24|NHP2\_HUM | NHP2 | NHP2\_HUMAN H/ACA ribonucleoprotein complex subunit 2 | 2 |
| sp|P28070|PSB4\_HUMA | PSMB4 | PSB4\_HUMAN Proteasome subunit beta type-4 | 2 |
| sp|Q8IXM3|RM41\_HUM | MRPL41 | RM41\_HUMAN 39S ribosomal protein L41, mitochondrial | 2 |
| sp|Q8IY81|SPB1\_HUMA | FTSJ3 | SPB1\_HUMAN pre-rRNA processing protein FTSJ3 | 2 |
| sp|Q13595|TRA2A\_HUM | TRA2A | TRA2A\_HUMAN Transformer-2 protein homolog alpha | 2 |
| sp|P02666|CASB\_BOVIN | CSN2 | CASB\_BOVIN\_contaminant Beta-casein | 2 |
| sp|P28066|PSA5\_HUMA | PSMA5 | PSA5\_HUMAN Proteasome subunit alpha type-5 | 2 |
| sp|Q6PK04|CC137\_HUM | CCDC137 | CC137\_HUMAN Coiled-coil domain-containing protein 137 | 2 |
| sp|P49458|SRP09\_HUM | SRP9 | SRP09\_HUMAN Signal recognition particle 9 kDa protein | 2 |
| sp|Q96DV4|RM38\_HUM | MRPL38 | RM38\_HUMAN 39S ribosomal protein L38, mitochondrial | 2 |
| sp|Q9Y2W1|TR150\_HU | THRAP3 | TR150\_HUMAN Thyroid hormone receptor-associated protein 3 | 2 |
| sp|Q8TBF4|ZCRB1\_HUM | ZCRB1 | ZCRB1\_HUMAN Zinc finger CCHC-type and RNA-binding motif-containi | 2 |
| sp|P00558|PGK1\_HUMA | PGK1 | PGK1\_HUMAN Phosphoglycerate kinase 1 | 2 |
| sp|O95707|RPP29\_HUM | POP4 | RPP29\_HUMAN Ribonuclease P protein subunit p29 | 2 |
| sp|Q86YQ8|CPNE8\_HU | CPNE8 | CPNE8\_HUMAN Copine-8 | 2 |
| sp|Q9UNZ5|L10K\_HUM | C19orf53 | L10K\_HUMAN Leydig cell tumor 10 kDa protein homolog | 2 |
| sp|P53621-2|COPA\_HU | COPA | COPA\_HUMAN Isoform 2 of Coatomer subunit alpha | 2 |
| sp|P41091|IF2G\_HUMA | EIF2S3 | IF2G\_HUMAN Eukaryotic translation initiation factor 2 subunit 3 | 2 |
| sp|Q9BXS6|NUSAP\_HU | NUSAP1 | NUSAP\_HUMAN Nucleolar and spindle-associated protein 1 | 2 |
| sp|Q96PK6|RBM14\_HU | RBM14 | RBM14\_HUMAN RNA-binding protein 14 | 2 |
| sp|Q6FI13|H2A2A\_HUM | HIST2H2AA3 | H2A2A\_HUMAN Histone H2A type 2-A | 2 |
| sp|Q9P2R3-4|ANFY1\_H | ANKFY1 | ANFY1\_HUMAN Isoform 4 of Ankyrin repeat and FYVE domain-containi | 2 |
| sp|Q9Y6A4|CP080\_HUM | C16orf80 | CP080\_HUMAN UPF0468 protein C16orf80 | 2 |
| sp|Q6DD87|ZN787\_HU | ZNF787 | ZN787\_HUMAN Zinc finger protein 787 | 2 |
| sp|O15226-2|NKRF\_HU | NKRF | NKRF\_HUMAN Isoform 2 of NF-kappa-B-repressing factor | 2 |
| sp|Q7Z739|YTHD3\_HUM | YTHDF3 | YTHD3\_HUMAN YTH domain family protein 3 | 2 |

## Slide 18
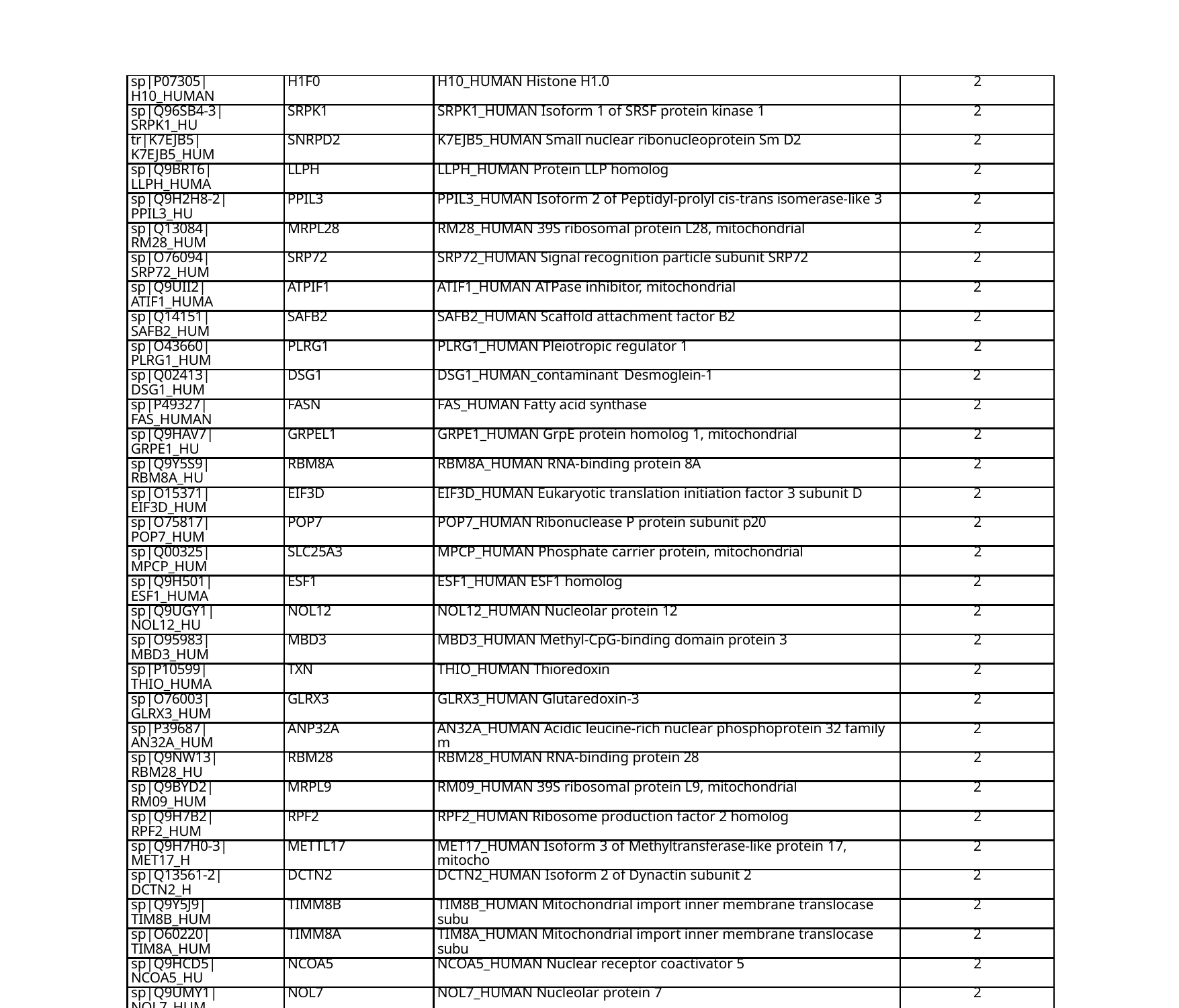

| sp|P07305|H10\_HUMAN | H1F0 | H10\_HUMAN Histone H1.0 | 2 |
| --- | --- | --- | --- |
| sp|Q96SB4-3|SRPK1\_HU | SRPK1 | SRPK1\_HUMAN Isoform 1 of SRSF protein kinase 1 | 2 |
| tr|K7EJB5|K7EJB5\_HUM | SNRPD2 | K7EJB5\_HUMAN Small nuclear ribonucleoprotein Sm D2 | 2 |
| sp|Q9BRT6|LLPH\_HUMA | LLPH | LLPH\_HUMAN Protein LLP homolog | 2 |
| sp|Q9H2H8-2|PPIL3\_HU | PPIL3 | PPIL3\_HUMAN Isoform 2 of Peptidyl-prolyl cis-trans isomerase-like 3 | 2 |
| sp|Q13084|RM28\_HUM | MRPL28 | RM28\_HUMAN 39S ribosomal protein L28, mitochondrial | 2 |
| sp|O76094|SRP72\_HUM | SRP72 | SRP72\_HUMAN Signal recognition particle subunit SRP72 | 2 |
| sp|Q9UII2|ATIF1\_HUMA | ATPIF1 | ATIF1\_HUMAN ATPase inhibitor, mitochondrial | 2 |
| sp|Q14151|SAFB2\_HUM | SAFB2 | SAFB2\_HUMAN Scaffold attachment factor B2 | 2 |
| sp|O43660|PLRG1\_HUM | PLRG1 | PLRG1\_HUMAN Pleiotropic regulator 1 | 2 |
| sp|Q02413|DSG1\_HUM | DSG1 | DSG1\_HUMAN\_contaminant Desmoglein-1 | 2 |
| sp|P49327|FAS\_HUMAN | FASN | FAS\_HUMAN Fatty acid synthase | 2 |
| sp|Q9HAV7|GRPE1\_HU | GRPEL1 | GRPE1\_HUMAN GrpE protein homolog 1, mitochondrial | 2 |
| sp|Q9Y5S9|RBM8A\_HU | RBM8A | RBM8A\_HUMAN RNA-binding protein 8A | 2 |
| sp|O15371|EIF3D\_HUM | EIF3D | EIF3D\_HUMAN Eukaryotic translation initiation factor 3 subunit D | 2 |
| sp|O75817|POP7\_HUM | POP7 | POP7\_HUMAN Ribonuclease P protein subunit p20 | 2 |
| sp|Q00325|MPCP\_HUM | SLC25A3 | MPCP\_HUMAN Phosphate carrier protein, mitochondrial | 2 |
| sp|Q9H501|ESF1\_HUMA | ESF1 | ESF1\_HUMAN ESF1 homolog | 2 |
| sp|Q9UGY1|NOL12\_HU | NOL12 | NOL12\_HUMAN Nucleolar protein 12 | 2 |
| sp|O95983|MBD3\_HUM | MBD3 | MBD3\_HUMAN Methyl-CpG-binding domain protein 3 | 2 |
| sp|P10599|THIO\_HUMA | TXN | THIO\_HUMAN Thioredoxin | 2 |
| sp|O76003|GLRX3\_HUM | GLRX3 | GLRX3\_HUMAN Glutaredoxin-3 | 2 |
| sp|P39687|AN32A\_HUM | ANP32A | AN32A\_HUMAN Acidic leucine-rich nuclear phosphoprotein 32 family m | 2 |
| sp|Q9NW13|RBM28\_HU | RBM28 | RBM28\_HUMAN RNA-binding protein 28 | 2 |
| sp|Q9BYD2|RM09\_HUM | MRPL9 | RM09\_HUMAN 39S ribosomal protein L9, mitochondrial | 2 |
| sp|Q9H7B2|RPF2\_HUM | RPF2 | RPF2\_HUMAN Ribosome production factor 2 homolog | 2 |
| sp|Q9H7H0-3|MET17\_H | METTL17 | MET17\_HUMAN Isoform 3 of Methyltransferase-like protein 17, mitocho | 2 |
| sp|Q13561-2|DCTN2\_H | DCTN2 | DCTN2\_HUMAN Isoform 2 of Dynactin subunit 2 | 2 |
| sp|Q9Y5J9|TIM8B\_HUM | TIMM8B | TIM8B\_HUMAN Mitochondrial import inner membrane translocase subu | 2 |
| sp|O60220|TIM8A\_HUM | TIMM8A | TIM8A\_HUMAN Mitochondrial import inner membrane translocase subu | 2 |
| sp|Q9HCD5|NCOA5\_HU | NCOA5 | NCOA5\_HUMAN Nuclear receptor coactivator 5 | 2 |
| sp|Q9UMY1|NOL7\_HUM | NOL7 | NOL7\_HUMAN Nucleolar protein 7 | 2 |
| sp|Q6P5R6|RL22L\_HUM | RPL22L1 | RL22L\_HUMAN 60S ribosomal protein L22-like 1 | 2 |
| sp|P30101|PDIA3\_HUM | PDIA3 | PDIA3\_HUMAN Protein disulfide-isomerase A3 | 2 |
| sp|Q9BYD3|RM04\_HUM | MRPL4 | RM04\_HUMAN 39S ribosomal protein L4, mitochondrial | 2 |

## Slide 19
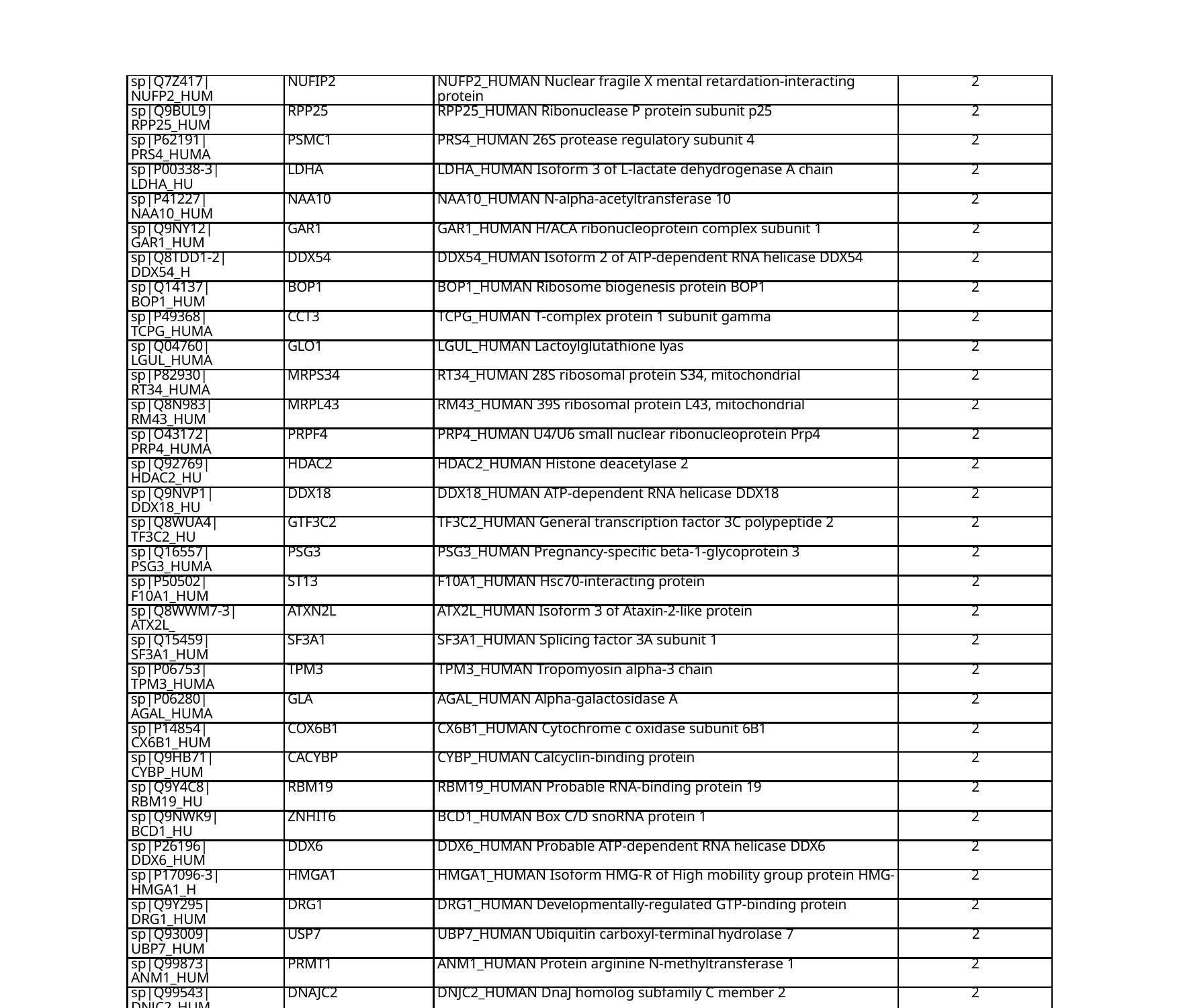

| sp|Q7Z417|NUFP2\_HUM | NUFIP2 | NUFP2\_HUMAN Nuclear fragile X mental retardation-interacting protein | 2 |
| --- | --- | --- | --- |
| sp|Q9BUL9|RPP25\_HUM | RPP25 | RPP25\_HUMAN Ribonuclease P protein subunit p25 | 2 |
| sp|P62191|PRS4\_HUMA | PSMC1 | PRS4\_HUMAN 26S protease regulatory subunit 4 | 2 |
| sp|P00338-3|LDHA\_HU | LDHA | LDHA\_HUMAN Isoform 3 of L-lactate dehydrogenase A chain | 2 |
| sp|P41227|NAA10\_HUM | NAA10 | NAA10\_HUMAN N-alpha-acetyltransferase 10 | 2 |
| sp|Q9NY12|GAR1\_HUM | GAR1 | GAR1\_HUMAN H/ACA ribonucleoprotein complex subunit 1 | 2 |
| sp|Q8TDD1-2|DDX54\_H | DDX54 | DDX54\_HUMAN Isoform 2 of ATP-dependent RNA helicase DDX54 | 2 |
| sp|Q14137|BOP1\_HUM | BOP1 | BOP1\_HUMAN Ribosome biogenesis protein BOP1 | 2 |
| sp|P49368|TCPG\_HUMA | CCT3 | TCPG\_HUMAN T-complex protein 1 subunit gamma | 2 |
| sp|Q04760|LGUL\_HUMA | GLO1 | LGUL\_HUMAN Lactoylglutathione lyas | 2 |
| sp|P82930|RT34\_HUMA | MRPS34 | RT34\_HUMAN 28S ribosomal protein S34, mitochondrial | 2 |
| sp|Q8N983|RM43\_HUM | MRPL43 | RM43\_HUMAN 39S ribosomal protein L43, mitochondrial | 2 |
| sp|O43172|PRP4\_HUMA | PRPF4 | PRP4\_HUMAN U4/U6 small nuclear ribonucleoprotein Prp4 | 2 |
| sp|Q92769|HDAC2\_HU | HDAC2 | HDAC2\_HUMAN Histone deacetylase 2 | 2 |
| sp|Q9NVP1|DDX18\_HU | DDX18 | DDX18\_HUMAN ATP-dependent RNA helicase DDX18 | 2 |
| sp|Q8WUA4|TF3C2\_HU | GTF3C2 | TF3C2\_HUMAN General transcription factor 3C polypeptide 2 | 2 |
| sp|Q16557|PSG3\_HUMA | PSG3 | PSG3\_HUMAN Pregnancy-specific beta-1-glycoprotein 3 | 2 |
| sp|P50502|F10A1\_HUM | ST13 | F10A1\_HUMAN Hsc70-interacting protein | 2 |
| sp|Q8WWM7-3|ATX2L\_ | ATXN2L | ATX2L\_HUMAN Isoform 3 of Ataxin-2-like protein | 2 |
| sp|Q15459|SF3A1\_HUM | SF3A1 | SF3A1\_HUMAN Splicing factor 3A subunit 1 | 2 |
| sp|P06753|TPM3\_HUMA | TPM3 | TPM3\_HUMAN Tropomyosin alpha-3 chain | 2 |
| sp|P06280|AGAL\_HUMA | GLA | AGAL\_HUMAN Alpha-galactosidase A | 2 |
| sp|P14854|CX6B1\_HUM | COX6B1 | CX6B1\_HUMAN Cytochrome c oxidase subunit 6B1 | 2 |
| sp|Q9HB71|CYBP\_HUM | CACYBP | CYBP\_HUMAN Calcyclin-binding protein | 2 |
| sp|Q9Y4C8|RBM19\_HU | RBM19 | RBM19\_HUMAN Probable RNA-binding protein 19 | 2 |
| sp|Q9NWK9|BCD1\_HU | ZNHIT6 | BCD1\_HUMAN Box C/D snoRNA protein 1 | 2 |
| sp|P26196|DDX6\_HUM | DDX6 | DDX6\_HUMAN Probable ATP-dependent RNA helicase DDX6 | 2 |
| sp|P17096-3|HMGA1\_H | HMGA1 | HMGA1\_HUMAN Isoform HMG-R of High mobility group protein HMG- | 2 |
| sp|Q9Y295|DRG1\_HUM | DRG1 | DRG1\_HUMAN Developmentally-regulated GTP-binding protein | 2 |
| sp|Q93009|UBP7\_HUM | USP7 | UBP7\_HUMAN Ubiquitin carboxyl-terminal hydrolase 7 | 2 |
| sp|Q99873|ANM1\_HUM | PRMT1 | ANM1\_HUMAN Protein arginine N-methyltransferase 1 | 2 |
| sp|Q99543|DNJC2\_HUM | DNAJC2 | DNJC2\_HUMAN DnaJ homolog subfamily C member 2 | 2 |
| sp|P98179|RBM3\_HUM | RBM3 | RBM3\_HUMAN Putative RNA-binding protein 3 | 2 |
| sp|Q9NQ50|RM40\_HUM | MRPL40 | RM40\_HUMAN 39S ribosomal protein L40, mitochondrial | 2 |
| sp|P24752|THIL\_HUMA | ACAT1 | THIL\_HUMAN Acetyl-CoA acetyltransferase, mitochondria | 2 |

## Slide 20
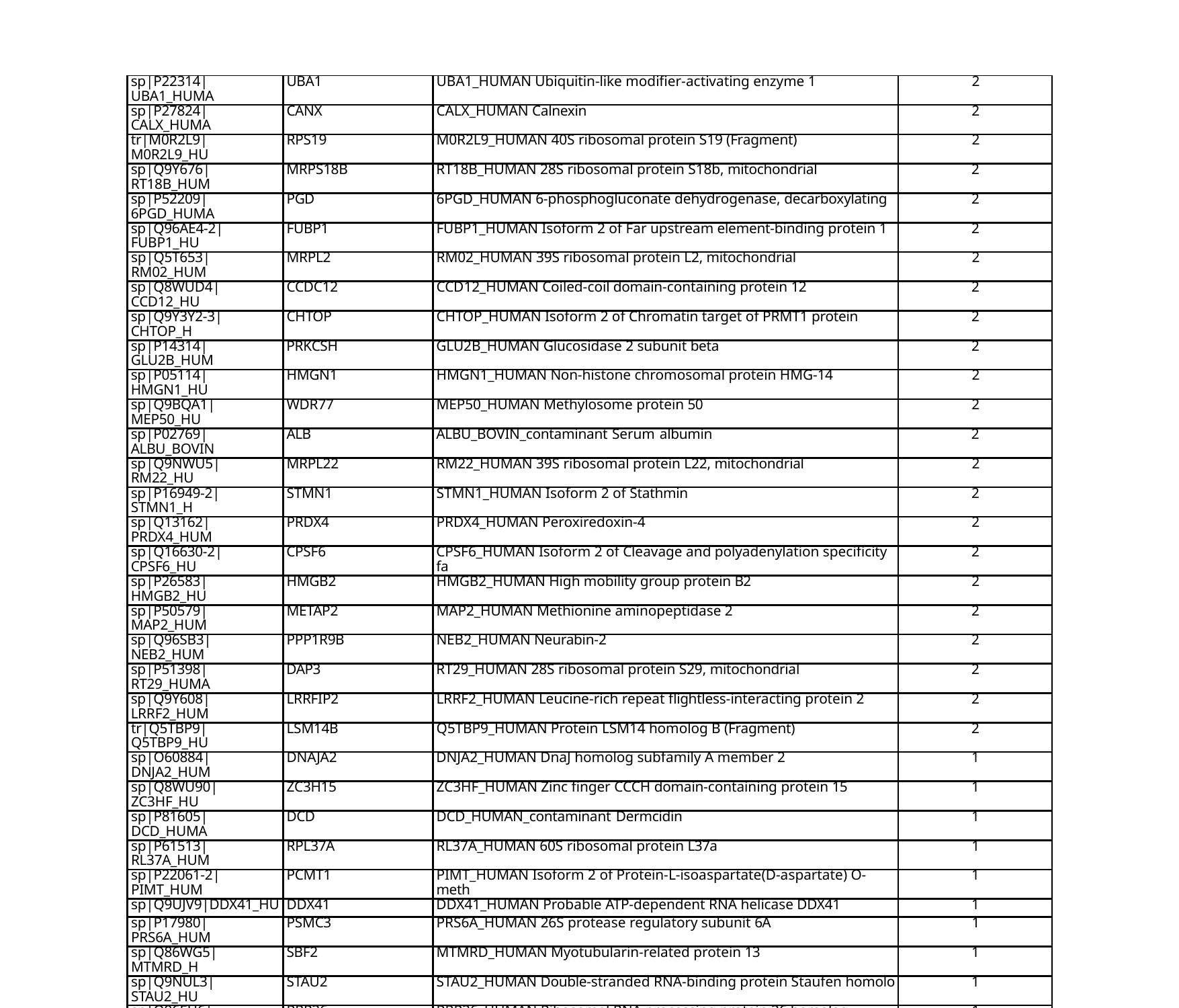

| sp|P22314|UBA1\_HUMA | UBA1 | UBA1\_HUMAN Ubiquitin-like modifier-activating enzyme 1 | 2 |
| --- | --- | --- | --- |
| sp|P27824|CALX\_HUMA | CANX | CALX\_HUMAN Calnexin | 2 |
| tr|M0R2L9|M0R2L9\_HU | RPS19 | M0R2L9\_HUMAN 40S ribosomal protein S19 (Fragment) | 2 |
| sp|Q9Y676|RT18B\_HUM | MRPS18B | RT18B\_HUMAN 28S ribosomal protein S18b, mitochondrial | 2 |
| sp|P52209|6PGD\_HUMA | PGD | 6PGD\_HUMAN 6-phosphogluconate dehydrogenase, decarboxylating | 2 |
| sp|Q96AE4-2|FUBP1\_HU | FUBP1 | FUBP1\_HUMAN Isoform 2 of Far upstream element-binding protein 1 | 2 |
| sp|Q5T653|RM02\_HUM | MRPL2 | RM02\_HUMAN 39S ribosomal protein L2, mitochondrial | 2 |
| sp|Q8WUD4|CCD12\_HU | CCDC12 | CCD12\_HUMAN Coiled-coil domain-containing protein 12 | 2 |
| sp|Q9Y3Y2-3|CHTOP\_H | CHTOP | CHTOP\_HUMAN Isoform 2 of Chromatin target of PRMT1 protein | 2 |
| sp|P14314|GLU2B\_HUM | PRKCSH | GLU2B\_HUMAN Glucosidase 2 subunit beta | 2 |
| sp|P05114|HMGN1\_HU | HMGN1 | HMGN1\_HUMAN Non-histone chromosomal protein HMG-14 | 2 |
| sp|Q9BQA1|MEP50\_HU | WDR77 | MEP50\_HUMAN Methylosome protein 50 | 2 |
| sp|P02769|ALBU\_BOVIN | ALB | ALBU\_BOVIN\_contaminant Serum albumin | 2 |
| sp|Q9NWU5|RM22\_HU | MRPL22 | RM22\_HUMAN 39S ribosomal protein L22, mitochondrial | 2 |
| sp|P16949-2|STMN1\_H | STMN1 | STMN1\_HUMAN Isoform 2 of Stathmin | 2 |
| sp|Q13162|PRDX4\_HUM | PRDX4 | PRDX4\_HUMAN Peroxiredoxin-4 | 2 |
| sp|Q16630-2|CPSF6\_HU | CPSF6 | CPSF6\_HUMAN Isoform 2 of Cleavage and polyadenylation specificity fa | 2 |
| sp|P26583|HMGB2\_HU | HMGB2 | HMGB2\_HUMAN High mobility group protein B2 | 2 |
| sp|P50579|MAP2\_HUM | METAP2 | MAP2\_HUMAN Methionine aminopeptidase 2 | 2 |
| sp|Q96SB3|NEB2\_HUM | PPP1R9B | NEB2\_HUMAN Neurabin-2 | 2 |
| sp|P51398|RT29\_HUMA | DAP3 | RT29\_HUMAN 28S ribosomal protein S29, mitochondrial | 2 |
| sp|Q9Y608|LRRF2\_HUM | LRRFIP2 | LRRF2\_HUMAN Leucine-rich repeat flightless-interacting protein 2 | 2 |
| tr|Q5TBP9|Q5TBP9\_HU | LSM14B | Q5TBP9\_HUMAN Protein LSM14 homolog B (Fragment) | 2 |
| sp|O60884|DNJA2\_HUM | DNAJA2 | DNJA2\_HUMAN DnaJ homolog subfamily A member 2 | 1 |
| sp|Q8WU90|ZC3HF\_HU | ZC3H15 | ZC3HF\_HUMAN Zinc finger CCCH domain-containing protein 15 | 1 |
| sp|P81605|DCD\_HUMA | DCD | DCD\_HUMAN\_contaminant Dermcidin | 1 |
| sp|P61513|RL37A\_HUM | RPL37A | RL37A\_HUMAN 60S ribosomal protein L37a | 1 |
| sp|P22061-2|PIMT\_HUM | PCMT1 | PIMT\_HUMAN Isoform 2 of Protein-L-isoaspartate(D-aspartate) O-meth | 1 |
| sp|Q9UJV9|DDX41\_HU | DDX41 | DDX41\_HUMAN Probable ATP-dependent RNA helicase DDX41 | 1 |
| sp|P17980|PRS6A\_HUM | PSMC3 | PRS6A\_HUMAN 26S protease regulatory subunit 6A | 1 |
| sp|Q86WG5|MTMRD\_H | SBF2 | MTMRD\_HUMAN Myotubularin-related protein 13 | 1 |
| sp|Q9NUL3|STAU2\_HU | STAU2 | STAU2\_HUMAN Double-stranded RNA-binding protein Staufen homolo | 1 |
| sp|Q96EU6|RRP36\_HUM | RRP36 | RRP36\_HUMAN Ribosomal RNA processing protein 36 homolog | 1 |
| sp|O75475|PSIP1\_HUM | PSIP1 | PSIP1\_HUMAN PC4 and SFRS1-interacting protein | 1 |
| sp|Q03701|CEBPZ\_HUM | CEBPZ | CEBPZ\_HUMAN CCAAT/enhancer-binding protein zeta | 1 |

## Slide 21
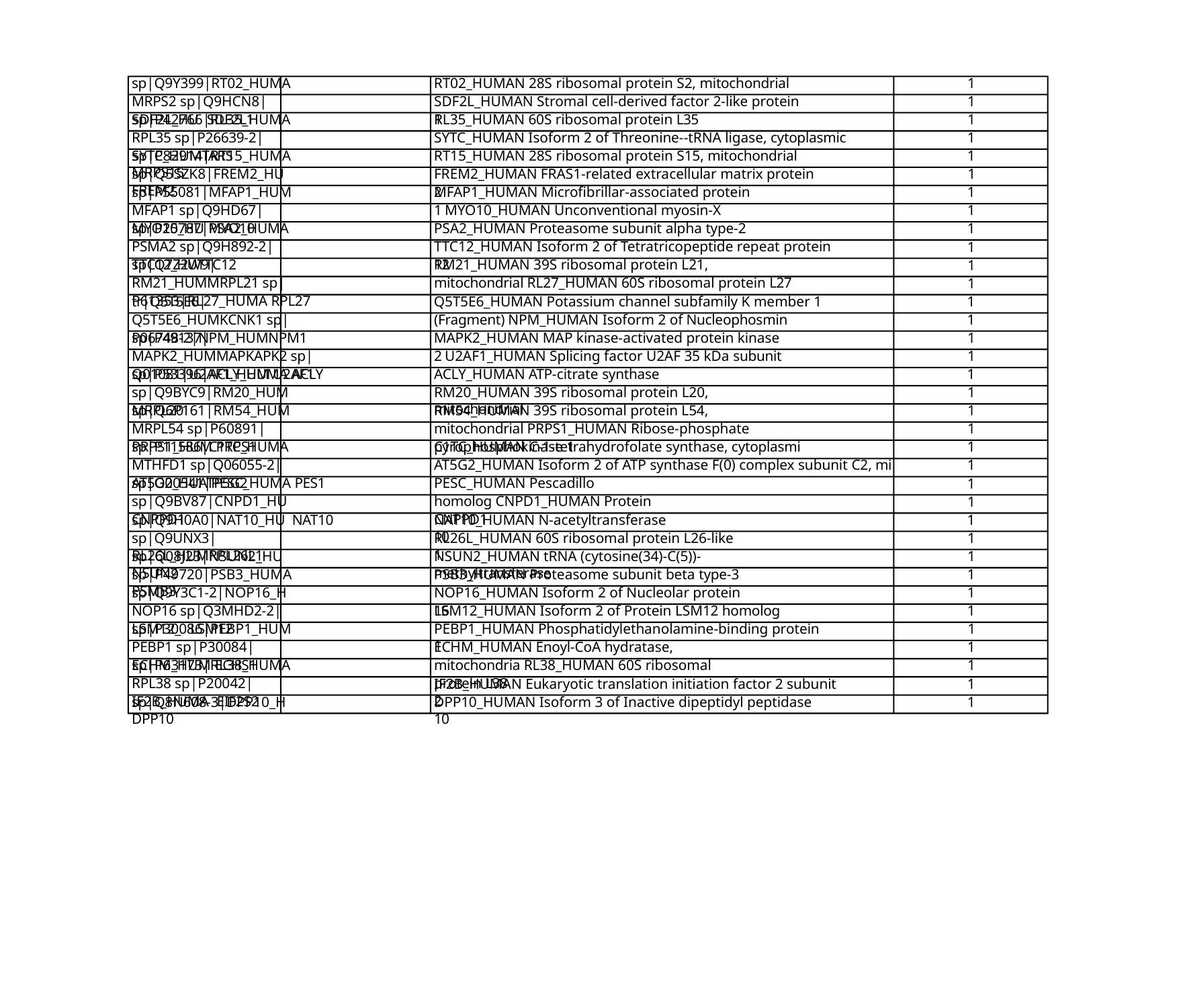

sp|Q9Y399|RT02_HUMA MRPS2 sp|Q9HCN8|SDF2L_HU SDF2L1
RT02_HUMAN 28S ribosomal protein S2, mitochondrial SDF2L_HUMAN Stromal cell-derived factor 2-like protein 1
1
1
sp|P42766|RL35_HUMA RPL35 sp|P26639-2|SYTC_HUMTARS
RL35_HUMAN 60S ribosomal protein L35
SYTC_HUMAN Isoform 2 of Threonine--tRNA ligase, cytoplasmic
1
1
RT15_HUMAN 28S ribosomal protein S15, mitochondrial FREM2_HUMAN FRAS1-related extracellular matrix protein 2
1
1
sp|P82914|RT15_HUMA MRPS15
sp|Q5SZK8|FREM2_HU FREM2
sp|P55081|MFAP1_HUM MFAP1 sp|Q9HD67|MYO10_HU MYO10
MFAP1_HUMAN Microfibrillar-associated protein 1 MYO10_HUMAN Unconventional myosin-X
1
1
sp|P25787|PSA2_HUMA PSMA2 sp|Q9H892-2|TTC12_HUTTC12
PSA2_HUMAN Proteasome subunit alpha type-2 TTC12_HUMAN Isoform 2 of Tetratricopeptide repeat protein 12
1
1
sp|Q7Z2W9|RM21_HUMMRPL21 sp|P61353|RL27_HUMA RPL27
RM21_HUMAN 39S ribosomal protein L21, mitochondrial RL27_HUMAN 60S ribosomal protein L27
1
1
tr|Q5T5E6|Q5T5E6_HUMKCNK1 sp|P06748-2|NPM_HUMNPM1
Q5T5E6_HUMAN Potassium channel subfamily K member 1 (Fragment) NPM_HUMAN Isoform 2 of Nucleophosmin
1
1
sp|P49137|MAPK2_HUMMAPKAPK2 sp|Q01081|U2AF1_HUM U2AF1
MAPK2_HUMAN MAP kinase-activated protein kinase 2 U2AF1_HUMAN Splicing factor U2AF 35 kDa subunit
1
1
sp|P53396|ACLY_HUMA ACLY sp|Q9BYC9|RM20_HUM MRPL20
ACLY_HUMAN ATP-citrate synthase
RM20_HUMAN 39S ribosomal protein L20, mitochondrial
1
1
sp|Q6P161|RM54_HUM MRPL54 sp|P60891|PRPS1_HUM PRPS1
RM54_HUMAN 39S ribosomal protein L54, mitochondrial PRPS1_HUMAN Ribose-phosphate pyrophosphokinase 1
1
1
sp|P11586|C1TC_HUMA MTHFD1 sp|Q06055-2|AT5G2_HUATP5G2
C1TC_HUMAN C-1-tetrahydrofolate synthase, cytoplasmi AT5G2_HUMAN Isoform 2 of ATP synthase F(0) complex subunit C2, mi
1
1
sp|O00541|PESC_HUMA PES1 sp|Q9BV87|CNPD1_HU CNPPD1
PESC_HUMAN Pescadillo homolog CNPD1_HUMAN Protein CNPPD1
1
1
sp|Q9H0A0|NAT10_HU NAT10 sp|Q9UNX3|RL26L_HUMRPL26L1
1
1
NAT10_HUMAN N-acetyltransferase 10
RL26L_HUMAN 60S ribosomal protein L26-like 1
1
1
sp|Q08J23|NSUN2_HU NSUN2
NSUN2_HUMAN tRNA (cytosine(34)-C(5))-methyltransferase
PSB3_HUMAN Proteasome subunit beta type-3 NOP16_HUMAN Isoform 2 of Nucleolar protein 16
sp|P49720|PSB3_HUMA PSMB3
sp|Q9Y3C1-2|NOP16_H NOP16 sp|Q3MHD2-2|LSM12_ LSM12
1
1
LSM12_HUMAN Isoform 2 of Protein LSM12 homolog
sp|P30086|PEBP1_HUM PEBP1 sp|P30084|ECHM_HUM ECHS1
1
1
PEBP1_HUMAN Phosphatidylethanolamine-binding protein 1
ECHM_HUMAN Enoyl-CoA hydratase, mitochondria RL38_HUMAN 60S ribosomal protein L38
sp|P63173|RL38_HUMA RPL38 sp|P20042|IF2B_HUMA EIF2S2
1
1
IF2B_HUMAN Eukaryotic translation initiation factor 2 subunit 2
sp|Q8N608-3|DPP10_H DPP10
DPP10_HUMAN Isoform 3 of Inactive dipeptidyl peptidase 10
1

## Slide 22
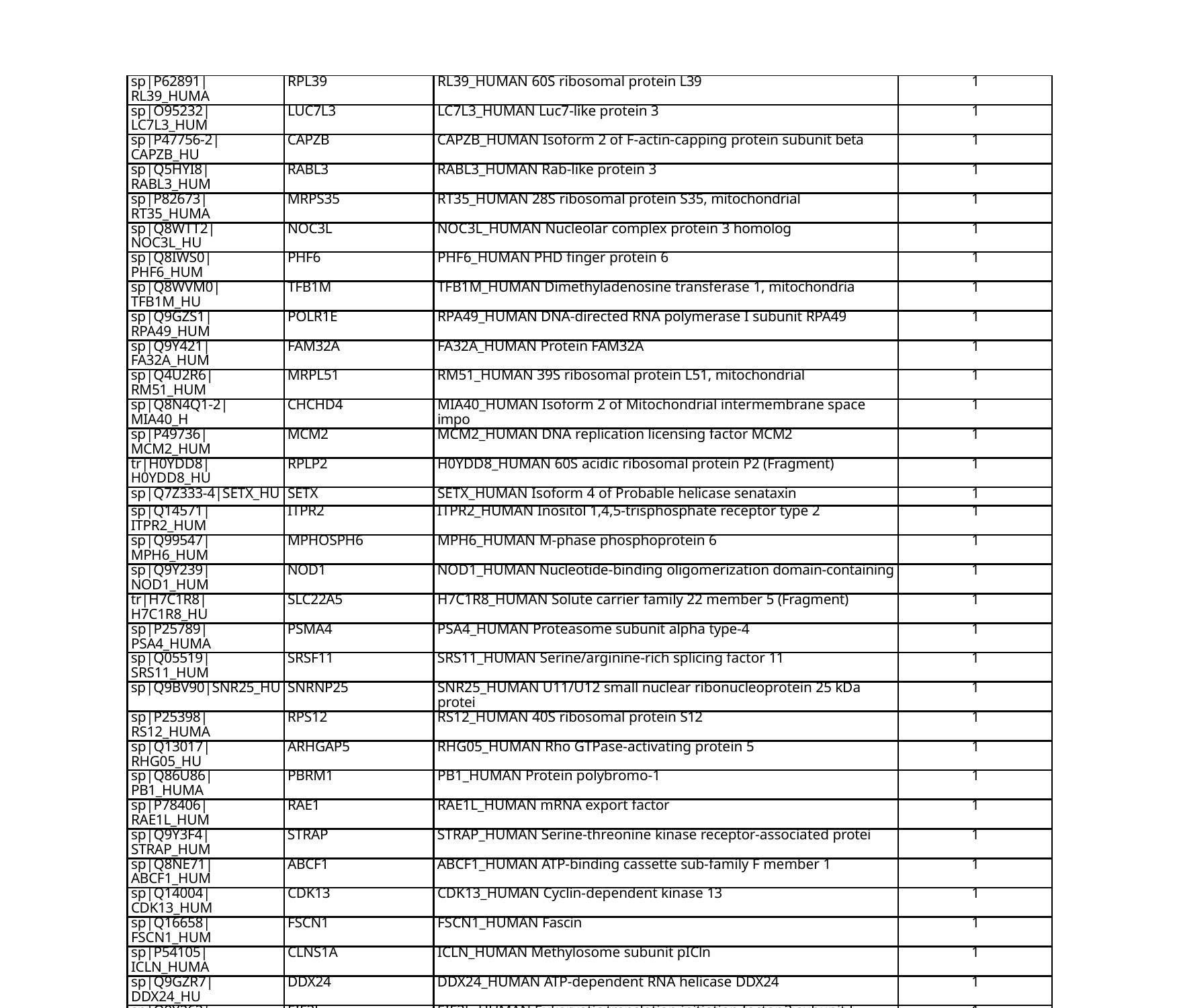

| sp|P62891|RL39\_HUMA | RPL39 | RL39\_HUMAN 60S ribosomal protein L39 | 1 |
| --- | --- | --- | --- |
| sp|O95232|LC7L3\_HUM | LUC7L3 | LC7L3\_HUMAN Luc7-like protein 3 | 1 |
| sp|P47756-2|CAPZB\_HU | CAPZB | CAPZB\_HUMAN Isoform 2 of F-actin-capping protein subunit beta | 1 |
| sp|Q5HYI8|RABL3\_HUM | RABL3 | RABL3\_HUMAN Rab-like protein 3 | 1 |
| sp|P82673|RT35\_HUMA | MRPS35 | RT35\_HUMAN 28S ribosomal protein S35, mitochondrial | 1 |
| sp|Q8WTT2|NOC3L\_HU | NOC3L | NOC3L\_HUMAN Nucleolar complex protein 3 homolog | 1 |
| sp|Q8IWS0|PHF6\_HUM | PHF6 | PHF6\_HUMAN PHD finger protein 6 | 1 |
| sp|Q8WVM0|TFB1M\_HU | TFB1M | TFB1M\_HUMAN Dimethyladenosine transferase 1, mitochondria | 1 |
| sp|Q9GZS1|RPA49\_HUM | POLR1E | RPA49\_HUMAN DNA-directed RNA polymerase I subunit RPA49 | 1 |
| sp|Q9Y421|FA32A\_HUM | FAM32A | FA32A\_HUMAN Protein FAM32A | 1 |
| sp|Q4U2R6|RM51\_HUM | MRPL51 | RM51\_HUMAN 39S ribosomal protein L51, mitochondrial | 1 |
| sp|Q8N4Q1-2|MIA40\_H | CHCHD4 | MIA40\_HUMAN Isoform 2 of Mitochondrial intermembrane space impo | 1 |
| sp|P49736|MCM2\_HUM | MCM2 | MCM2\_HUMAN DNA replication licensing factor MCM2 | 1 |
| tr|H0YDD8|H0YDD8\_HU | RPLP2 | H0YDD8\_HUMAN 60S acidic ribosomal protein P2 (Fragment) | 1 |
| sp|Q7Z333-4|SETX\_HU | SETX | SETX\_HUMAN Isoform 4 of Probable helicase senataxin | 1 |
| sp|Q14571|ITPR2\_HUM | ITPR2 | ITPR2\_HUMAN Inositol 1,4,5-trisphosphate receptor type 2 | 1 |
| sp|Q99547|MPH6\_HUM | MPHOSPH6 | MPH6\_HUMAN M-phase phosphoprotein 6 | 1 |
| sp|Q9Y239|NOD1\_HUM | NOD1 | NOD1\_HUMAN Nucleotide-binding oligomerization domain-containing | 1 |
| tr|H7C1R8|H7C1R8\_HU | SLC22A5 | H7C1R8\_HUMAN Solute carrier family 22 member 5 (Fragment) | 1 |
| sp|P25789|PSA4\_HUMA | PSMA4 | PSA4\_HUMAN Proteasome subunit alpha type-4 | 1 |
| sp|Q05519|SRS11\_HUM | SRSF11 | SRS11\_HUMAN Serine/arginine-rich splicing factor 11 | 1 |
| sp|Q9BV90|SNR25\_HU | SNRNP25 | SNR25\_HUMAN U11/U12 small nuclear ribonucleoprotein 25 kDa protei | 1 |
| sp|P25398|RS12\_HUMA | RPS12 | RS12\_HUMAN 40S ribosomal protein S12 | 1 |
| sp|Q13017|RHG05\_HU | ARHGAP5 | RHG05\_HUMAN Rho GTPase-activating protein 5 | 1 |
| sp|Q86U86|PB1\_HUMA | PBRM1 | PB1\_HUMAN Protein polybromo-1 | 1 |
| sp|P78406|RAE1L\_HUM | RAE1 | RAE1L\_HUMAN mRNA export factor | 1 |
| sp|Q9Y3F4|STRAP\_HUM | STRAP | STRAP\_HUMAN Serine-threonine kinase receptor-associated protei | 1 |
| sp|Q8NE71|ABCF1\_HUM | ABCF1 | ABCF1\_HUMAN ATP-binding cassette sub-family F member 1 | 1 |
| sp|Q14004|CDK13\_HUM | CDK13 | CDK13\_HUMAN Cyclin-dependent kinase 13 | 1 |
| sp|Q16658|FSCN1\_HUM | FSCN1 | FSCN1\_HUMAN Fascin | 1 |
| sp|P54105|ICLN\_HUMA | CLNS1A | ICLN\_HUMAN Methylosome subunit pICln | 1 |
| sp|Q9GZR7|DDX24\_HU | DDX24 | DDX24\_HUMAN ATP-dependent RNA helicase DDX24 | 1 |
| sp|Q9Y262|EIF3L\_HUMA | EIF3L | EIF3L\_HUMAN Eukaryotic translation initiation factor 3 subunit L | 1 |
| sp|Q8N100|ATOH7\_HU | ATOH7 | ATOH7\_HUMAN Protein atonal homolog 7 | 1 |
| sp|O60783|RT14\_HUMA | MRPS14 | RT14\_HUMAN 28S ribosomal protein S14, mitochondrial | 1 |

## Slide 23
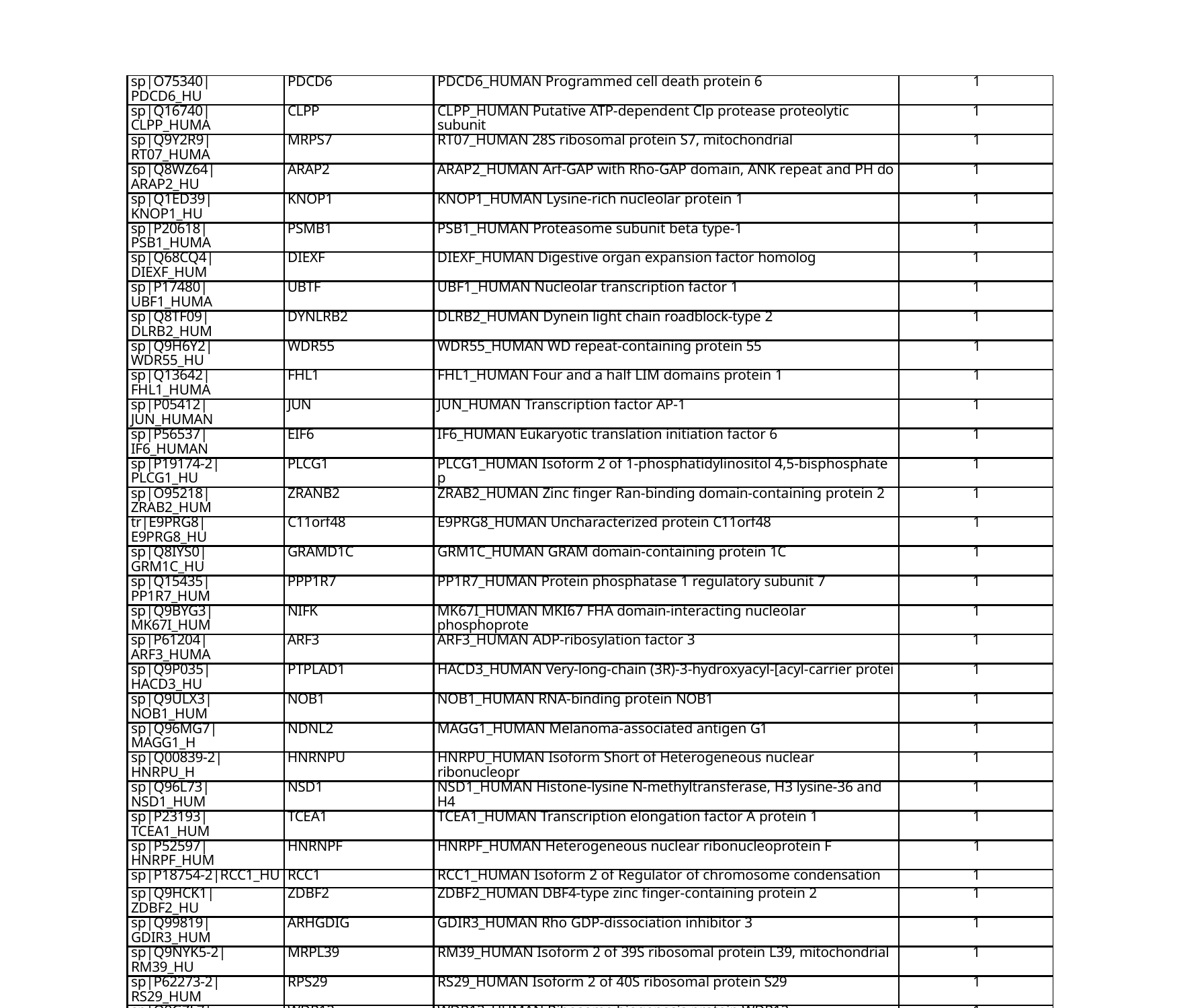

| sp|O75340|PDCD6\_HU | PDCD6 | PDCD6\_HUMAN Programmed cell death protein 6 | 1 |
| --- | --- | --- | --- |
| sp|Q16740|CLPP\_HUMA | CLPP | CLPP\_HUMAN Putative ATP-dependent Clp protease proteolytic subunit | 1 |
| sp|Q9Y2R9|RT07\_HUMA | MRPS7 | RT07\_HUMAN 28S ribosomal protein S7, mitochondrial | 1 |
| sp|Q8WZ64|ARAP2\_HU | ARAP2 | ARAP2\_HUMAN Arf-GAP with Rho-GAP domain, ANK repeat and PH do | 1 |
| sp|Q1ED39|KNOP1\_HU | KNOP1 | KNOP1\_HUMAN Lysine-rich nucleolar protein 1 | 1 |
| sp|P20618|PSB1\_HUMA | PSMB1 | PSB1\_HUMAN Proteasome subunit beta type-1 | 1 |
| sp|Q68CQ4|DIEXF\_HUM | DIEXF | DIEXF\_HUMAN Digestive organ expansion factor homolog | 1 |
| sp|P17480|UBF1\_HUMA | UBTF | UBF1\_HUMAN Nucleolar transcription factor 1 | 1 |
| sp|Q8TF09|DLRB2\_HUM | DYNLRB2 | DLRB2\_HUMAN Dynein light chain roadblock-type 2 | 1 |
| sp|Q9H6Y2|WDR55\_HU | WDR55 | WDR55\_HUMAN WD repeat-containing protein 55 | 1 |
| sp|Q13642|FHL1\_HUMA | FHL1 | FHL1\_HUMAN Four and a half LIM domains protein 1 | 1 |
| sp|P05412|JUN\_HUMAN | JUN | JUN\_HUMAN Transcription factor AP-1 | 1 |
| sp|P56537|IF6\_HUMAN | EIF6 | IF6\_HUMAN Eukaryotic translation initiation factor 6 | 1 |
| sp|P19174-2|PLCG1\_HU | PLCG1 | PLCG1\_HUMAN Isoform 2 of 1-phosphatidylinositol 4,5-bisphosphate p | 1 |
| sp|O95218|ZRAB2\_HUM | ZRANB2 | ZRAB2\_HUMAN Zinc finger Ran-binding domain-containing protein 2 | 1 |
| tr|E9PRG8|E9PRG8\_HU | C11orf48 | E9PRG8\_HUMAN Uncharacterized protein C11orf48 | 1 |
| sp|Q8IYS0|GRM1C\_HU | GRAMD1C | GRM1C\_HUMAN GRAM domain-containing protein 1C | 1 |
| sp|Q15435|PP1R7\_HUM | PPP1R7 | PP1R7\_HUMAN Protein phosphatase 1 regulatory subunit 7 | 1 |
| sp|Q9BYG3|MK67I\_HUM | NIFK | MK67I\_HUMAN MKI67 FHA domain-interacting nucleolar phosphoprote | 1 |
| sp|P61204|ARF3\_HUMA | ARF3 | ARF3\_HUMAN ADP-ribosylation factor 3 | 1 |
| sp|Q9P035|HACD3\_HU | PTPLAD1 | HACD3\_HUMAN Very-long-chain (3R)-3-hydroxyacyl-[acyl-carrier protei | 1 |
| sp|Q9ULX3|NOB1\_HUM | NOB1 | NOB1\_HUMAN RNA-binding protein NOB1 | 1 |
| sp|Q96MG7|MAGG1\_H | NDNL2 | MAGG1\_HUMAN Melanoma-associated antigen G1 | 1 |
| sp|Q00839-2|HNRPU\_H | HNRNPU | HNRPU\_HUMAN Isoform Short of Heterogeneous nuclear ribonucleopr | 1 |
| sp|Q96L73|NSD1\_HUM | NSD1 | NSD1\_HUMAN Histone-lysine N-methyltransferase, H3 lysine-36 and H4 | 1 |
| sp|P23193|TCEA1\_HUM | TCEA1 | TCEA1\_HUMAN Transcription elongation factor A protein 1 | 1 |
| sp|P52597|HNRPF\_HUM | HNRNPF | HNRPF\_HUMAN Heterogeneous nuclear ribonucleoprotein F | 1 |
| sp|P18754-2|RCC1\_HU | RCC1 | RCC1\_HUMAN Isoform 2 of Regulator of chromosome condensation | 1 |
| sp|Q9HCK1|ZDBF2\_HU | ZDBF2 | ZDBF2\_HUMAN DBF4-type zinc finger-containing protein 2 | 1 |
| sp|Q99819|GDIR3\_HUM | ARHGDIG | GDIR3\_HUMAN Rho GDP-dissociation inhibitor 3 | 1 |
| sp|Q9NYK5-2|RM39\_HU | MRPL39 | RM39\_HUMAN Isoform 2 of 39S ribosomal protein L39, mitochondrial | 1 |
| sp|P62273-2|RS29\_HUM | RPS29 | RS29\_HUMAN Isoform 2 of 40S ribosomal protein S29 | 1 |
| sp|Q9GZL7|WDR12\_HU | WDR12 | WDR12\_HUMAN Ribosome biogenesis protein WDR12 | 1 |
| sp|P10515|ODP2\_HUM | DLAT | ODP2\_HUMAN Dihydrolipoyllysine-residue acetyltransferase component | 1 |
| sp|Q9Y5L4|TIM13\_HUM | TIMM13 | TIM13\_HUMAN Mitochondrial import inner membrane translocase subu | 1 |

## Slide 24
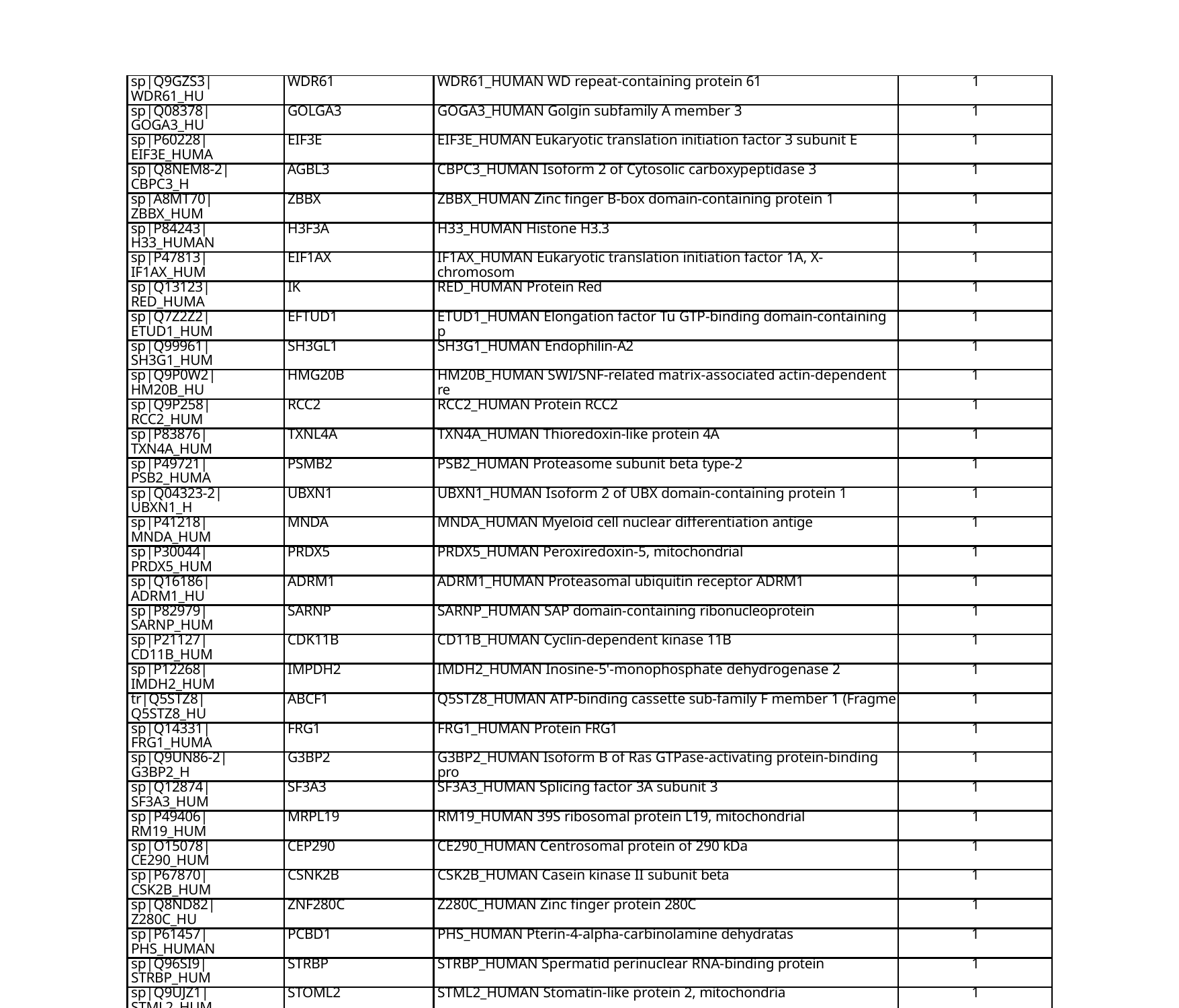

| sp|Q9GZS3|WDR61\_HU | WDR61 | WDR61\_HUMAN WD repeat-containing protein 61 | 1 |
| --- | --- | --- | --- |
| sp|Q08378|GOGA3\_HU | GOLGA3 | GOGA3\_HUMAN Golgin subfamily A member 3 | 1 |
| sp|P60228|EIF3E\_HUMA | EIF3E | EIF3E\_HUMAN Eukaryotic translation initiation factor 3 subunit E | 1 |
| sp|Q8NEM8-2|CBPC3\_H | AGBL3 | CBPC3\_HUMAN Isoform 2 of Cytosolic carboxypeptidase 3 | 1 |
| sp|A8MT70|ZBBX\_HUM | ZBBX | ZBBX\_HUMAN Zinc finger B-box domain-containing protein 1 | 1 |
| sp|P84243|H33\_HUMAN | H3F3A | H33\_HUMAN Histone H3.3 | 1 |
| sp|P47813|IF1AX\_HUM | EIF1AX | IF1AX\_HUMAN Eukaryotic translation initiation factor 1A, X-chromosom | 1 |
| sp|Q13123|RED\_HUMA | IK | RED\_HUMAN Protein Red | 1 |
| sp|Q7Z2Z2|ETUD1\_HUM | EFTUD1 | ETUD1\_HUMAN Elongation factor Tu GTP-binding domain-containing p | 1 |
| sp|Q99961|SH3G1\_HUM | SH3GL1 | SH3G1\_HUMAN Endophilin-A2 | 1 |
| sp|Q9P0W2|HM20B\_HU | HMG20B | HM20B\_HUMAN SWI/SNF-related matrix-associated actin-dependent re | 1 |
| sp|Q9P258|RCC2\_HUM | RCC2 | RCC2\_HUMAN Protein RCC2 | 1 |
| sp|P83876|TXN4A\_HUM | TXNL4A | TXN4A\_HUMAN Thioredoxin-like protein 4A | 1 |
| sp|P49721|PSB2\_HUMA | PSMB2 | PSB2\_HUMAN Proteasome subunit beta type-2 | 1 |
| sp|Q04323-2|UBXN1\_H | UBXN1 | UBXN1\_HUMAN Isoform 2 of UBX domain-containing protein 1 | 1 |
| sp|P41218|MNDA\_HUM | MNDA | MNDA\_HUMAN Myeloid cell nuclear differentiation antige | 1 |
| sp|P30044|PRDX5\_HUM | PRDX5 | PRDX5\_HUMAN Peroxiredoxin-5, mitochondrial | 1 |
| sp|Q16186|ADRM1\_HU | ADRM1 | ADRM1\_HUMAN Proteasomal ubiquitin receptor ADRM1 | 1 |
| sp|P82979|SARNP\_HUM | SARNP | SARNP\_HUMAN SAP domain-containing ribonucleoprotein | 1 |
| sp|P21127|CD11B\_HUM | CDK11B | CD11B\_HUMAN Cyclin-dependent kinase 11B | 1 |
| sp|P12268|IMDH2\_HUM | IMPDH2 | IMDH2\_HUMAN Inosine-5'-monophosphate dehydrogenase 2 | 1 |
| tr|Q5STZ8|Q5STZ8\_HU | ABCF1 | Q5STZ8\_HUMAN ATP-binding cassette sub-family F member 1 (Fragme | 1 |
| sp|Q14331|FRG1\_HUMA | FRG1 | FRG1\_HUMAN Protein FRG1 | 1 |
| sp|Q9UN86-2|G3BP2\_H | G3BP2 | G3BP2\_HUMAN Isoform B of Ras GTPase-activating protein-binding pro | 1 |
| sp|Q12874|SF3A3\_HUM | SF3A3 | SF3A3\_HUMAN Splicing factor 3A subunit 3 | 1 |
| sp|P49406|RM19\_HUM | MRPL19 | RM19\_HUMAN 39S ribosomal protein L19, mitochondrial | 1 |
| sp|O15078|CE290\_HUM | CEP290 | CE290\_HUMAN Centrosomal protein of 290 kDa | 1 |
| sp|P67870|CSK2B\_HUM | CSNK2B | CSK2B\_HUMAN Casein kinase II subunit beta | 1 |
| sp|Q8ND82|Z280C\_HU | ZNF280C | Z280C\_HUMAN Zinc finger protein 280C | 1 |
| sp|P61457|PHS\_HUMAN | PCBD1 | PHS\_HUMAN Pterin-4-alpha-carbinolamine dehydratas | 1 |
| sp|Q96SI9|STRBP\_HUM | STRBP | STRBP\_HUMAN Spermatid perinuclear RNA-binding protein | 1 |
| sp|Q9UJZ1|STML2\_HUM | STOML2 | STML2\_HUMAN Stomatin-like protein 2, mitochondria | 1 |
| sp|Q9NRX4|PHP14\_HU | PHPT1 | PHP14\_HUMAN 14 kDa phosphohistidine phosphatase | 1 |
| sp|Q15572|TAF1C\_HUM | TAF1C | TAF1C\_HUMAN TATA box-binding protein-associated factor RNA polym | 1 |
| sp|P62158|CALM\_HUM | CALM1 | CALM\_HUMAN Calmodulin | 1 |

## Slide 25
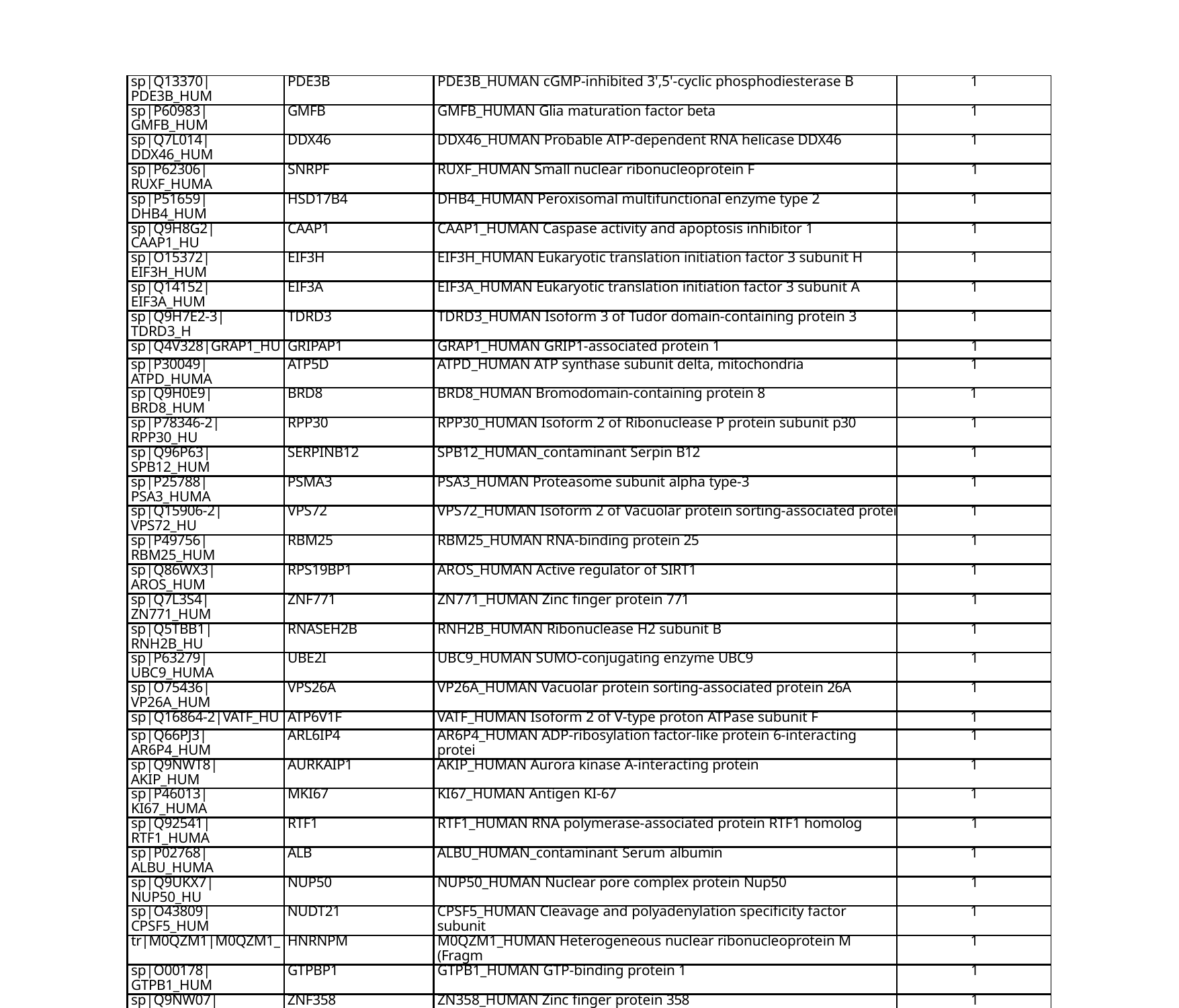

| sp|Q13370|PDE3B\_HUM | PDE3B | PDE3B\_HUMAN cGMP-inhibited 3',5'-cyclic phosphodiesterase B | 1 |
| --- | --- | --- | --- |
| sp|P60983|GMFB\_HUM | GMFB | GMFB\_HUMAN Glia maturation factor beta | 1 |
| sp|Q7L014|DDX46\_HUM | DDX46 | DDX46\_HUMAN Probable ATP-dependent RNA helicase DDX46 | 1 |
| sp|P62306|RUXF\_HUMA | SNRPF | RUXF\_HUMAN Small nuclear ribonucleoprotein F | 1 |
| sp|P51659|DHB4\_HUM | HSD17B4 | DHB4\_HUMAN Peroxisomal multifunctional enzyme type 2 | 1 |
| sp|Q9H8G2|CAAP1\_HU | CAAP1 | CAAP1\_HUMAN Caspase activity and apoptosis inhibitor 1 | 1 |
| sp|O15372|EIF3H\_HUM | EIF3H | EIF3H\_HUMAN Eukaryotic translation initiation factor 3 subunit H | 1 |
| sp|Q14152|EIF3A\_HUM | EIF3A | EIF3A\_HUMAN Eukaryotic translation initiation factor 3 subunit A | 1 |
| sp|Q9H7E2-3|TDRD3\_H | TDRD3 | TDRD3\_HUMAN Isoform 3 of Tudor domain-containing protein 3 | 1 |
| sp|Q4V328|GRAP1\_HU | GRIPAP1 | GRAP1\_HUMAN GRIP1-associated protein 1 | 1 |
| sp|P30049|ATPD\_HUMA | ATP5D | ATPD\_HUMAN ATP synthase subunit delta, mitochondria | 1 |
| sp|Q9H0E9|BRD8\_HUM | BRD8 | BRD8\_HUMAN Bromodomain-containing protein 8 | 1 |
| sp|P78346-2|RPP30\_HU | RPP30 | RPP30\_HUMAN Isoform 2 of Ribonuclease P protein subunit p30 | 1 |
| sp|Q96P63|SPB12\_HUM | SERPINB12 | SPB12\_HUMAN\_contaminant Serpin B12 | 1 |
| sp|P25788|PSA3\_HUMA | PSMA3 | PSA3\_HUMAN Proteasome subunit alpha type-3 | 1 |
| sp|Q15906-2|VPS72\_HU | VPS72 | VPS72\_HUMAN Isoform 2 of Vacuolar protein sorting-associated protei | 1 |
| sp|P49756|RBM25\_HUM | RBM25 | RBM25\_HUMAN RNA-binding protein 25 | 1 |
| sp|Q86WX3|AROS\_HUM | RPS19BP1 | AROS\_HUMAN Active regulator of SIRT1 | 1 |
| sp|Q7L3S4|ZN771\_HUM | ZNF771 | ZN771\_HUMAN Zinc finger protein 771 | 1 |
| sp|Q5TBB1|RNH2B\_HU | RNASEH2B | RNH2B\_HUMAN Ribonuclease H2 subunit B | 1 |
| sp|P63279|UBC9\_HUMA | UBE2I | UBC9\_HUMAN SUMO-conjugating enzyme UBC9 | 1 |
| sp|O75436|VP26A\_HUM | VPS26A | VP26A\_HUMAN Vacuolar protein sorting-associated protein 26A | 1 |
| sp|Q16864-2|VATF\_HU | ATP6V1F | VATF\_HUMAN Isoform 2 of V-type proton ATPase subunit F | 1 |
| sp|Q66PJ3|AR6P4\_HUM | ARL6IP4 | AR6P4\_HUMAN ADP-ribosylation factor-like protein 6-interacting protei | 1 |
| sp|Q9NWT8|AKIP\_HUM | AURKAIP1 | AKIP\_HUMAN Aurora kinase A-interacting protein | 1 |
| sp|P46013|KI67\_HUMA | MKI67 | KI67\_HUMAN Antigen KI-67 | 1 |
| sp|Q92541|RTF1\_HUMA | RTF1 | RTF1\_HUMAN RNA polymerase-associated protein RTF1 homolog | 1 |
| sp|P02768|ALBU\_HUMA | ALB | ALBU\_HUMAN\_contaminant Serum albumin | 1 |
| sp|Q9UKX7|NUP50\_HU | NUP50 | NUP50\_HUMAN Nuclear pore complex protein Nup50 | 1 |
| sp|O43809|CPSF5\_HUM | NUDT21 | CPSF5\_HUMAN Cleavage and polyadenylation specificity factor subunit | 1 |
| tr|M0QZM1|M0QZM1\_ | HNRNPM | M0QZM1\_HUMAN Heterogeneous nuclear ribonucleoprotein M (Fragm | 1 |
| sp|O00178|GTPB1\_HUM | GTPBP1 | GTPB1\_HUMAN GTP-binding protein 1 | 1 |
| sp|Q9NW07|ZN358\_HU | ZNF358 | ZN358\_HUMAN Zinc finger protein 358 | 1 |
| sp|O75390|CISY\_HUMA | CS | CISY\_HUMAN Citrate synthase, mitochondria | 1 |
| sp|Q9BRJ7|SDOS\_HUM | NUDT16L1 | SDOS\_HUMAN Protein syndesmos | 1 |

## Slide 26
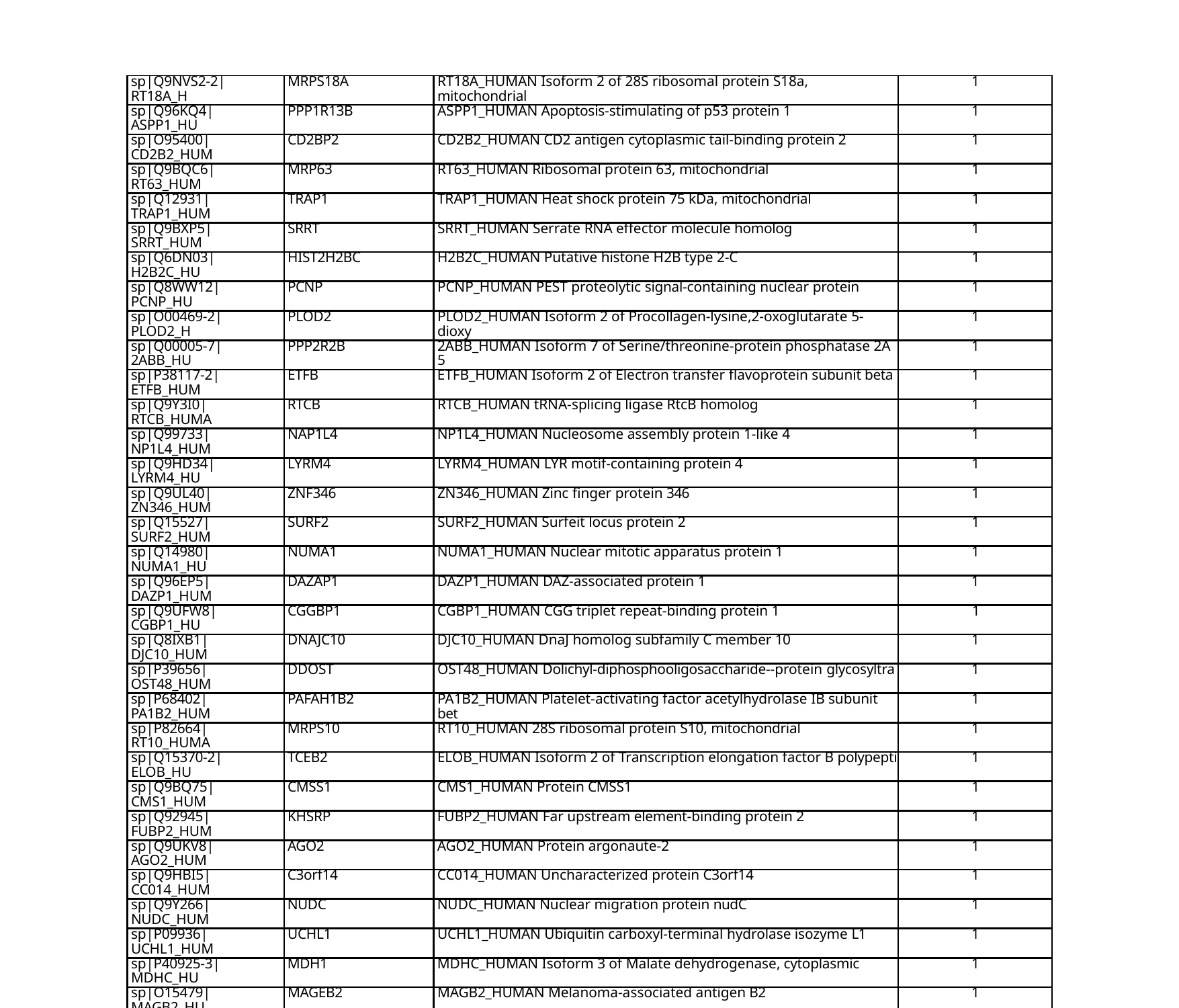

| sp|Q9NVS2-2|RT18A\_H | MRPS18A | RT18A\_HUMAN Isoform 2 of 28S ribosomal protein S18a, mitochondrial | 1 |
| --- | --- | --- | --- |
| sp|Q96KQ4|ASPP1\_HU | PPP1R13B | ASPP1\_HUMAN Apoptosis-stimulating of p53 protein 1 | 1 |
| sp|O95400|CD2B2\_HUM | CD2BP2 | CD2B2\_HUMAN CD2 antigen cytoplasmic tail-binding protein 2 | 1 |
| sp|Q9BQC6|RT63\_HUM | MRP63 | RT63\_HUMAN Ribosomal protein 63, mitochondrial | 1 |
| sp|Q12931|TRAP1\_HUM | TRAP1 | TRAP1\_HUMAN Heat shock protein 75 kDa, mitochondrial | 1 |
| sp|Q9BXP5|SRRT\_HUM | SRRT | SRRT\_HUMAN Serrate RNA effector molecule homolog | 1 |
| sp|Q6DN03|H2B2C\_HU | HIST2H2BC | H2B2C\_HUMAN Putative histone H2B type 2-C | 1 |
| sp|Q8WW12|PCNP\_HU | PCNP | PCNP\_HUMAN PEST proteolytic signal-containing nuclear protein | 1 |
| sp|O00469-2|PLOD2\_H | PLOD2 | PLOD2\_HUMAN Isoform 2 of Procollagen-lysine,2-oxoglutarate 5-dioxy | 1 |
| sp|Q00005-7|2ABB\_HU | PPP2R2B | 2ABB\_HUMAN Isoform 7 of Serine/threonine-protein phosphatase 2A 5 | 1 |
| sp|P38117-2|ETFB\_HUM | ETFB | ETFB\_HUMAN Isoform 2 of Electron transfer flavoprotein subunit beta | 1 |
| sp|Q9Y3I0|RTCB\_HUMA | RTCB | RTCB\_HUMAN tRNA-splicing ligase RtcB homolog | 1 |
| sp|Q99733|NP1L4\_HUM | NAP1L4 | NP1L4\_HUMAN Nucleosome assembly protein 1-like 4 | 1 |
| sp|Q9HD34|LYRM4\_HU | LYRM4 | LYRM4\_HUMAN LYR motif-containing protein 4 | 1 |
| sp|Q9UL40|ZN346\_HUM | ZNF346 | ZN346\_HUMAN Zinc finger protein 346 | 1 |
| sp|Q15527|SURF2\_HUM | SURF2 | SURF2\_HUMAN Surfeit locus protein 2 | 1 |
| sp|Q14980|NUMA1\_HU | NUMA1 | NUMA1\_HUMAN Nuclear mitotic apparatus protein 1 | 1 |
| sp|Q96EP5|DAZP1\_HUM | DAZAP1 | DAZP1\_HUMAN DAZ-associated protein 1 | 1 |
| sp|Q9UFW8|CGBP1\_HU | CGGBP1 | CGBP1\_HUMAN CGG triplet repeat-binding protein 1 | 1 |
| sp|Q8IXB1|DJC10\_HUM | DNAJC10 | DJC10\_HUMAN DnaJ homolog subfamily C member 10 | 1 |
| sp|P39656|OST48\_HUM | DDOST | OST48\_HUMAN Dolichyl-diphosphooligosaccharide--protein glycosyltra | 1 |
| sp|P68402|PA1B2\_HUM | PAFAH1B2 | PA1B2\_HUMAN Platelet-activating factor acetylhydrolase IB subunit bet | 1 |
| sp|P82664|RT10\_HUMA | MRPS10 | RT10\_HUMAN 28S ribosomal protein S10, mitochondrial | 1 |
| sp|Q15370-2|ELOB\_HU | TCEB2 | ELOB\_HUMAN Isoform 2 of Transcription elongation factor B polypepti | 1 |
| sp|Q9BQ75|CMS1\_HUM | CMSS1 | CMS1\_HUMAN Protein CMSS1 | 1 |
| sp|Q92945|FUBP2\_HUM | KHSRP | FUBP2\_HUMAN Far upstream element-binding protein 2 | 1 |
| sp|Q9UKV8|AGO2\_HUM | AGO2 | AGO2\_HUMAN Protein argonaute-2 | 1 |
| sp|Q9HBI5|CC014\_HUM | C3orf14 | CC014\_HUMAN Uncharacterized protein C3orf14 | 1 |
| sp|Q9Y266|NUDC\_HUM | NUDC | NUDC\_HUMAN Nuclear migration protein nudC | 1 |
| sp|P09936|UCHL1\_HUM | UCHL1 | UCHL1\_HUMAN Ubiquitin carboxyl-terminal hydrolase isozyme L1 | 1 |
| sp|P40925-3|MDHC\_HU | MDH1 | MDHC\_HUMAN Isoform 3 of Malate dehydrogenase, cytoplasmic | 1 |
| sp|O15479|MAGB2\_HU | MAGEB2 | MAGB2\_HUMAN Melanoma-associated antigen B2 | 1 |
| sp|P02662|CASA1\_BOVI | CSN1S1 | CASA1\_BOVIN\_contaminant Alpha-S1-casein | 1 |
| sp|Q08380|LG3BP\_HUM | LGALS3BP | LG3BP\_HUMAN Galectin-3-binding protein | 1 |
| sp|Q9P016|THYN1\_HU | THYN1 | THYN1\_HUMAN Thymocyte nuclear protein 1 | 1 |

## Slide 27
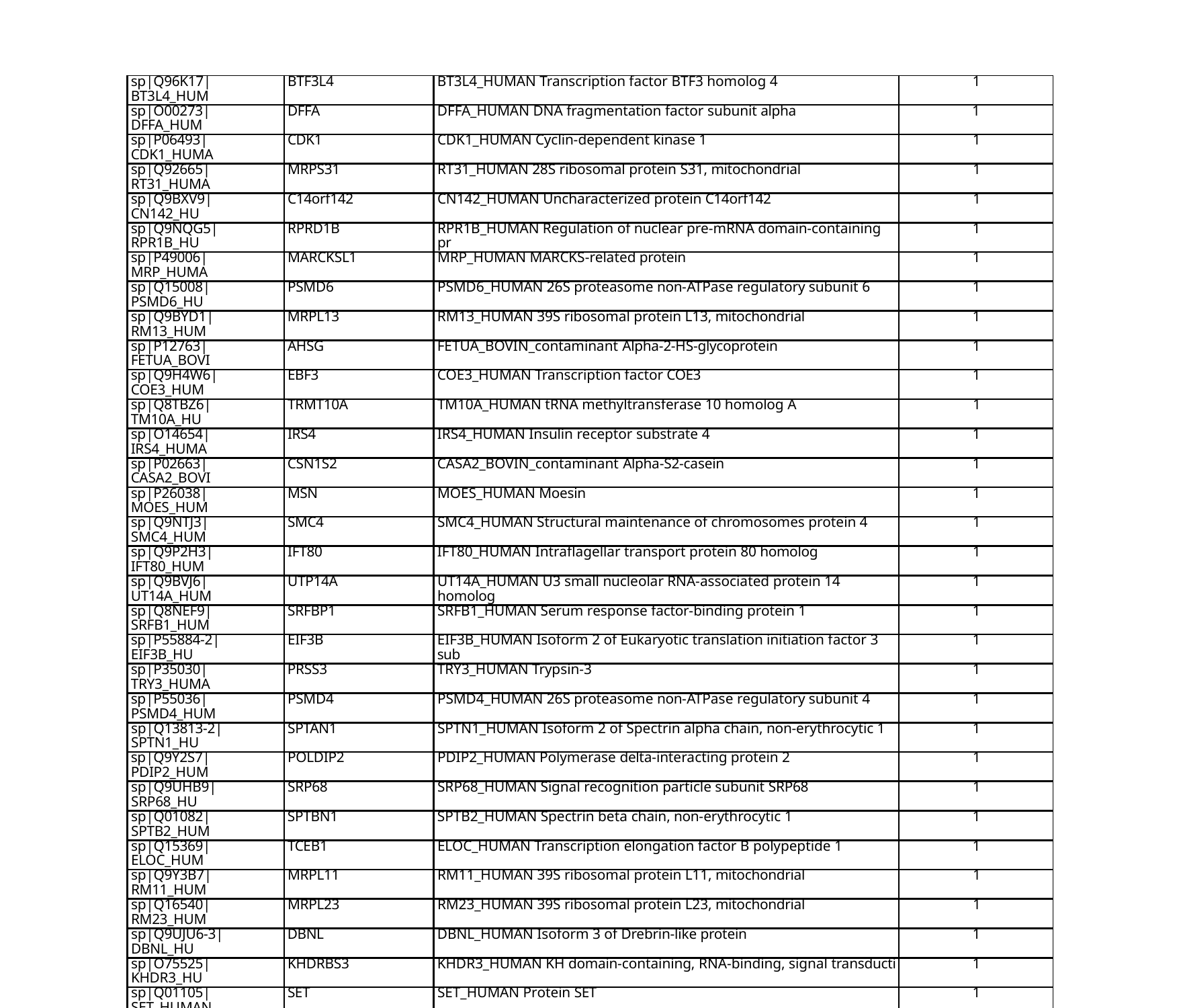

| sp|Q96K17|BT3L4\_HUM | BTF3L4 | BT3L4\_HUMAN Transcription factor BTF3 homolog 4 | 1 |
| --- | --- | --- | --- |
| sp|O00273|DFFA\_HUM | DFFA | DFFA\_HUMAN DNA fragmentation factor subunit alpha | 1 |
| sp|P06493|CDK1\_HUMA | CDK1 | CDK1\_HUMAN Cyclin-dependent kinase 1 | 1 |
| sp|Q92665|RT31\_HUMA | MRPS31 | RT31\_HUMAN 28S ribosomal protein S31, mitochondrial | 1 |
| sp|Q9BXV9|CN142\_HU | C14orf142 | CN142\_HUMAN Uncharacterized protein C14orf142 | 1 |
| sp|Q9NQG5|RPR1B\_HU | RPRD1B | RPR1B\_HUMAN Regulation of nuclear pre-mRNA domain-containing pr | 1 |
| sp|P49006|MRP\_HUMA | MARCKSL1 | MRP\_HUMAN MARCKS-related protein | 1 |
| sp|Q15008|PSMD6\_HU | PSMD6 | PSMD6\_HUMAN 26S proteasome non-ATPase regulatory subunit 6 | 1 |
| sp|Q9BYD1|RM13\_HUM | MRPL13 | RM13\_HUMAN 39S ribosomal protein L13, mitochondrial | 1 |
| sp|P12763|FETUA\_BOVI | AHSG | FETUA\_BOVIN\_contaminant Alpha-2-HS-glycoprotein | 1 |
| sp|Q9H4W6|COE3\_HUM | EBF3 | COE3\_HUMAN Transcription factor COE3 | 1 |
| sp|Q8TBZ6|TM10A\_HU | TRMT10A | TM10A\_HUMAN tRNA methyltransferase 10 homolog A | 1 |
| sp|O14654|IRS4\_HUMA | IRS4 | IRS4\_HUMAN Insulin receptor substrate 4 | 1 |
| sp|P02663|CASA2\_BOVI | CSN1S2 | CASA2\_BOVIN\_contaminant Alpha-S2-casein | 1 |
| sp|P26038|MOES\_HUM | MSN | MOES\_HUMAN Moesin | 1 |
| sp|Q9NTJ3|SMC4\_HUM | SMC4 | SMC4\_HUMAN Structural maintenance of chromosomes protein 4 | 1 |
| sp|Q9P2H3|IFT80\_HUM | IFT80 | IFT80\_HUMAN Intraflagellar transport protein 80 homolog | 1 |
| sp|Q9BVJ6|UT14A\_HUM | UTP14A | UT14A\_HUMAN U3 small nucleolar RNA-associated protein 14 homolog | 1 |
| sp|Q8NEF9|SRFB1\_HUM | SRFBP1 | SRFB1\_HUMAN Serum response factor-binding protein 1 | 1 |
| sp|P55884-2|EIF3B\_HU | EIF3B | EIF3B\_HUMAN Isoform 2 of Eukaryotic translation initiation factor 3 sub | 1 |
| sp|P35030|TRY3\_HUMA | PRSS3 | TRY3\_HUMAN Trypsin-3 | 1 |
| sp|P55036|PSMD4\_HUM | PSMD4 | PSMD4\_HUMAN 26S proteasome non-ATPase regulatory subunit 4 | 1 |
| sp|Q13813-2|SPTN1\_HU | SPTAN1 | SPTN1\_HUMAN Isoform 2 of Spectrin alpha chain, non-erythrocytic 1 | 1 |
| sp|Q9Y2S7|PDIP2\_HUM | POLDIP2 | PDIP2\_HUMAN Polymerase delta-interacting protein 2 | 1 |
| sp|Q9UHB9|SRP68\_HU | SRP68 | SRP68\_HUMAN Signal recognition particle subunit SRP68 | 1 |
| sp|Q01082|SPTB2\_HUM | SPTBN1 | SPTB2\_HUMAN Spectrin beta chain, non-erythrocytic 1 | 1 |
| sp|Q15369|ELOC\_HUM | TCEB1 | ELOC\_HUMAN Transcription elongation factor B polypeptide 1 | 1 |
| sp|Q9Y3B7|RM11\_HUM | MRPL11 | RM11\_HUMAN 39S ribosomal protein L11, mitochondrial | 1 |
| sp|Q16540|RM23\_HUM | MRPL23 | RM23\_HUMAN 39S ribosomal protein L23, mitochondrial | 1 |
| sp|Q9UJU6-3|DBNL\_HU | DBNL | DBNL\_HUMAN Isoform 3 of Drebrin-like protein | 1 |
| sp|O75525|KHDR3\_HU | KHDRBS3 | KHDR3\_HUMAN KH domain-containing, RNA-binding, signal transducti | 1 |
| sp|Q01105|SET\_HUMAN | SET | SET\_HUMAN Protein SET | 1 |
| sp|Q7Z7F7-2|RM55\_HU | MRPL55 | RM55\_HUMAN Isoform 2 of 39S ribosomal protein L55, mitochondrial | 1 |
| sp|Q1KMD3|HNRL2\_HU | HNRNPUL2 | HNRL2\_HUMAN Heterogeneous nuclear ribonucleoprotein U-like protei | 1 |
| sp|Q13442|HAP28\_HUM | PDAP1 | HAP28\_HUMAN 28 kDa heat- and acid-stable phosphoprotein | 1 |

## Slide 28
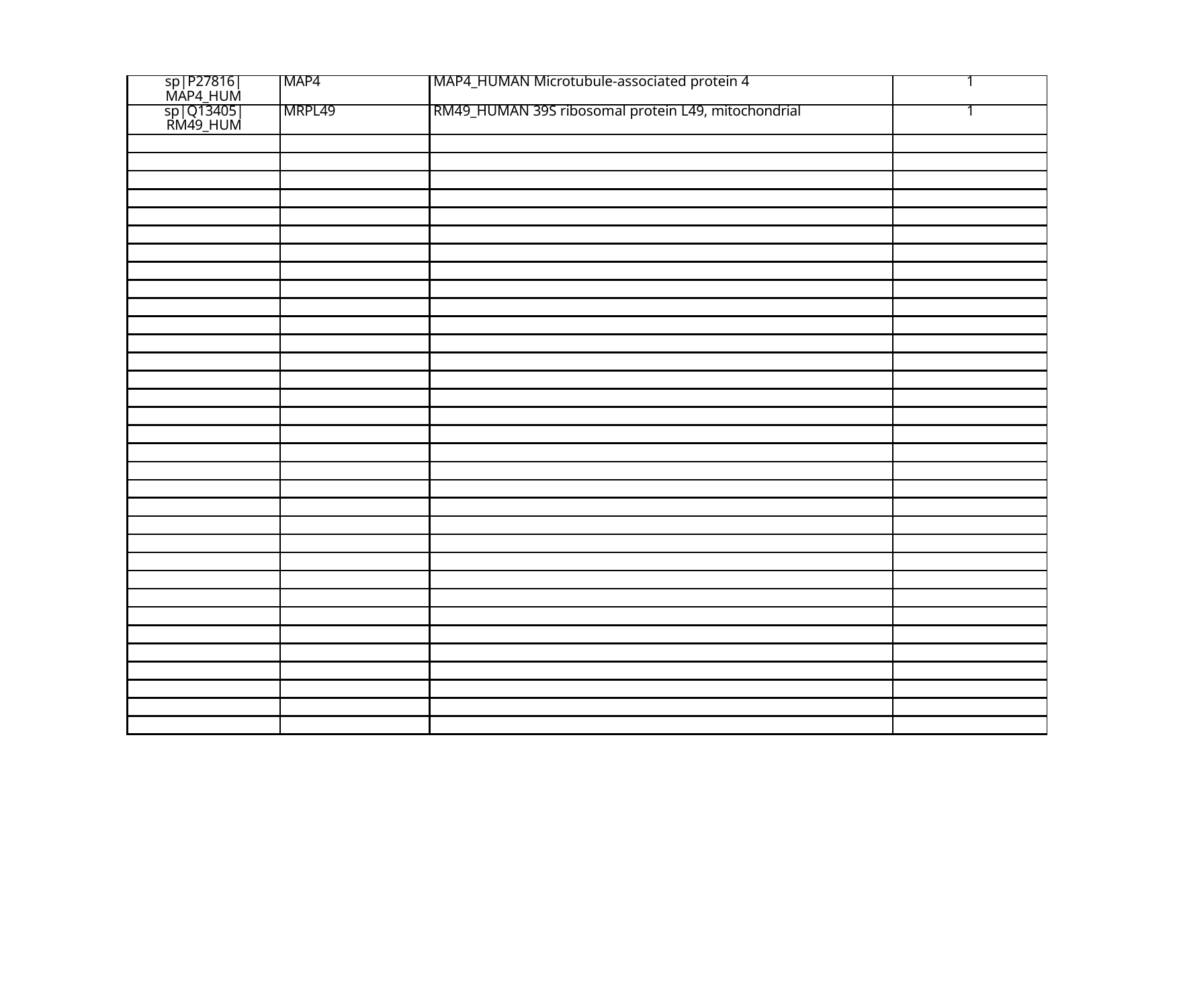

| sp|P27816|MAP4\_HUM | MAP4 | MAP4\_HUMAN Microtubule-associated protein 4 | 1 |
| --- | --- | --- | --- |
| sp|Q13405|RM49\_HUM | MRPL49 | RM49\_HUMAN 39S ribosomal protein L49, mitochondrial | 1 |
| | | | |
| | | | |
| | | | |
| | | | |
| | | | |
| | | | |
| | | | |
| | | | |
| | | | |
| | | | |
| | | | |
| | | | |
| | | | |
| | | | |
| | | | |
| | | | |
| | | | |
| | | | |
| | | | |
| | | | |
| | | | |
| | | | |
| | | | |
| | | | |
| | | | |
| | | | |
| | | | |
| | | | |
| | | | |
| | | | |
| | | | |
| | | | |
| | | | |
